# In vivo genome and base editing of a human *PCSK9* knock-in hypercholesterolemic mouse model

**DOI:** 10.1186/s12915-018-0624-2

**Published:** 2019-01-15

**Authors:** Alba Carreras, Luna Simona Pane, Roberto Nitsch, Katja Madeyski-Bengtson, Michelle Porritt, Pinar Akcakaya, Amir Taheri-Ghahfarokhi, Elke Ericson, Mikael Bjursell, Marta Perez-Alcazar, Frank Seeliger, Magnus Althage, Ralph Knöll, Ryan Hicks, Lorenz M. Mayr, Rosie Perkins, Daniel Lindén, Jan Borén, Mohammad Bohlooly-Y, Marcello Maresca

**Affiliations:** 1 Discovery Biology, Discovery Sciences, IMED Biotech Unit, AstraZeneca, Pepparedsleden 1, Mölndal, 43 183 Gothenburg, Sweden; 2 Advanced Medicines Safety, Drug Safety and Metabolism, IMED Biotech Unit, AstraZeneca, Gothenburg, Sweden; 3 Pathology Science, Drug Safety and Metabolism, IMED Biotech Unit, AstraZeneca, Gothenburg, Sweden; 4 Cardiovascular, Renal and Metabolism, IMED Biotech Unit, AstraZeneca, Gothenburg, Sweden; 5 Discovery Biology, Discovery Sciences, IMED Biotech Unit, AstraZeneca, Gothenburg, Sweden; 6 Department of Molecular and Clinical Medicine, University of Gothenburg, The Wallenberg Laboratory, Sahlgrenska University Hospital, Gothenburg, Sweden; 7 Present Address: Department of Molecular and Clinical Medicine, University of Gothenburg, The Wallenberg Laboratory, Sahlgrenska University Hospital, Gothenburg, Sweden; 8 Present Address: GE Healthcare Life Sciences, The Grove Centre, White Lion Road, Amersham, UK

**Keywords:** Hypercholesterolemia, PCSK9, Genome editing, Base editing, CRISPR-Cas9

## Abstract

**Background:**

Plasma concentration of low-density lipoprotein (LDL) cholesterol is a well-established risk factor for cardiovascular disease. Inhibition of proprotein convertase subtilisin/kexin type 9 (PCSK9), which regulates cholesterol homeostasis, has recently emerged as an approach to reduce cholesterol levels. The development of humanized animal models is an important step to validate and study human drug targets, and use of genome and base editing has been proposed as a mean to target disease alleles*.*

**Results:**

To address the lack of validated models to test the safety and efficacy of techniques to target human PCSK9, we generated a liver-specific human PCSK9 knock-in mouse model (hPCSK9-KI). We showed that plasma concentrations of total cholesterol were higher in hPCSK9-KI than in wildtype mice and increased with age. Treatment with evolocumab, a monoclonal antibody that targets human PCSK9, reduced cholesterol levels in hPCSK9-KI but not in wildtype mice, showing that the hypercholesterolemic phenotype was driven by overexpression of human PCSK9. CRISPR-Cas9-mediated genome editing of human PCSK9 reduced plasma levels of human and not mouse PCSK9, and in parallel reduced plasma concentrations of total cholesterol; genome editing of mouse Pcsk9 did not reduce cholesterol levels. Base editing using a guide RNA that targeted human and mouse PCSK9 reduced plasma levels of human and mouse PCSK9 and total cholesterol. In our mouse model, base editing was more precise than genome editing, and no off-target editing nor chromosomal translocations were identified.

**Conclusions:**

Here, we describe a humanized mouse model with liver-specific expression of human PCSK9 and a human-like hypercholesterolemia phenotype, and demonstrate that this mouse can be used to evaluate antibody and gene editing-based (genome and base editing) therapies to modulate the expression of human PCSK9 and reduce cholesterol levels. We predict that this mouse model will be used in the future to understand the efficacy and safety of novel therapeutic approaches for hypercholesterolemia.

**Electronic supplementary material:**

The online version of this article (10.1186/s12915-018-0624-2) contains supplementary material, which is available to authorized users.

## Background

The circulating concentration of low-density lipoprotein (LDL) cholesterol is a well-established risk factor for cardiovascular disease [1, 2]. Although statins are currently the main treatment for hypercholesterolemia, many patients are statin intolerant [3–5] and inhibition of proprotein convertase subtilisin/kexin type 9 (PCSK9) has recently emerged as an alternative or parallel approach to reduce cholesterol levels [6–9]. PCSK9 plays a key role in cholesterol homeostasis by directing membrane-bound LDL receptors to lysosomal degradation [10, 11]. Individuals with loss-of-function mutations in *PCSK9* have ~ 30% lower levels of LDL cholesterol than the general population [12, 13]. Anti-human PCSK9 monoclonal antibodies [14–17] and PCSK9 siRNAs [18] have been developed and used in clinical trials to reduce plasma LDL cholesterol levels [19–21]. However, although both these treatments can achieve therapeutic benefit, their effects are short lived and chronic administration is required.

It is today possible to permanently alter the human genome using gene-editing techniques. Recent studies have used CRISPR-Cas9 (clustered regularly interspaced short palindromic repeats [CRISPR]-CRISPR-associated protein 9) to disrupt *PCSK9* in mouse and human hepatocytes in vivo, leading to reduced plasma concentrations of PCSK9 and cholesterol [22, 23]. In these studies, CRISPR-Cas9 generated a double-strand break in the DNA that was repaired by non-homologous end-joining (NHEJ) [24], a method that is error prone and may produce off-target mutations, thus limiting its use in humans. Base editing, which uses cytidine or adenosine deaminases fused to dead Cas9 or Cas9 nickase, has been proposed as a safer and more precise alternative to standard genome editing [25–27]. These programmable deaminases, called base editors, have been reported to specifically promote cytidine to thymidine (C to T) transitions (G to A on the opposite strand) at guide RNA target sites in mammalian cells, mouse embryos, and adult mice [25, 26, 28–30] with consistently comparable efficiency to CRISPR-Cas9 but with increased precision in single nucleotide substitutions [31]. A recent study used base editing in adult mice to disrupt mouse *Pcsk9*, which resulted in reduced plasma concentrations of PCSK9 and cholesterol with no evidence of off-target mutagenesis [29]. However, base editing of human *PCSK9* in vivo has not previously been attempted.

The development of animal models of human diseases is an important step to validate and study human drug targets. When testing genetic and antibody therapies in vivo, a high level of human homology is needed, and humanized mouse models are therefore particularly relevant in this context. Here, we describe a mouse model with liver-specific expression of human *PCSK9* and a human-like hypercholesterolemia phenotype on chow diet. We demonstrate that this mouse can be used as a treatment model to evaluate antibody and gene editing-based (genome and base editing) therapies to modulate human PCSK9 levels. We predict that this mouse model will be used in the future to understand the efficacy and safety of novel therapeutic approaches for hypercholesterolemia.

## Results

### Generation and characterization of the humanized hypercholesterolemic mouse model

**Fig. 1:**
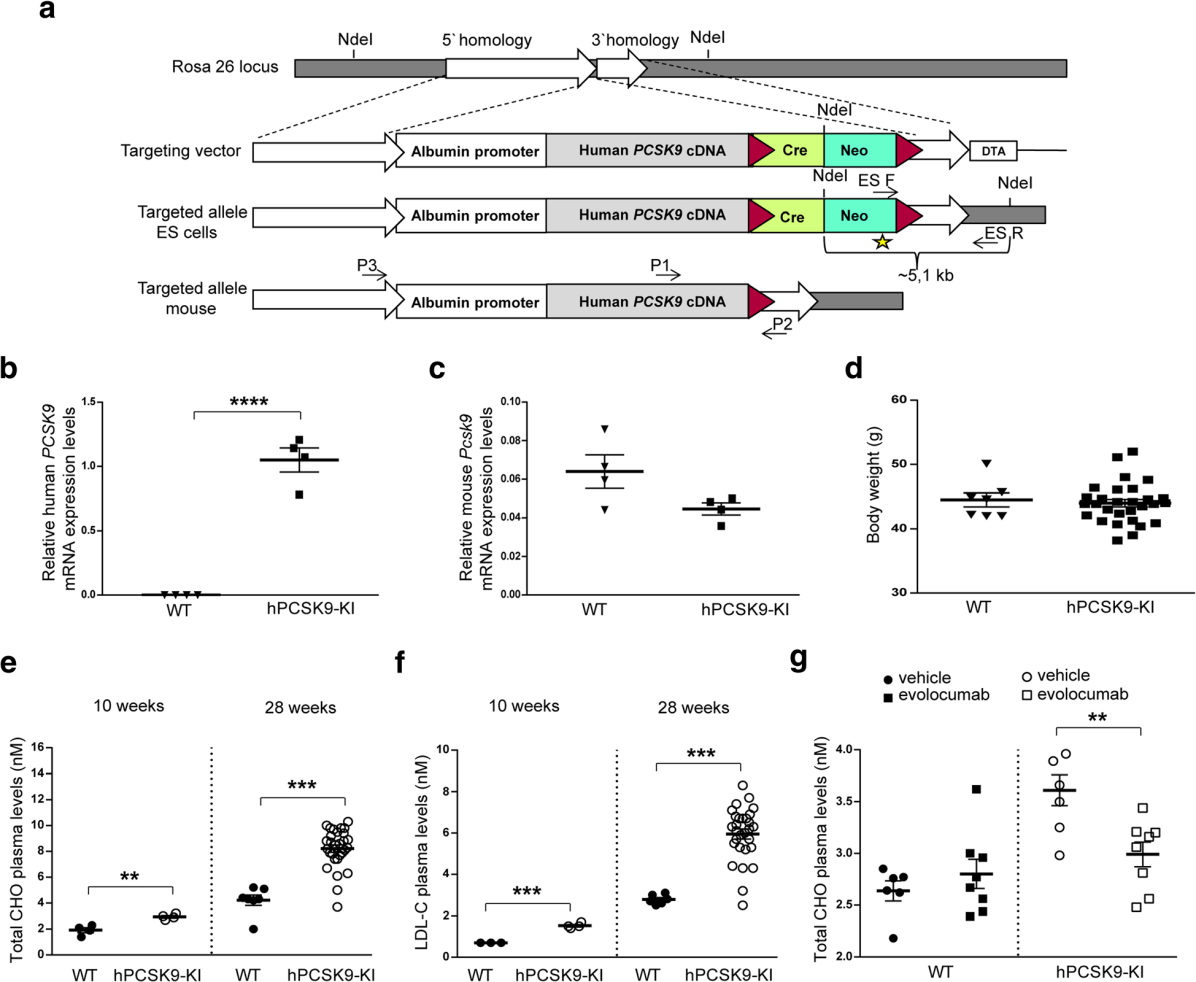
Generation and characterization of the human *PCSK9* knock-in mouse model (hPCSK9-KI). **a** Generation of the hPCSK9-KI mouse. A human *PCSK9*-cDNA expression cassette was inserted into the mouse *Rosa26* locus. Red triangles indicate loxP sites flanking the Cre-Neo cassette in the targeting vector. The *Neo* gene was used for selection of positive clones, and the *Diphtheria toxin A* fragment gene (DTA) was used for negative selection of clones with random integrations of the transgene. Arrows indicate the genotyping primers; NdeI indicates the restriction enzyme site used for the neomycin probe (star) in Southern blot analysis. **b**, **c** Human *PCSK9* and mouse *Pcsk9* mRNA expression levels relative to β-actin in the liver from 10-week-old hPCSK9-KI mice and WT littermates (*n* = 4 per group). **d** Body weight in 28-week-old WT mice (*n* = 7) and hPCSK9-KI mice (*n* = 29). **e**, **f** Plasma concentrations of total cholesterol (CHO) and LDL cholesterol (LDL-C) in hPCSK9-KI mice and WT littermates at 10 and 28 weeks of age (*n* = 4 at 10 weeks; *n* = 7 for the WT group and *n* = 29 for the hPCSK9-KI group at 28 weeks). **g** Plasma concentrations of total cholesterol (CHO) 24 h after treatment with 10 mg/kg evolocumab in 13-week-old hPCSK9-KI mice and their WT littermates (*n* = 6 for the WT group and *n* = 8 for the hPCSK9-KI group). Student’s *t* test analysis was performed to evaluate the differences between WT and hPCSK9-KI mice. Values are presented as group means ± SEM. WT, wild type C57BL/6N. hPCSK9-KI, human PCSK9 inserted into Rosa26 locus in C57BL/6N. **p < 0.001, ***p < 0.0001, and ****p < 0.00001

To obtain a permanent model of hypercholesterolemia, we generated a knock-in (KI) mouse with liver-specific expression of human *PCSK9*, here termed hPCSK9-KI (Fig. 1a). Expression of human *PCSK9* was driven by the albumin promoter, which directs the transgene expression specifically in the liver, whereas mouse *Pcsk9* expression remained under control of its endogenous promoter. Because albumin is the most abundant serum protein produced by the liver, we reasoned that linking the expression of human *PCSK9* to this promoter would induce a dyslipidemia similar to that observed in humans carrying *PCSK9* gain-of function mutations [13, 32–37].

Immunohistochemical analysis confirmed liver-specific expression of human *PCSK9* in 10-week-old hPCSK9-KI mice (Additional file 1: Figure S1). Human *PCSK9* was expressed in the liver from hPCSK9-KI but not from their wildtype (WT) littermates (Fig. 1b) whereas expression of endogenous mouse *Pcsk9* mRNA was comparable in the liver from hPCSK9-KI and WT mice (Fig. 1c). The hPCSK9-KI mice reached adulthood with a development similar to their WT littermates, and no differences were observed in body weight between these genotypes at 28 weeks of age (Fig. 1d). Plasma levels of total cholesterol and LDL cholesterol were significantly higher in the hPCSK9-KI mice compared with their WT littermates at both 10 and 28 weeks of age (Fig. 1e, f and Additional file 2: Table S1). Together, these data demonstrate a clear PCSK9-driven hypercholesterolemic phenotype that worsens with age, similar to the lipid profile seen in humans with hypercholesterolemia [32, 34–37].

To verify that the hypercholesterolemic phenotype of hPCSK9-KI mice is caused by expression of human *PCSK9*, we administered 10 mg/kg evolocumab (a monoclonal antibody that inhibits human PCSK9) or vehicle subcutaneously to WT and hPCSK9-KI mice. Total plasma cholesterol levels in WT mice were not affected by evolocumab treatment, but were significantly lower in evolocumab-treated hPCSK9-KI mice compared with vehicle-treated hPCSK9-KI mice 24 h after injection (Fig. 1g). These data support the hypothesis that the hypercholesterolemic phenotype observed in our transgenic model is dictated by the liver-specific expression of human *PCSK9*.

### Cas9-mediated in vivo editing of human *PCSK9*

**Fig. 2:**
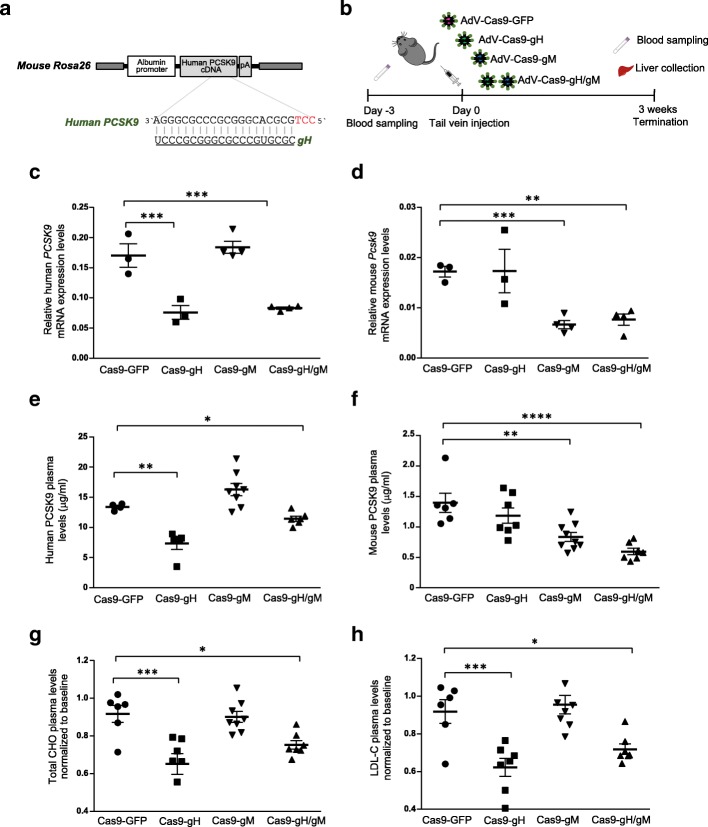
Cas9-mediated in vivo knock-out of human *PCSK9* and mouse *Pcsk9* in hPCSK9-KI mice. **a** Targeted region at human *PCSK9* locus. The guide RNA gH targets the human *PCSK9* cDNA knocked into the mouse R26 locus. **b** Experimental design: 28-week-old hPCSK9-KI mice were transduced with adenoviral vectors (AdV) encoding Cas9 together with gH, gM (targeting mouse *Pcsk9*), both gH and gM (gH/gM), or GFP as control. Blood was collected 3 days before injection (baseline); the blood and liver were collected 3 weeks after injection. **c**, **d** Human *PCSK9* and mouse *mPcsk9* mRNA expression levels relative to β-actin in the liver from mice 3 weeks after Cas9 treatment (*n* = 3 for the Cas9-GFP and Cas9-gH groups and *n* = 4 for the Cas9-gM and Cas9gH/gM groups). **e**, **f** Plasma concentrations of human and mouse PCSK9 proteins 3 weeks after Cas9 treatment (*n* = 6 for the Cas9-GFP group; *n* = 7 for the Cas9-gH and Cas9gH/gM groups; *n* = 8 for the Cas9-gM group). **g**, **h** Plasma concentrations of total cholesterol (CHO) and LDL cholesterol (LDL-C) 3 weeks after Cas9 treatment (normalized to plasma concentrations at baseline; *n* = 6 for the Cas9-GFP group; *n* = 7 for the Cas9-gH and Cas9gH/gM groups; *n* = 8 for the Cas9-gM group). Data were analyzed with one-way ANOVA and Dunnett’s correction for multiple testing. Values are presented as group means ± SEM. *p < 0.05; **p < 0.001; ***p < 0.0001

To determine the effect of genomic targeting of human *PCSK9* by CRISPR-Cas9 in our hPCSK9-KI mouse model, we designed a novel *Streptococcus pyogenes* Cas9 guide RNA (here termed gH) targeting the coding sequence of exon 1 within the human *PSCK9* gene (Fig. 2a and Additional file 3: Figure S2a). As a control, we used a previously described and validated guide RNA (here termed gM) to target mouse *Pcsk9* [22] (Additional file 3: Figure S2a).

We first co-expressed gH with Cas9 in human HEK293T cells to test the specificity and efficacy of gH in targeting the human *PCSK9* locus in vitro. Using a well-validated method of CRISPR off-target detection (GUIDE-seq) [38], we showed that the on-target site was represented in the majority of read counts; two off-target sites were identified at intergenic and intron regions of the genome, but these had very low read counts (Additional file 4: Table S2). By using the Surveyor assay on genomic DNA from transfected cells, we demonstrated active cleavage within the *PCSK9* targeted region (Additional file 3: Figure S2b).

Adenovirus is one of the most effective means of delivering genes in vivo, and particularly to the liver. Here, we injected adenoviral vectors encoding Cas9 together with gH, gM, both gH and gM (gH/gM), or, as a negative control, GFP into 28-week-old hPCSK9-KI mice, randomized into age-matched groups on the basis of body weight (Fig. 2b). hPCSK9-KI mice transduced with gH/gM received half the dose of gH compared to the mice transduced with gH alone. Three weeks after transduction, we observed efficient cleavage (as determined by the Surveyor assay) of human *PCSK9* or mouse *Pcsk9* in liver tissue from hPCSK9-KI mice treated with Cas9-gH or Cas9-gM, respectively, and of both human *PCSK9* and mouse *Pcsk9* in liver tissue from hPCSK9-KI mice treated with Cas9-gH/gM (Additional file 3: Figure S2c). No genetic disruption was observed in hPCSK9-KI mice treated with the negative control vector (Additional file 3: Figure S2c).

Three weeks after treatment, we observed significant reductions in hepatic mRNA expression and circulating protein levels of human PCSK9 in Cas9-gH-treated mice, of mouse PCSK9 in Cas9-gM-treated mice, and of both human and mouse PCSK9 in Cas9-gH/gM-treated mice compared with levels in Cas9-GFP-injected mice (Fig. 2c–f). In parallel with the high expression of human versus mouse *PCSK9* mRNA, protein levels of human PCSK9 were notably higher than those of mouse PCSK9 in Cas9-GFP-injected hPCSK9-KI mice (Fig. 2c–f). Histopathological and immunohistochemical analyses of the liver confirmed that downregulation of human and/or mouse PCSK9 protein levels was specific to hPCSK9-KI mice treated with the corresponding Cas9, and there was no cross reactivity (Additional file 5: Figure S3). In agreement with the main role of human PCSK9 in driving the hypercholesterolemic phenotype, staining of LDL-receptor-positive cells was highest in the liver from hPCSK9 mice transduced with gH (Additional file 5: Figure S3).

In parallel with the reduction in human PCSK9 levels, we observed significant reductions in total plasma cholesterol and LDL cholesterol levels in hPCSK9-KI mice 3 weeks after treatment with Cas9-gH or Cas9-gH/gM compared with Cas9-GFP treatment (Fig. 2g, h). No reduction in total plasma cholesterol or LDL cholesterol levels was observed in hPCSK9-KI mice injected with Cas9-gM (Fig. 2g, h). Although this result is in contrast to a previous study that reported a reduction in cholesterol and LDL cholesterol in WT mice treated with Cas9-gM [22], this discrepancy can be explained by the fact that human *PCSK9* expression appears to be higher than mouse *Pcsk9* expression in our mouse model and therefore ablation of mouse *Pcsk9* would not be expected to have much effect on cholesterol levels. Overall, these data confirm the key role of human PCSK9 in driving the hypercholesterolemic phenotype observed in the hPCSK9-KI model.

To assess whether our hPCSK9-KI mouse model could be used as a tool to investigate the effect of genome editing in younger mice, we tested Cas9-gH in hPCSK9-KI mice that were 10 weeks old when treatment was initiated. Indeed, we also observed efficient cleavage (as determined by the Surveyor assay) at the human *PCSK9* locus (Additional file 6: Figure S4a) and significant reductions in human PCSK9 protein and total cholesterol plasma levels 3 weeks after Cas9-gH treatment in hPCSK9-KI mice (Additional file 6: Figure S4b, d). Histopathological and immunohistochemical analysis of the liver in hPCSK9-KI mice confirmed downregulation of human PCSK9 and increased staining of LDL-receptor-positive cells in Cas9-gH-compared with Cas9-GFP-injected mice (Additional file 6: Figure S4e).

We also observed a non-significant reduction in mouse PCSK9 protein 3 weeks after Cas9-gH treatment in hPCSK9-KI mice (Additional file 6: Figure S4c). Given the number of mismatches between gH and the mouse gene (Additional file 3: Figure S2a), this effect is more likely explained by the variability of mRNA and protein levels between mice than by gH binding to the mouse locus. However, we cannot exclude the possibility that an altered level of human *PCSK9* after Cas9-gH treatment (Additional file 6: Figure S4b), resulting in an increase of LDL-receptor signaling (Additional file 6: Figure S4e), led to an indirect reduction of mouse *Pcsk9* expression. Of note, Cas9-gH treatment did not significantly affect the levels of mouse PCSK9 protein or total plasma cholesterol in WT mice (Additional file 6: Figure S4c, d), confirming the specificity of gH for the human locus.

### BE3-mediated in vivo editing of human *PCSK9*

**Fig. 3:**
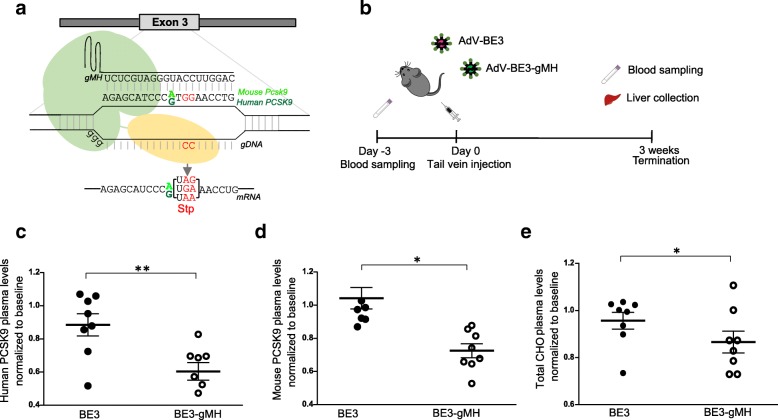
Base editor-mediated in vivo knock-out of human *PCSK9* and mouse *Pcsk9* in hPCSK9-KI mice. **a** Targeted regions at human *PCSK9* and mouse *Pcsk9* loci. The gMH guide RNA has perfect complementarity to the mouse *Pcsk9* locus and a single nucleotide mismatch to the human gene (A vs G in position 11 of the protospacer). The nucleotides substituted by base editing are shown in red. **b** Experimental design: 10-week-old hPCSK9-KI mice were transduced with adenoviral vectors (AdV) encoding BE3 alone (control) or BE3 together with gMH. Blood was collected 3 days before injection (baseline); the blood and liver were collected 3 weeks after injection. **c**, **d** Plasma concentrations of human and mouse PCSK9 proteins 3 weeks after BE3 treatment (normalized to plasma concentrations at baseline; *n* = 8 per group). **e** Plasma concentrations of total cholesterol (CHO) 3 weeks after BE3 treatment (normalized to plasma concentrations at baseline; *n* = 8 per group). Data were pooled from two independent experiments and analyzed with one-way ANOVA and Dunnett’s correction for multiple testing. Values are presented as group means ± SEM. *p < 0.05; **p < 0.001

In contrast to standard CRISPR-Cas9, base editors (cytidine deaminases fused to a dead Cas9 or Cas9 nickase) can promote cytidine to thymidine (C to T) transitions at specific target sites without generating DNA double-strand breaks [25, 26, 31, 39]. Base editing has previously been used to introduce nonsense mutations in *Pcsk9* in the mouse liver in vivo, resulting in reduced circulating PCSK9 protein and cholesterol levels with no evidence of off-target mutagenesis [29]. This earlier study used the widely used third-generation base editor BE3 and a guide RNA targeting codon W159 (TGG) within the mouse *Pcsk9* locus [29]. This guide RNA has perfect complementarity to the mouse *Pcsk9* locus and one nucleotide mismatch to the human gene (Fig. 3a); we used this guide (here termed gMH) to target both mouse *Pcsk9* and human *PCSK9* and in our hPCSK9-KI mouse model. Of note, W159 is also proximal to a loss-of-function variant of human *PCSK9* that has previously been described as a nonsense allele protective against hypercholesterolemia and coronary heart disease [40].

To test the efficacy of gMH in targeting the human *PCSK9* locus, we co-expressed gMH with BE3 in human HEK293T cells. Deep sequencing of genomic DNA from transfected cells showed that the majority of single nucleotide substitutions at the *PCSK9* locus were the targeted C to T transitions (G to A on the opposite strand) (Additional file 7: Figure S5a). Next, to assess whether efficient base editing of human *PCSK9* could be achieved in vivo, we injected adenoviral vectors encoding BE3 together with gMH or BE3 alone as control into 10-week-old hPCSK9-KI mice, randomized into two groups on the basis of body weight (Fig. 3b). Three weeks after transduction, deep sequencing of genomic DNA from liver tissue of hPCSK9-KI mice treated with BE3-gMH showed that most of the single nucleotide substitutions in the human *PCSK9* and mouse *Pcsk9* were the targeted C to T transitions (G to A on the opposite strand) (Additional file 7: Figure S5b).

We observed significant reductions in the levels of circulating human and mouse PCSK9 protein in BE3-gMH treated hPCSK9-KI mice compared with control BE3-injected littermates 3 weeks after injection (Fig. 3c, d). Histopathological and immunohistochemical analysis of the liver confirmed reduced levels of human PCSK9 protein and increased staining of LDL-receptor-positive cells in hPCSK9-KI mice treated with BE3-gMH (Additional file 8: Figure S6). Furthermore, we observed significant reductions in the levels of plasma total cholesterol in BE3-gMH treated hPCSK9-KI mice compared with control BE3-injected littermates 3 weeks after injection (Fig. 3e).

### Comparison of Cas9- and BE3-mediated in vivo editing of human *PCSK9*

To directly compare the mutagenic effect of BE3 and Cas9 treatments in vivo, we analyzed genomic DNA from liver tissue by deep sequencing 3 weeks after injecting 10-week-old hPCSK9-KI mice with adenoviral vectors encoding Cas9-gMH and BE-gMH. We chose gMH as the guide RNA for this comparison as it is suitable for use with both methods whereas gH cannot be used for base editing.

**Fig. 4:**
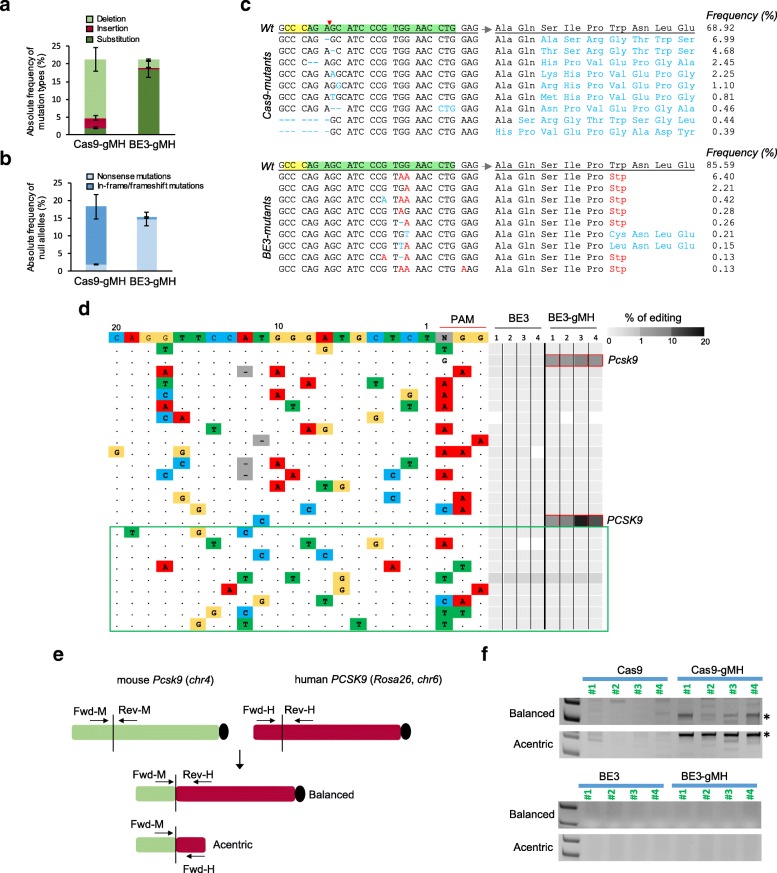
In vivo comparison of Cas9 and base editor mutagenic effects in hPCSK9-KI mice. Ten-week-old hPCSK9-KI mice were transduced with adenoviral vectors encoding Cas9-gMH or BE3-gMH. Genomic DNA from mouse liver was analyzed by deep sequencing (**a**–**d**) and PCR (**f**) 3 weeks after treatment. Absolute frequency of (**a**) mutation types and **(b)** null alleles in hPCSK9-KI mice treated with Cas9-gMH (*n* = 6) or BE3-gMH (*n* = 8). **c** Representative alignments of Cas9- and BE3-induced mutant alleles and their corresponding predicted protein sequences. Protospacer adjacent motif and gMH sequence are highlighted in yellow and green, respectively. The red arrowhead indicates Cas9 cutting site. **d** Analysis of 15 off-target sites previously identified for Cas9-gMH by CIRCLE-seq [41] and 9 off-target sites predicted bioinformatically (boxed in green). Mutation frequencies determined by amplicon seq are presented as heat maps for the mouse *Pcsk9* on-target site and the 24 off-targets, including human *PCSK9*. For each site, mismatches relative to the on-target site are shown as colored boxes and bases in the spacer sequence are numbered from 1 (most PAM-proximal) to 20 (most PAM-distal). Each box in the heatmap represents a single sequencing experiment. Sites that were significantly different between groups treated with BE3-gMH and BE3 alone are highlighted with a red outline around the boxes (*n* = 4 per group). **e** Schematic of balanced and acentric inter-chromosomal translocations occurring between the mouse *Pcsk9* locus and the human *PCSK9* locus. **f** PCR on genomic DNA from liver tissue of hPCSK9-KI mice treated with Cas9-gMH or BE3-gMH (and Cas9-GFP and BE3 as controls) was amplified to assess for translocation events. A PCR band of 327 bp or 492 bp (indicated by *) can only be amplified if a translocation (balanced or acentric, respectively) between the mouse *Pcsk9* locus and the human *PCSK9* locus has occurred

Overall, Cas9-gMH and BE3-gMH treatments resulted in similar editing frequencies at the human *PCSK9* locus (range 10.2–31.5% and 11.1–34.9%, respectively). By contrast, the editing frequency was higher after Cas9-gMH treatment compared to BE3-gMH treatment at the mouse *Pcsk9* locus (range 13.7–38.2% and 5.5–14.4%, respectively). At both the human and mouse loci, single-base changes accounted for 90% of BE3-gMH-associated mutations but for less than 10% of Cas9-gMH-associated mutations (Fig. 4a). Accordingly, Cas9-gMH treatment mostly produced in-frame and frameshift mutations whereas BE3-gMH treatment mostly generated nonsense mutations (Fig. 4b and Additional file 9: Table S3). In accordance with the absolute editing efficiency, the frequency of total null alleles at the human *PCSK9* locus after Cas9-gMH or BE3-gMH treatment was similar (range 5.7–25.6% and 8.2–27.3%, respectively) but higher after Cas9-gMH treatment compared to BE3-gMH treatment at the mouse *Pcsk9* locus (range 7.4–27.4% and 3.1–13.5%, respectively). However, the relative percentages of total null alleles were similar for the two treatments (72.6% and 73.1% for Cas9-gMH and BE3-gMH respectively at the human *PCSK9* locus and 65.9% and 69.6% for Cas9-gMH and BE3-gMH respectively at the mouse *Pcks9* locus; Additional file 9: Table S3). Of note, the number of allelic variants was significantly lower after BE3-gMH treatment than after Cas9-gMH treatment (Fig. 4c and Additional file 10: Table S4). Moreover, in contrast to Cas9-gMH treatment, which was predicted to introduce additional amino acids to the *PCSK9* and *Pcsk9* truncated proteins in all cases, the majority of null alleles generated by base editing carried the targeted STOP codons and did not introduce any additional amino acids, thus reducing the possibility of new epitope generation (Fig. 4c and Additional file 9: Table S3, Additional file 10: Table S4, Additional file 11: Table S5).

We recently described VIVO (verification of in vivo off-targets), a highly sensitive approach that can robustly identify the genome-wide off-target effects of CRISPR-Cas nucleases in vivo, and previously used this strategy to investigate off-target effects of Cas9-gMH in the liver from hPCSK9-KI mice [41]. In the first step (in vitro), we identified 529 potential off-target sites by CIRCLE-Seq; in the second step (in vivo), we examined the 69 sites that had the highest CIRCLE-seq read counts (and up to 6 mismatches) together with the on-target site and found significant indel mutations only at the on-target mouse *Pcsk9* locus and the human *PCSK9* transgene [41]. To evaluate potential off-target effects induced by BE-gMH treatment in the current study, we used deep sequencing to analyze sites (among the 69 sites validated in vivo for Cas9-gMH) that harbored at least one cytosine in the BE window (and up to 3 mismatches; chosen based on a recent study showing that several guide RNAs with 2 or 3 mismatches were highly active with BE3 but not with Cas9 or vice versa [41, 42]). We also examined 9 other potential off-target sites predicted by bioinformatics analysis (Additional file 12: Table S6 and Additional file 13: Table S**7**). We did not detect any point mutations or indels at off-target sites either identified by CIRCLE-seq or predicted bioinformatically (Fig. 4d). These results extend findings from a previous study that used bionformatic prediction tools only and did not find any evidence of off-target mutagenesis when using BE3 and gMH to target mouse *Pcsk9* [29].

Chromosomal translocations, which result from double-strand breaks in chromosomes and subsequent joining of loose ends of different chromosome arms, may limit the therapeutic use of genomic editing technologies because of their oncogenic potential [43, 44]. Because we did not observe any detectable off-target effect of gMH for base editing (Fig. 4d), we assumed that any possible translocation event depending on genome editing and base editing treatment would only occur between the mouse *Pcsk9* locus (on-target), which is on the long arm of chromosome 4, and the human *PCSK9*, which is knocked into the *Rosa* 26 locus on the long arm of chromosome 6 and can be considered an artificial off-target. Hence, we looked for the presence of balanced and acentric inter-chromosomal translocations that resulted in the fusion of mouse *Pcsk9* exon 3 with human *PCSK9* exon 3. Again, we used genomic DNA from liver tissue 3 weeks after injecting 10-week-old hPCSK9-KI mice with adenoviral vectors encoding Cas9-gMH and BE-gMH, and performed PCR, using a forward primer annealing to the mouse gene and a reverse primer annealing to the human locus for assessment of a potential balanced translocation and a forward primer annealing to the mouse gene and a forward primer annealing to the human locus for assessment of a potential acentric translocation (Fig. 4e). Of note, we observed bands of the expected size only in samples that were treated with Cas9-gMH but not in samples treated with BE-gMH (Fig. 4f). This result demonstrates the advantage of base editing over genome editing to avoid unwanted genetic rearrangements.

## Discussion

Here, we generated a mouse model exhibiting a human-like hypercholesterolemic phenotype driven by liver-specific expression of human *PCSK9*. Using two approaches, we confirmed that the hypercholesterolemia was driven by expression of the human *PCSK9.* First, cholesterol levels were reduced by treatment with a monoclonal antibody targeting human PCSK9. Second, Cas9-mediated genome editing using guide RNAs that targeted specifically human *PCSK9* or mouse *Pcsk9* showed that inhibition of the human but not the mouse gene was required to achieve an effect on cholesterol levels. We also showed that base editing treatment reduced circulating cholesterol in this mouse model, and subsequently used the model as a tool to compare the mutagenic effects of base editing and Cas9-mediated genome editing. We noted that base editing resulted in a more pronounced reduction in plasma PCSK9 levels relative to the frequency of editing at the genetic level. Greater reductions in plasma PCSK9 levels compared with the frequency of editing have also been reported after Cas9 treatment [22, 24]. One potential explanation for this phenomenon is that the decreased PCSK9 concentration in plasma following genome or base editing leads to an increase in the concentration of LDL receptors at the cell surface. This may occur because PCSK9 normally binds to a LDL receptor with subsequent internalization of both molecules. Thus, as a result of increased LDL receptor availability, there might then be an additional reduction in PCSK9 plasma levels. This relationship would be consistent with previously published data showing that individuals with only one or no WT *LDLR* gene(s) have higher levels of circulating PCSK9 protein [45]. Another potential explanation is that adenoviral vectors mostly infect hepatocytes, but the frequency of on-target alteration is assessed for all cell types in the liver, thereby potentially underestimating the true mutation frequency in hepatocytes. The lower efficiency of base editors compared with Cas9 can be explained by the recent observation that base editor expression is lower than that of Cas9 [46]. The use of a codon-optimized version of a base editor could increase the efficiency of base editing [47].

Base editing is emerging as a new technology that can be as efficient as Cas9-mediated genome editing but is more precise because it can introduce specific point mutations and does not rely on the error-prone NHEJ pathway. Our study confirmed that base editing can be applied in vivo to insert precise point mutations without any detectable guide RNA-dependent off-target mutations at CIRCLE-Seq or bioinformatically predicted sites. In agreement with previous studies [25, 31], we showed that the number of indels was significantly lower following base editing compared with Cas9-mediated genome editing in vivo. We also showed that base editing could potentially generate fewer newly formed peptides, thus reducing the probability of an immune response compared with Cas9-mediated genome editing. Finally, we showed in vivo evidence of chromosomal translocations only after Cas9-mediated genome editing and not base editing. These findings are in line with recently described safety concerns linked to Cas9-induced double-strand DNA cleavage [48], and suggest evaluation of more precise Cas9 variants to reduce the number of translocations in vivo*.* In addition, more unbiased methods for assessing genomic rearrangement events should also be explored. Before translating base editing to the clinic, further in vivo studies are required to assess the presence of potential guide RNA-independent off-targets induced by non-specific deamination due to ApoBecC1 overexpression. However, our results indicate important advantages of base editing.

## Conclusions

We predict that our humanized hPCSK9-KI mouse model described here can be used to evaluate therapeutic strategies spanning from classical pharmaceutical inhibitors (antibodies, antisense, and small molecules) to innovative genetic approaches directed against *PCSK9*, to compare delivery methods and to assess the safety of drug treatment and genome editing in vivo.

## Methods

### Generation of the hPCSK9-KI mouse model

The hPCSK9-KI mouse line was generated by expressing the human *PCSK9* cDNA under the control of a 2.34 kb mouse albumin promoter/enhancer [49]. We constructed a targeting vector for homologous recombination of the mouse *Rosa 26* (*R26*) locus. Two *R26* homology arms of 5.5 kbp 5′ and 1.7 kbp 3′ respectively flank the albumin promoter/enhancer hPCSK9-cDNA fragment (Fig. 1a). To select for positively targeted embryonic stem (ES) cells, a floxed pol II promoter-driven neomycin phosphotransferase (*Neo*) selectable marker cassette was included. The floxed cassette also harbors a cDNA for *Cre* recombinase under the control of a 698 bp testis-specific angiotensin converting enzyme promoter [50]. The target construct was electroporated into C57BL6 (Prx) ES cells. Neo-resistant clones were analyzed for correct integration into the *R26* locus by PCR using the primers listed in Additional file 13: Table S7. Thirteen positive clones were further screened by Southern blot, using a Neo probe. One validated clone was expanded and injected into Balb/cAnNCrl blastocysts to generate chimeric mice. Chimeric C57BL/6N hPCSK9 heterozygous males were bred to C57BL/6N females to generate experimental animals, which are littermates with two genotypes: C57BL/6N hPCSK9KI+/− (referred to as hPCSK9-KI) and WT C57BL/6 N hPCSK9KI−/− (referred to as WT). Black-coated offspring were genotyped for carrying the *R26* Alb*PCSK9* knock-in modification, using the primers listed in Additional file 13: Table S7 P1 (3′ end of human *PCSK9* cDNA), P2 (3′ of insertion in *R26* locus and P3 5′ of insertion).

### Animals

All mouse experiments were approved by the AstraZeneca internal committee for animal studies and the Gothenburg Ethics Committee for Experimental Animals (license no 162–2015+) compliant with EU directives on the protection of animals used for scientific purposes. For all mouse experiments, male hPCSK9-KI mice and their WT littermates were housed individually in a temperature-controlled room (22 °C) with a 12:12-h light/dark cycle. They were fed chow diet (Cat. N. D12450J, Research Diets, New Brunswick, NJ) and water ad libitum.

### Total cholesterol and LDL cholesterol measurements

Plasma lipids from fasted hPCSK9-KI and WT mice were isolated as described previously [51]. Total plasma cholesterol was measured using an enzymatic colorimetric method (Cat. N. A11A01634; ABX Pentra 400; HORIBA Medical, Irvine, CA) [52]. High-density lipoprotein (HDL), LDL, and very-low-density lipoprotein (VLDL) subfractions were separated by anion-exchange high-performance liquid chromatography as described previously [52].

### Mouse and human PCSK9 protein levels

Enzyme-linked immunosorbent assays (ELISA) were performed on mouse plasma (diluted 1:800–1:1000) using the Mouse and Human Proprotein convertase 9 Quantikine ELISA Kit (Cat. N. MPC900 (mouse) and MPC900 (human); R&D Systems, Minneapolis, MN) according to the manufacturer’s protocol.

### Histology

Liver and other tissues from the mice were immediately fixed in 4% neutral buffered formalin, embedded in paraffin and finally prepared in 5 μm thick sections. Liver sections were stained for hematoxylin and eosin (H&E, morphology characterization), mouse PCSK9 (anti-mouse PCSK9 antibody, Dil. 1:500; Cat. N. ab31762; Lot: GR102562-1; RRID:AB_777140; Abcam, Cambridge, UK), human PCSK9 (anti-human PCSK9 antibody, Dil. 1:500; Cat. N. ab28770; Lot: GR90399–1; RRID:AB_777137; Abcam, Cambridge, UK), and LDL receptor (anti-LDL receptor antibody, Dil. 1:100; Cat. N. ab52818, Lot: GR295148-10; RRID:AB_881213; Abcam, Cambridge, UK). Pancreas, skeletal muscle, kidney, white adipose tissue, brown adipose tissue, and spleen sections were stained for human PCSK9 using the antibody described above. All histological slides were blinded and examined using light microscopy (Carl Zeiss Microscopy GmbH, Jena, Germany) by an experienced board-certified pathologist.

### RT-PCR

Total RNA was isolated from mouse liver using the RNeasy Mini Kit (Cat. N. 74106; Qiagen, Valencia, CA). cDNA was generated using the high-capacity cDNA reverse transcription Kit (Cat. N. 4368813; ThermoFisher Scientific, Stockholm, Sweden) and used for quantitative real-time PCR with the QuantStudio 7 Flex (Applied Biosystems, Stockholm, Sweden), using human *PCSK9* and mouse *Pcsk9* gene expression assays (Taqman, Life Technologies Europe, Stockholm, Sweden). The results were normalized to β-actin as a reference gene. Quantitative measures were obtained applying the ΔΔCT method.

### Evolocumab treatment of hPCSK9-KI mice

Ten-week-old male hPCSK9-KI mice were randomized into two experimental groups, and a single subcutaneous injection of saline or evolocumab (Repatha from Amgen Europe, Breda, Holland) 10 mg/kg was administered as described previously [14, 53]. Three days before injection and 1 day after, blood from saphenous vena was collected in EDTA-coated tubes, centrifuged (1500×*g*, 20 min) and plasma was stored at − 80 °C upon analysis. One week after antibody administration, all mice were killed using isoflurane inhalation and exsanguination. Blood from inferior vena cava was collected in EDTA-coated tubes, and plasma was obtained as above.

### Guide RNA design and in vitro cleavage assay

The guide RNA gM (GGCTGATGAGGCCGCACATG) that specifically targets mouse *Pcsk9* has been previously described [22]. We used bioinformatics to design a guide RNA gH that specifically targets the human *PCSK9* locus (GTCCCGCGGGCGCCCGTGCGC) and minimize off-target effects. The guide RNA gMH (CAGGTTCCATGGGATGCTCT) has been previously described [29] and shows perfect alignment to the mouse gene and one nucleotide mismatch (position 10 distal to the protospacer adjacent motif) to the human gene. Cas9-mediated cleavage with all the gRNAs used was evaluated and confirmed by using the Surveyor assay (Cat. N. 706025; Integrated DNA Technologies, BVBA, Leuven, Belgium) according to the manufacturer’s instructions.

### Plasmids and adenoviral constructs

For pcDNA3.1-Cas9 and pcDNA3.1-BE3 plasmid construction, the Cas9 and BE3 inserts were assembled from synthetic oligonucleotides and/or PCR products and cloned into a pcDNA3.1 (+) backbone. For pcDNA3.1-gH and pcDNA3.1-gMH plasmid construction, the gH and gMH guide RNAs were cloned under the U6 promoter into a pcDNA3.1 (+) backbone.

The adenoviral constructs encoding Cas9-GFP, Cas9-gH, Cas9-gM, Cas9-gMH, BE3, and BE3-gMH were generated by Vector Biolabs (Malvern, PA). Gene-specific guide RNAs were cloned under the transcriptional control U6 promoter and codon-optimized BE3 and Cas9 under the transcriptional control of the chicken β-actin hybrid (CBh) promoter or the cytomegalovirus (CMV) promoter, into an adenoviral type 5 (dE1/E3) backbone.

### Cell studies

Human HEK293T cells (ATCC) were cultured in DMEM+GlutaMAX medium (ThermoFisher Scientific) supplemented with 10% FBS and 1% penicillin-streptomycin (ThermoFisher Scientific).

For the Surveyor assay, HEK293T cells were seeded into 12-well plates at 70–80% confluency and transfected with 500 ng of pcDNA3.1-gH or pcDNA3.1-gMH and 500 ng of pcDNA3.1-Cas9 plasmids by using the FuGENE HD transfection reagent (Promega). Seventy-two hours post-transfection, the cells were collected and genomic DNA was isolated using the Gentra Puregene Cell and Tissue kit (Cat. N. 158667; Qiagen, Valencia, CA). The human *PCSK9* locus was amplified using the primers listed in Additional file 13: Table S7. PCR products were digested with the Surveyor mismatch cleavage assay (Integrated DNA Technologies), and mutagenesis was visualized on 10% TBE polyacrylamide gel.

For testing BE3 activity in combination with gMH, HEK293T cells were seeded into 12-well plates at 70–80% confluency a day before transfection with 500 ng of pcDNA3.1-gMH and 500 ng of pCDNA-BE3 by using the FuGENE HD transfection reagent (Promega). Seventy-two hours post-transfection, cells were collected and genomic DNA was isolated using the Gentra Puregene Cell and Tissue kit (Cat. N. 158667; Qiagen, Valencia, CA). The human *PCSK9* locus was amplified using adapter containing gene-specific primers (Additional file 13: Table S7) for deep sequencing.

### CRISPR-Cas9 and base editing treatment of hPCSK9-KI mice

For genome editing, 28-week-old male hPCSK9-KI mice were randomized into 4 groups and injected with adenoviral vectors expressing Cas9-GFP, Cas9-gM or Cas9-gH (1 × 10^9^ adenoviral particles per mouse in 200 μl PBS), or Cas9-gM/gH (0.5 × 10^9^ adenoviral particles of Cas9-gM and 0.5 × 10^9^ adenoviral particles of Cas9-gH in 200 μl PBS) into the tail vein. For base editing, 10-week-old male hPCSK9-KI mice were randomized into 2 groups and injected with adenoviral vectors expressing BE3 or BE3-gMH (1 × 10^9^ adenoviral particles per mouse in 200 μl PBS). To compare genome and base editing directly, 10-week-old male hPCSK9-KI mice were also injected with adenoviral vectors expressing Cas9-gMH (1 × 10^9^ adenoviral particles per mouse in 200 μl PBS) into the tail vein.

Three days before injection, blood from saphenous vena was collected in EDTA-coated tubes, centrifuged (1500×*g*, 20 min) and plasma was stored at − 80 °C before analysis. Three weeks after injection, mice were killed using isoflurane inhalation and exsanguination. Blood from inferior vena cava was collected in EDTA-coated tubes, and plasma isolated as described above. In addition, biopsies from different tissues were collected for morphological and molecular analyses.

### Targeted deep sequencing

Genomic DNA isolated from HEK293T cells or liver tissue from adenovirus-injected mice was amplified with adapter-containing gene-specific primers (Additional file 13: Table S7); linker for forward primers: TCGTCGGCAGCGTCAGATGTGTATAAGAGACAG; linker for reverse primers: GTCTCGTGGGCTCGGAGATGTGTATAAGAGACAG) using Q5 Hot Start High Fidelity DNA polymerase (NEB) and sequenced by using a NextSeq500 Instrument (Illumina). Sequencing reads were demultiplexed using Illumina software and paired FASTQ files were analyzed using CRISPResso^2^. Briefly, reads with a minimum average quality score of 33 were aligned to the reference sequence. A window of 30 base pairs centered on the predicted cleavage site was specified for the quantification of indels and base-editing outcome. The frequencies of in-frame and frameshifting mutations were automatically calculated using CRISPResso^2^. The frequency of mutated alleles was calculated based on the CRISPResso’s detected alleles, “Alleles_frequency_table.txt,” in NGS data as follows: first, all of the detected alleles were imported into the Microsoft Excel program. Next, the number of reads for alleles with identical 30 nucleotides around the predicted cut site was consolidated. Alleles with a frequency of less than 0.01% were excluded from the analysis. Finally, the 10 most frequent alleles among the consolidated alleles were translated to amino acid sequences, and the relative frequency of each allele was calculated.

### Off-target analysis

GUIDE-seq was performed on HEK293T cells to evaluate the extent of potential off-target sites of gH gRNA as described previously [38]. Data were analyzed using AstraZeneca-developed algorithms and software.

For the analysis of BE3-gMH off-target sites in vivo, we selected those that had up to 3 mismatches relative to gMH and harboring at least one cytosine in the BE window (Additional file 12: Table S6) among the sites previously evaluated for Cas9-gMH [41] and examined them by deep sequencing. Data were analyzed and visualized by using AstraZeneca-developed algorithms and software.

### Assessment of translocations

Genomic DNA from liver tissue of adenovirus-injected mice was isolated using the Gentra Puregene Cell and Tissue kit (Cat. N. 158667; Qiagen, Valencia, CA) and amplified with gene specific primers to assess for potentially balanced and acentric translocation events. For assessment of the balanced translocation, genomic DNA was amplified with a forward primer annealing to intron 2–3 of mouse *Pcks9* and a reverse primer annealing to exon 4 of human *PCSK9* (Additional file 13: Table S7). For assessment of the acentric translocation, genomic DNA was amplified with a forward primer annealing to intron 2–3 of mouse Pcks9 and a forward primer annealing to exon 1 of human PCSK9 (Additional file 13: Table S7). Amplicons were analyzed on 10% Novex TBE Gels (ThermoFisher Scientific).

### Statistical analysis

Comparisons between groups were performed using statistical linear models including univariate regression and ANOVA. When relevant, Dunnett’s post hoc test was performed for correction of multiple hypotheses; otherwise, significance is reported for estimated regression coefficients (estimated effects). *p* < 0.05 between the groups was considered to be statistically significant. Data visualization was performed using GraphPad Prism7.02.

## Additional files

Additional file 1: **Figure S1.** Liver-specific expression of human PCSK9 in the hPCSK9-KI mouse model. (**a**) Representative micrographs of liver tissues from a WT (left) and a hPCSK9-KI mouse (right) showing expression of human PCSK9 (hPCSK9, brown) only in the hPCSK9-KI mouse liver. (**b**) Representative micrographs of tissues from a hPCSK9-KI mouse showing no hPCSK9 expression in pancreatic islets, skeletal muscle, kidney, white adipose tissue (WAT), brown adipose tissue (BAT), and spleen. Tissues were incubated with antibodies against hPCSK9. Scale bars, 200 μm. (PDF 3790 kb)

Additional file 2: **Table S1.** Lipoprotein profile of WT and hPCSK9-KI mice. Plasma concentrations (in nanomolar) of HDL-cholesterol (HDL-C), LDL-cholesterol (LDL-C), VLDL-cholesterol (VLDL-C), and total cholesterol in WT and hPCSK9-KI mice at 10 and 28 weeks of age. HDL-C, LDL-C, VLDL-C, and total cholesterol concentrations are presented as group means ± SD; *n* = 4–29; data were analyzed with univariate linear regression, *p* values correspond to *t* tests for estimated regression coefficients (effects) for the comparison between hPCSK9 and WT mice at 10 weeks or 28 weeks of age. *p < 0.05; **p < 0.005; ***p < 0.0005; ****p < 0.0001. (PDF 194 kb)

Additional file 3: **Figure S2.** CRISPR-Cas9 targeting strategy and editing efficiency. (**a**) Schematic of exon 1 of human *PCSK9* (top) and mouse *Pcsk9* (bottom) loci. Nucleotides are depicted as the distance from the ATG (orange box). The guide RNA gH has perfect complementarity to a sequence within exon 1 of human *PSCK9* while gM has eight mismatches to the most similar sequence within that exon, and there is no NGG protospacer adjacent motif (PAM) in the proximity. The guide RNA gM has perfect complementarity to a sequence within exon 1 of mouse *Pcsk9* while gH has six mismatches to the most similar sequence within that exon. Therefore, active cleavage is expected with gH only at human *PCSK9* and with gM only at mouse *Pcsk9* with no cross reactivity*.* (**b**) Surveyor mismatch cleavage assay shows gH cleavage activity in HEK293T cells. Cells were co-transfected with plasmids encoding Cas9 and gH and genomic DNA was analyzed 3 days later. The gel image demonstrates cleaving efficacy of gH at the human *PCSK9* locus. (**c**) Surveyor mismatch cleavage assay on genomic DNA from liver tissue of hPCSK9-KI mice 3 weeks after injection with adenoviral vectors encoding Cas9 together with gH, gM, both gH and gM (gH/gM), or GFP; mice were 28 weeks old at the time of injection. The gel image demonstrates cleaving efficacy of gH at the human *PCSK9* locus, gM at the mouse *Pcsk9* locus, and gM/gH at both loci. (PDF 1479 kb)

Additional file 4: **Table S2.** List of GUIDE-Seq-detected off-target sites for gH. (PDF 274 kb)

Additional file 5: **Figure S3.** Analysis of liver tissue from hPCSK9-KI mice 3 weeks after Cas9 treatment. Twenty-eight-week-old hPCSK9-KI mice were injected with adenoviral vectors encoding Cas9 together with gH, gM, both gH and gM (gH/gM), or GFP. Representative micrographs show staining with hematoxylin and eosin (H&E) and antibodies against human PCSK9 (hPCSK9, brown), mouse Pcks9 (mPCSK9, brown), and LDL receptors (LDL-R, brown). Scale bars, 200 μm. CV, central vein of the liver. (PDF 1391 kb)

Additional file 6: **Figure S4.** Cas9-gH treatment in hPCSK9-KI mice. (**a**) Surveyor mismatch cleavage assay on genomic DNA from liver tissue of hPCSK9-KI mice 3 weeks after injection with adenoviral vectors encoding Cas9 together with GFP (as control) or gH; mice were 10 weeks old at the time of injection. The gel image demonstrates cleaving efficacy of gH at the human *PCSK9* locus. (**b**) Plasma concentrations of human PCSK9 protein after treatment with Cas9-gH or Cas9-GFP in hPCSK9-KI mice (normalized to pretreatment plasma concentrations; *n* = 6 per group). (**c**, **d**) Plasma concentrations of (**c**) mouse PCSK9 protein and (**d**) total cholesterol 3 weeks after treatment with Cas9-gH or Cas9-GFP in WT or hPCSK9-KI mice (normalized to pretreatment plasma concentrations; *n* = 6 per group). Data were analyzed with univariate linear regression; reported *p* values correspond to *t* tests for estimated regression coefficients (effects). *p < 0.05, *n* = 6 per group. Values are presented as group means ± SEM. *p < 0.05; **p < 0.001. (**e**) Representative micrographs of liver tissues from hPCSK9-KI mice 3 weeks after treatment with Cas9-gH or Cas9-GFP. Tissues were stained with hematoxylin and eosin (H&E) for tissue morphology evaluation and antibodies against human PCSK9 (hPCSK9, brown) and LDL receptors (LDL-R, brown). Scale bars, 200 μm. CV, central vein of the liver. (PDF 5244 kb)

Additional file 7: **Figure S5.** Analysis of single nucleotide substitutions at the human *PCSK9* locus. (**a**) Percentage of single base changes at the human *PCSK9* target site in BE3-gMH-treated HEK293T cells. Cells were co-transfected with plasmids encoding BE3 and gMH and genomic DNA was analyzed by deep sequencing after 3 days. gMH targets codon W159 (TGG) within the human *PCSK9* locus; the two targeted G_s_ are in positions 13 and 14 of the protospacer adjacent motif (G_13_ and G_14_, respectively). (**b**) Percentage of single base changes at the human *PCSK9* (left) and mouse *Pcsk9* (right) target sites in the liver from BE3-gMH-treated hPCSK9-KI mice; mice were 10 weeks old at the time of injection, and genomic DNA was analyzed by deep sequencing 3 weeks after treatment. (PDF 236 kb)

Additional file 8: **Figure S6.** Analysis of liver tissue from hPCSK9-KI mice 3 weeks after BE3 treatment. 10-week-old hPCSK9-KI mice were injected with adenoviral vectors encoding BE3 alone or together with gMH. Representative micrographs show staining with hematoxylin and eosin (H&E) and antibodies against human PCSK9 (hPCSK9, brown) and LDL receptors (LDL-R, brown). Scale bars, 200 μm. CV, central vein of the liver. (PDF 1664 kb)

Additional file 9: **Table S3.** Frequency of null alleles generated by BE3-gMH and Cas9-gMH treatment in hPCSK9-KI mice. (PDF 202 kb)

Additional file 10: **Table S4.** Most frequent mutant alleles generated by BE3-gMH and Cas9-gMH treatment in hPCSK9-KI mice. (PDF 678 kb)

Additional file 11: **Table S5.** Frequency of targeted stop codons generated by BE3-gMH. (PDF 179 kb)

Additional file 12: **Table S6.** List of off-target sites for gMH. (PDF 439 kb)

Additional file 13: **Table S7.** List of primers used in this study. (PDF 215 kb)

Additional file 14: Raw data. (XLSX 21 kb)
