# Supplementary figures and images for: In vivo genome and base editing of a human PCSK9 knock-in hypercholesterolemic mouse model
Source: BMC Biol. 2019 Jan 15;17:4. doi: 10.1186/s12915-018-0624-2 (PMC6334452; doi:10.1186/s12915-018-0624-2)

# Additional file 1: Fig. S1

**a**

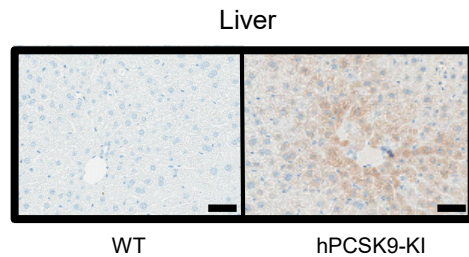

**b**

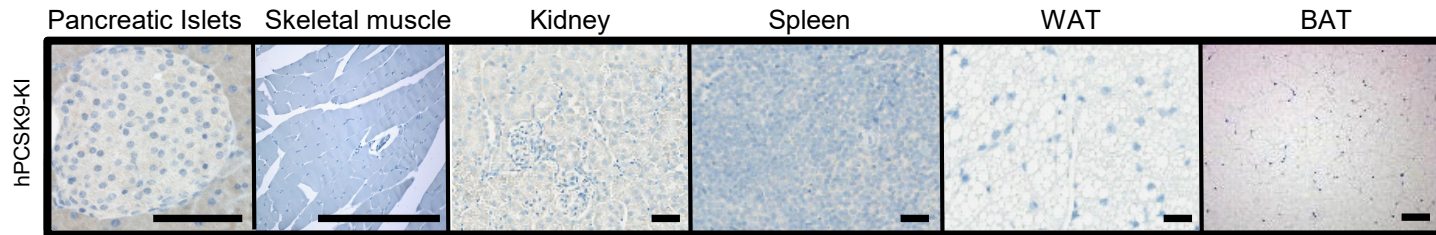

Supplement: Supplementary file 1 — Figure S1. Liver-specific expression of human PCSK9 in the hPCSK9-KI mouse model. (a) Representative micrographs of liver tissues from a WT (left) and a hPCSK9-KI mouse (right) showing expression of human PCSK9 (hPCSK9, brown) only in the hPCSK9-KI mouse liver. (b) Representative micrographs of tissues from a hPCSK9-KI mouse showing no hPCSK9 expression in pancreatic islets, skeletal muscle, kidney, white adipose tissue (WAT), brown adipose tissue (BAT), and spleen. Tissues were incubated with antibodies against hPCSK9. Scale bars, 200 μm. (PDF 3790 kb) [file 12915_2018_624_MOESM1_ESM.pdf]

Additional file 3: Fig. S2

a

Exon 1- human *PCSK9*

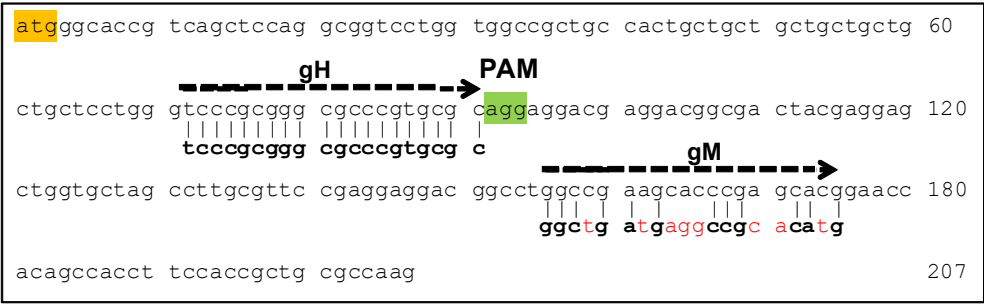

Exon 1-mouse *Pcsk9*

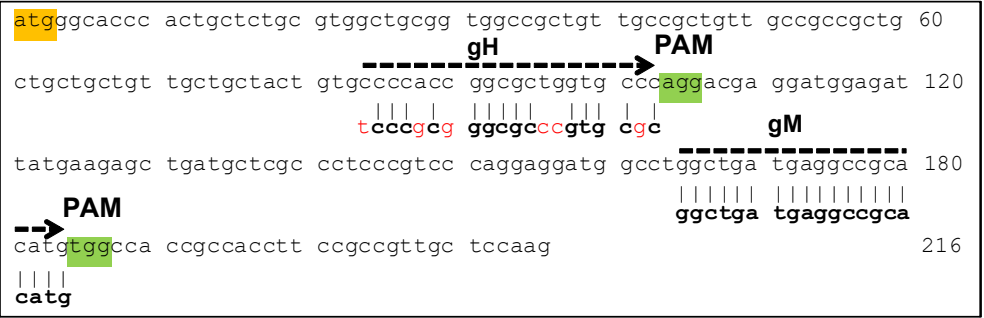

b

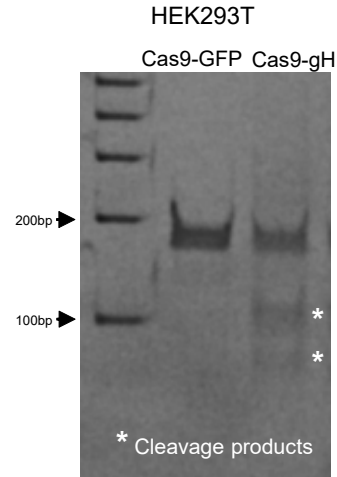

c

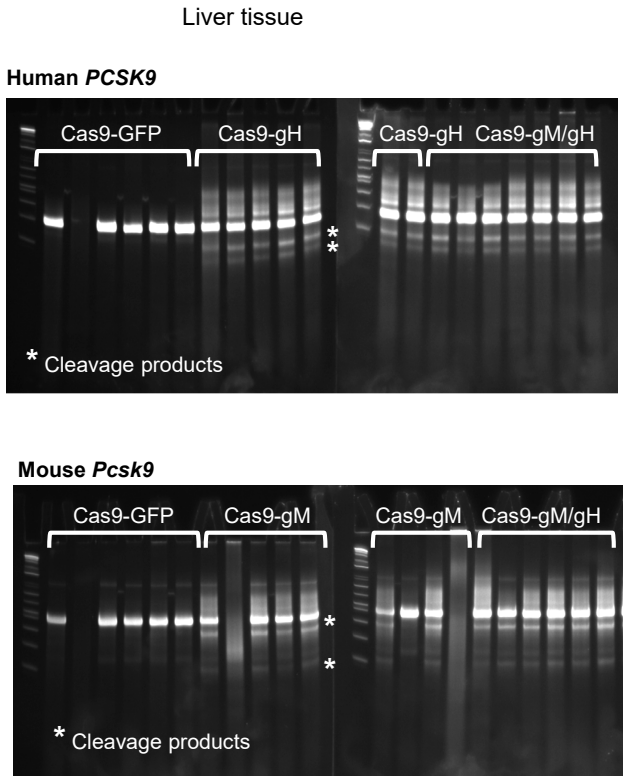

Supplement: Supplementary file 3 — Figure S2. CRISPR-Cas9 targeting strategy and editing efficiency. (a) Schematic of exon 1 of human PCSK9 (top) and mouse Pcsk9 (bottom) loci. Nucleotides are depicted as the distance from the ATG (orange box). The guide RNA gH has perfect complementarity to a sequence within exon 1 of human PSCK9 while gM has eight mismatches to the most similar sequence within that exon, and there is no NGG protospacer adjacent motif (PAM) in the proximity. The guide RNA gM has perfect complementarity to a sequence within exon 1 of mouse Pcsk9 while gH has six mismatches to the most similar sequence within that exon. Therefore, active cleavage is expected with gH only at human PCSK9 and with gM only at mouse Pcsk9 with no cross reactivity. (b) Surveyor mismatch cleavage assay shows gH cleavage activity in HEK293T cells. Cells were co-transfected with plasmids encoding Cas9 and gH and genomic DNA was analyzed 3 days later. The gel image demonstrates cleaving efficacy of gH at the human PCSK9 locus. (c) Surveyor mismatch cleavage assay on genomic DNA from liver tissue of hPCSK9-KI mice 3 weeks after injection with adenoviral vectors encoding Cas9 together with gH, gM, both gH and gM (gH/gM), or GFP; mice were 28 weeks old at the time of injection. The gel image demonstrates cleaving efficacy of gH at the human PCSK9 locus, gM at the mouse Pcsk9 locus, and gM/gH at both loci. (PDF 1479 kb) [file 12915_2018_624_MOESM3_ESM.pdf]

Additional file 5: Fig. S3

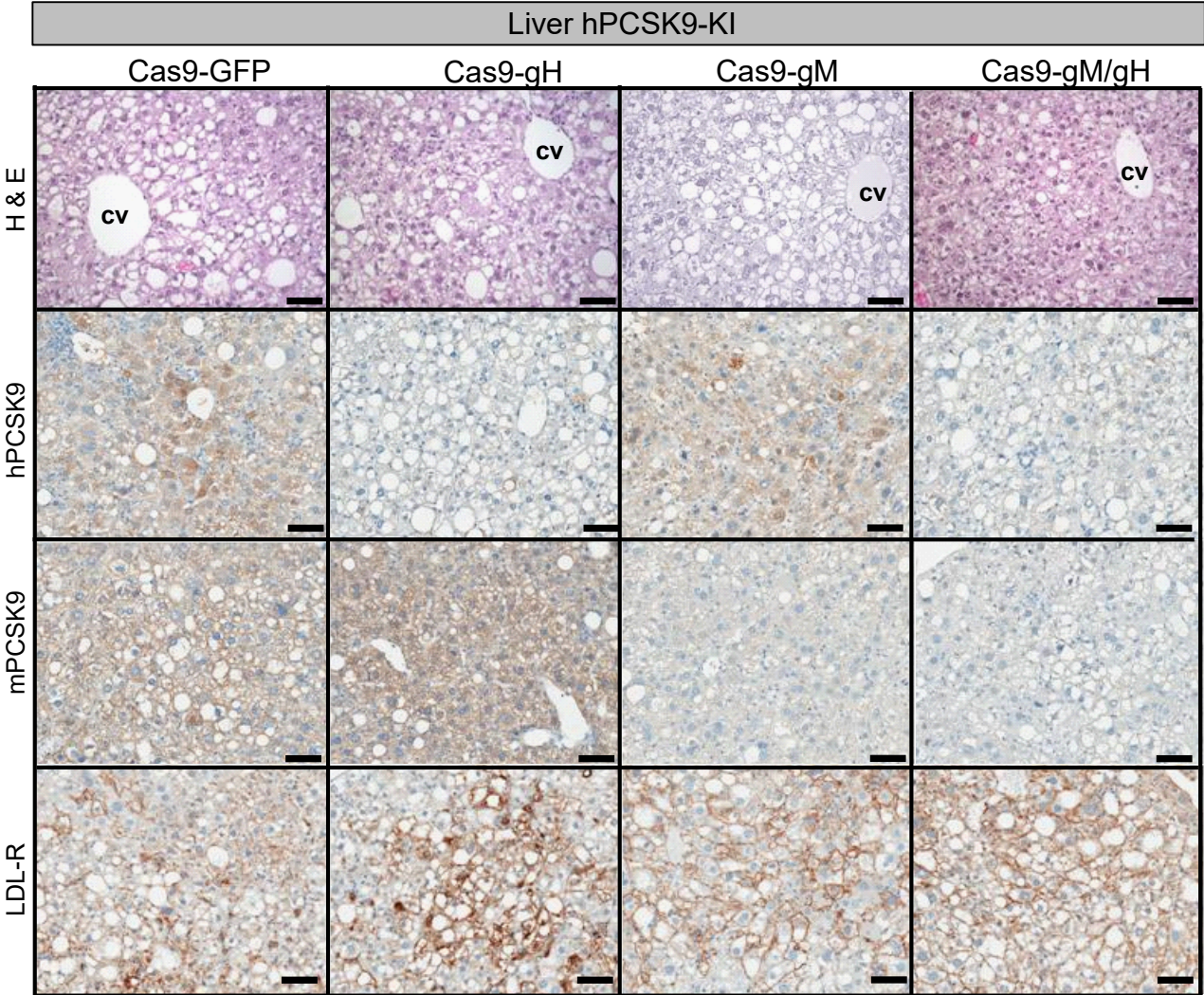

Supplement: Supplementary file 5 — Figure S3. Analysis of liver tissue from hPCSK9-KI mice 3 weeks after Cas9 treatment. Twenty-eight-week-old hPCSK9-KI mice were injected with adenoviral vectors encoding Cas9 together with gH, gM, both gH and gM (gH/gM), or GFP. Representative micrographs show staining with hematoxylin and eosin (H&E) and antibodies against human PCSK9 (hPCSK9, brown), mouse Pcks9 (mPCSK9, brown), and LDL receptors (LDL-R, brown). Scale bars, 200 μm. CV, central vein of the liver. (PDF 1391 kb) [file 12915_2018_624_MOESM5_ESM.pdf]

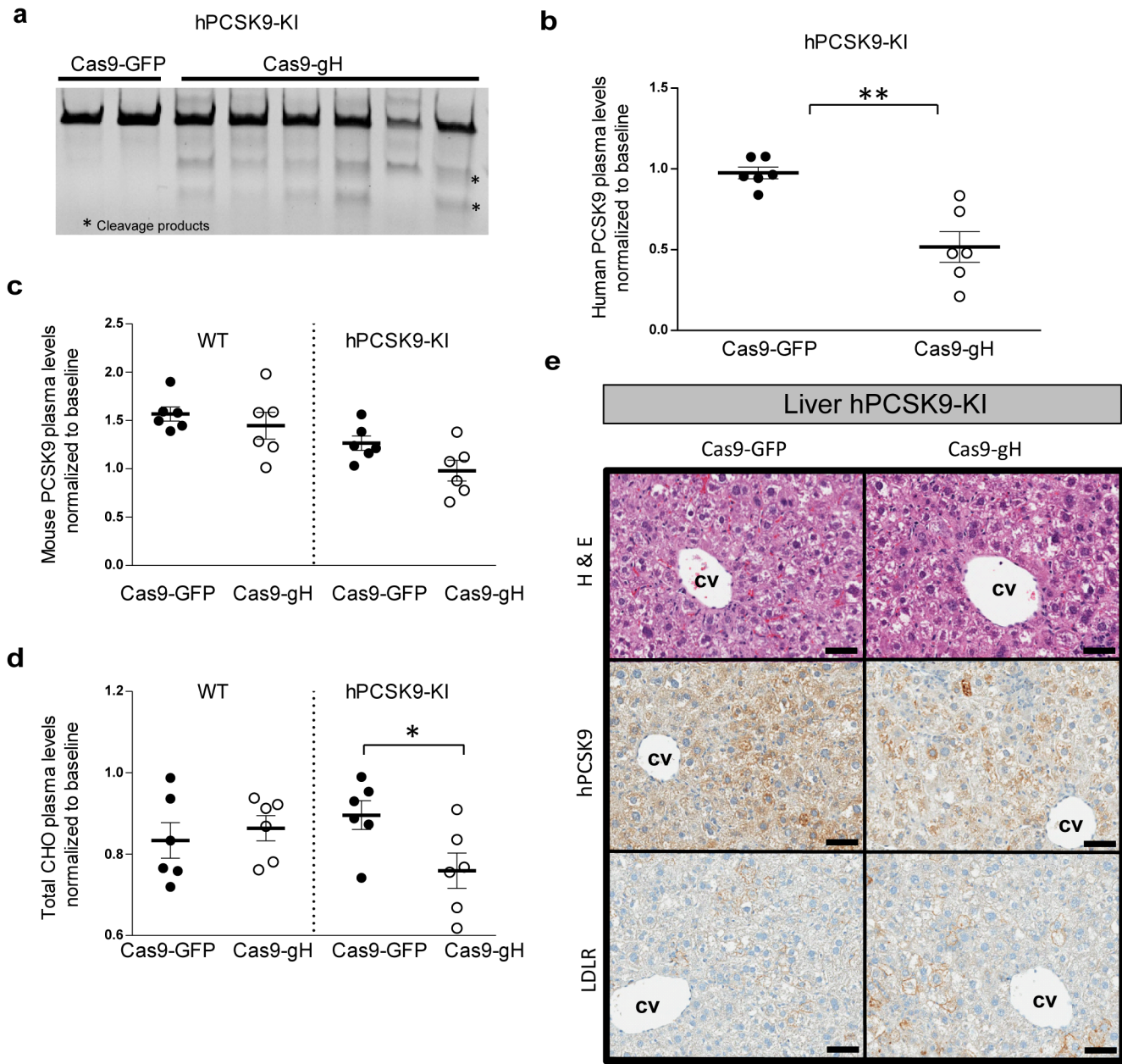

Supplement: Supplementary file 6 — Figure S4. Cas9-gH treatment in hPCSK9-KI mice. (a) Surveyor mismatch cleavage assay on genomic DNA from liver tissue of hPCSK9-KI mice 3 weeks after injection with adenoviral vectors encoding Cas9 together with GFP (as control) or gH; mice were 10 weeks old at the time of injection. The gel image demonstrates cleaving efficacy of gH at the human PCSK9 locus. (b) Plasma concentrations of human PCSK9 protein after treatment with Cas9-gH or Cas9-GFP in hPCSK9-KI mice (normalized to pretreatment plasma concentrations; n = 6 per group). (c, d) Plasma concentrations of (c) mouse PCSK9 protein and (d) total cholesterol 3 weeks after treatment with Cas9-gH or Cas9-GFP in WT or hPCSK9-KI mice (normalized to pretreatment plasma concentrations; n = 6 per group). Data were analyzed with univariate linear regression; reported p values correspond to t tests for estimated regression coefficients (effects). *p < 0.05, n = 6 per group. Values are presented as group means ± SEM. *p < 0.05; **p < 0.001. (e) Representative micrographs of liver tissues from hPCSK9-KI mice 3 weeks after treatment with Cas9-gH or Cas9-GFP. Tissues were stained with hematoxylin and eosin (H&E) for tissue morphology evaluation and antibodies against human PCSK9 (hPCSK9, brown) and LDL receptors (LDL-R, brown). Scale bars, 200 μm. CV, central vein of the liver. (PDF 5244 kb) [file 12915_2018_624_MOESM6_ESM.pdf]

# Additional file 8: Fig. S6

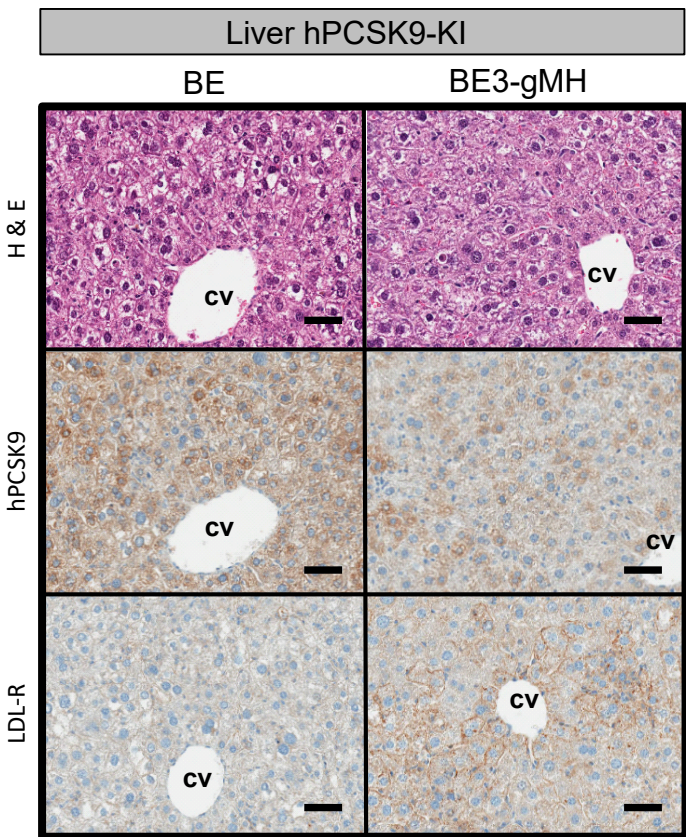

Supplement: Supplementary file 8 — Figure S6. Analysis of liver tissue from hPCSK9-KI mice 3 weeks after BE3 treatment. 10-week-old hPCSK9-KI mice were injected with adenoviral vectors encoding BE3 alone or together with gMH. Representative micrographs show staining with hematoxylin and eosin (H&E) and antibodies against human PCSK9 (hPCSK9, brown) and LDL receptors (LDL-R, brown). Scale bars, 200 μm. CV, central vein of the liver. (PDF 1664 kb) [file 12915_2018_624_MOESM8_ESM.pdf]
