# Supplementary material for: In vivo genome and base editing of a human PCSK9 knock-in hypercholesterolemic mouse model
Source: BMC Biol. 2019 Jan 15;17:4. doi: 10.1186/s12915-018-0624-2 (PMC6334452; doi:10.1186/s12915-018-0624-2)
Supplement: Supplementary file 2 — Table S1. Lipoprotein profile of WT and hPCSK9-KI mice. Plasma concentrations (in nanomolar) of HDL-cholesterol (HDL-C), LDL-cholesterol (LDL-C), VLDL-cholesterol (VLDL-C), and total cholesterol in WT and hPCSK9-KI mice at 10 and 28 weeks of age. HDL-C, LDL-C, VLDL-C, and total cholesterol concentrations are presented as group means ± SD; n = 4–29; data were analyzed with univariate linear regression, p values correspond to t tests for estimated regression coefficients (effects) for the comparison between hPCSK9 and WT mice at 10 weeks or 28 weeks of age. *p < 0.05; **p < 0.005; ***p < 0.0005; ****p < 0.0001. (PDF 194 kb) [file 12915_2018_624_MOESM2_ESM.pdf]

Additional file 2: Table S1

Lipoprotein profile of WT and hPCSK9-KI mice.

	10 weeks		28 weeks	
	WT mice	hPCSK9-KI mice	WT mice	hPCSK9-KI mice
HDL-C	1.23±0.31	1.33±0.10	1.57±0.35	2.12±0.37**
LDL-C	0.68±0.05	1.53±0.13***	2.53±0.71	5.95±1.29****
VLDL-C	0.05±0.02	0.09±0.02*	0.10±0.02	0.16±0.05**
Total cholesterol	1.93±0.39	2.95±0.21**	4.21±1.06	8.22±1.54***
