# Supplementary material for: In vivo genome and base editing of a human PCSK9 knock-in hypercholesterolemic mouse model
Source: BMC Biol. 2019 Jan 15;17:4. doi: 10.1186/s12915-018-0624-2 (PMC6334452; doi:10.1186/s12915-018-0624-2)
Supplement: Supplementary file 4 — Table S2. List of GUIDE-Seq-detected off-target sites for gH. (PDF 274 kb) [file 12915_2018_624_MOESM4_ESM.pdf]

Additional file 4: Table S2

List of GUIDE-Seq-detected off-targets for gH.

PCSK9 target site																				GUIDE-Seq reads	On and off-target sites
T	C	C	C	G	C	G	G	G	C	G	C	C	C	G	T	G	C	G	C		PCSK9
.	.	.	.	.	.	.	.	.	.	.	.	.	.	.	.	.	.	.	.	2265	PCSK9
C	.	.	.	.	.	A	.	.	.	.	.	.	.	.	A	.	.	.	.	624	Non-coding
G	.	.	.	.	.	.	.	.	.	.	.	.	.	.	G	.	.	.	.	229	TGFB1I1 intron
