# Supplementary material for: In vivo genome and base editing of a human PCSK9 knock-in hypercholesterolemic mouse model
Source: BMC Biol. 2019 Jan 15;17:4. doi: 10.1186/s12915-018-0624-2 (PMC6334452; doi:10.1186/s12915-018-0624-2)

Additional file 7: Fig. S5

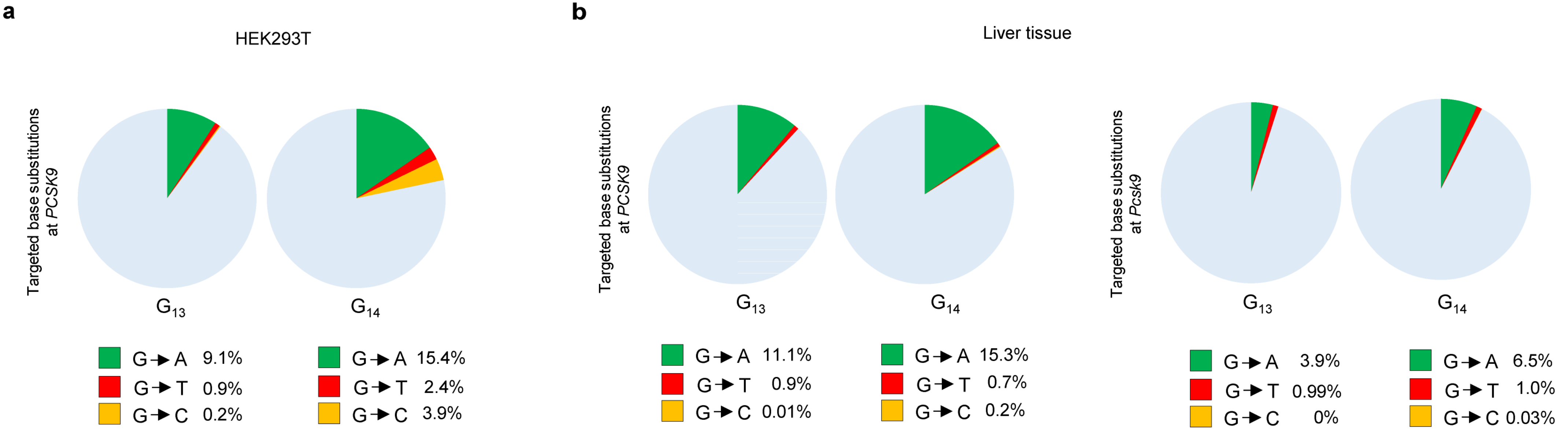

Supplement: Supplementary file 7 — Figure S5. Analysis of single nucleotide substitutions at the human PCSK9 locus. (a) Percentage of single base changes at the human PCSK9 target site in BE3-gMH-treated HEK293T cells. Cells were co-transfected with plasmids encoding BE3 and gMH and genomic DNA was analyzed by deep sequencing after 3 days. gMH targets codon W159 (TGG) within the human PCSK9 locus; the two targeted Gs are in positions 13 and 14 of the protospacer adjacent motif (G13 and G14, respectively). (b) Percentage of single base changes at the human PCSK9 (left) and mouse Pcsk9 (right) target sites in the liver from BE3-gMH-treated hPCSK9-KI mice; mice were 10 weeks old at the time of injection, and genomic DNA was analyzed by deep sequencing 3 weeks after treatment. (PDF 236 kb) [file 12915_2018_624_MOESM7_ESM.pdf]
