# Supplementary material for: In vivo genome and base editing of a human PCSK9 knock-in hypercholesterolemic mouse model
Source: BMC Biol. 2019 Jan 15;17:4. doi: 10.1186/s12915-018-0624-2 (PMC6334452; doi:10.1186/s12915-018-0624-2)
Supplement: Supplementary file 9 — Table S3. Frequency of null alleles generated by BE3-gMH and Cas9-gMH treatment in hPCSK9-KI mice. (PDF 202 kb) [file 12915_2018_624_MOESM9_ESM.pdf]

Additional file 9: Table S3

Frequency of null alleles generated by BE3-gMH and Cas9-gMH treatment in hPCSK9-KI mice.

Treatment	Locus	Mouse ID	Editing (absolute %)	Nonsense mutations (absolute %)	In-frame/frameshift mutations (absolute %)	Total null alleles (absolute %)	Total null alleles (relative %)
BE3-gMH	*Pcsk9*	#1	5,53	2,90	0,24	3,14	56,78
BE3-gMH	*Pcsk9*	#2	6,74	3,94	0,25	4,19	62,17
BE3-gMH	*Pcsk9*	#3	11,60	7,66	0,54	8,20	70,69
BE3-gMH	*Pcsk9*	#4	10,30	6,37	0,65	7,02	68,16
BE3-gMH	*Pcsk9*	#5	10,30	4,60	1,56	6,16	59,81
BE3-gMH	*Pcsk9*	#6	11,10	7,38	1,72	9,10	81,98
BE3-gMH	*Pcsk9*	#7	14,40	11,35	2,18	13,53	93,96
BE3-gMH	*Pcsk9*	#8	11,90	6,12	1,44	7,56	63,53
		Average	***10,23***	***6,29***	***1,07***	***7,36***	***69,63***

BE3-gMH	*PCSK9*	#1	11,11	7,38	0,86	8,24	74,17
BE3-gMH	*PCSK9*	#2	14,41	9,92	1,70	11,62	80,64
BE3-gMH	*PCSK9*	#3	21,11	14,50	2,52	17,02	80,63
BE3-gMH	*PCSK9*	#4	24,08	17,66	2,20	19,86	82,48
BE3-gMH	*PCSK9*	#5	25,13	10,53	1,38	11,91	47,39
BE3-gMH	*PCSK9*	#6	25,05	16,10	2,12	18,22	72,73
BE3-gMH	*PCSK9*	#7	34,93	24,52	2,80	27,32	78,21
BE3-gMH	*PCSK9*	#8	28,29	17,07	2,26	19,33	68,33
		Average	***23,01***	***14,71***	***1,98***	***16,69***	***73,07***

Cas9-gMH	*Pcsk9*	#1	29,10	0,07	19,79	19,86	68,25
Cas9-gMH	*Pcsk9*	#2	25,43	0,06	17,86	17,92	70,47
Cas9-gMH	*Pcsk9*	#3	13,70	0,71	6,68	7,39	53,94
Cas9-gMH	*Pcsk9*	#4	16,48	0,14	10,76	10,90	66,14
Cas9-gMH	*Pcsk9*	#5	38,16	0,28	27,09	27,37	71,72
Cas9-gMH	*Pcsk9*	#6	25,12	0,63	15,71	16,34	65,05
		Average	***24,43***	***2,37***	***14,27***	***16,64***	***65,93***

Cas9-gMH	*PCSK9*	#1	31,08	0,07	25,59	25,66	82,56
Cas9-gMH	*PCSK9*	#2	26,05	0,07	21,47	21,54	82,69
Cas9-gMH	*PCSK9*	#3	10,23	0,33	5,35	5,68	55,52
Cas9-gMH	*PCSK9*	#4	15,42	0,53	8,93	9,46	61,35
Cas9-gMH	*PCSK9*	#5	31,45	0,35	24,17	24,52	77,97
Cas9-gMH	*PCSK9*	#6	19,33	0,22	14,40	14,62	75,63
		Average	***22,57***	***0,56***	***16,31***	***16,87***	***72,62***
