# Supplementary material for: In vivo genome and base editing of a human PCSK9 knock-in hypercholesterolemic mouse model
Source: BMC Biol. 2019 Jan 15;17:4. doi: 10.1186/s12915-018-0624-2 (PMC6334452; doi:10.1186/s12915-018-0624-2)
Supplement: Supplementary file 10 — Table S4. Most frequent mutant alleles generated by BE3-gMH and Cas9-gMH treatment in hPCSK9-KI mice. (PDF 678 kb) [file 12915_2018_624_MOESM10_ESM.pdf]

Additional file 10: Table S4

Most frequent mutant alleles generated by BE3-gMH and Cas9-gMH treatment in hPCSK9-KI mice.

Alleles	#Reads	Protein Sequence	Type	Outcome	Percentage
ATTGAGGAAGACTCCTTTGTCTTCGCCCAGAGCATCCCATGGAACCTGGAGCGAATTATCC	639 466	Ile Glu Glu Asp Ser Phe Val Phe Ala Gln Ser Ile Pro Trp Asn Leu Glu Arg Ile Ile	Wild type	Wild type	70,895
ATTGAGGAAGACTCCTTTGTCTTCGCCCAG-GCATCCCATGGAACCTGGAGCGAATTATCC	30 886	Ile Glu Glu Asp Ser Phe Val Phe Ala Gln Ala Ser His Gly Thr Trp Ser Glu Leu Ser	Frameshift deletion	Frameshift Mutation	3,424
ATTGAGGAAGACTCCTTTGTCTTCGCCCAGA-CATCCCATGGAACCTGGAGCGAATTATCC	28 789	Ile Glu Glu Asp Ser Phe Val Phe Ala Gln Thr Ser His Gly Thr Trp Ser Glu Leu Ser	Frameshift deletion	Frameshift Mutation	3,192
ATTGAGGAAGACTCCTTTGTCTTCGCCCAGA**A**GCATCCCATGGAACCTGGAGCGAATTATCC	18 394	Ile Glu Glu Asp Ser Phe Val Phe Ala Gln Lys His Pro Met Glu Pro Gly Ala Asn Tyr	Frameshift insertion	Frameshift Mutation	2,039
ATTGAGGAAGACTCCTTTGTCTTCGCCCAGAG**G**CATCCCATGGAACCTGGAGCGAATTATCC	13 238	Ile Glu Glu Asp Ser Phe Val Phe Ala Gln Arg His Pro Met Glu Pro Gly Ala Asn Tyr	Frameshift insertion	Frameshift Mutation	1,468
ATTGAGGAAGACTCCTTTGTCTTCGCCCAGA**T**GCATCCCATGGAACCTGGAGCGAATTATCC	9 361	Ile Glu Glu Asp Ser Phe Val Phe Ala Gln Met His Pro Met Glu Pro Gly Ala Asn Tyr	Frameshift insertion	Frameshift Mutation	1,038
ATTGAGGAAGACTCCTTTGTCTTCGCCC--AGCATCCCATGGAACCTGGAGCGAATTATCC	9 260	Ile Glu Glu Asp Ser Phe Val Phe Ala Gln His Pro Met Glu Pro Gly Ala Asn Tyr	Frameshift deletion	Frameshift Mutation	1,027
ATTGAGGAAGACTCCTTTGTCTTCGCCCA------------GAACCTGGAGCGAATTATCC	4 404	Ile Glu Glu Asp Ser Phe Val Phe Ala Gln Asn Leu Glu Arg Ile Ile	Inframe deletion	Inframe Mutation	0,488

Cas9-gMH (#2_*mPcsk9*)

Alleles	#Reads	Protein Sequence	Type	Outcome	Percentage
ATTGAGGAAGACTCCTTTGTCTTCGCCCAGAGCATCCCATGGAACCTGGAGCGAATTATCC	405 587	Ile Glu Glu Asp Ser Phe Val Phe Ala Gln Ser Ile Pro Trp Asn Leu Glu Arg Ile Ile	Wild type	Wild type	74,565
ATTGAGGAAGACTCCTTTGTCTTCGCCCAGA-CATCCCATGGAACCTGGAGCGAATTATCC	16 414	Ile Glu Glu Asp Ser Phe Val Phe Ala Gln Thr Ser His Gly Thr Trp Ser Glu Leu Ser	Frameshift deletion	Frameshift Mutation	3,018
ATTGAGGAAGACTCCTTTGTCTTCGCCCAG-GCATCCCATGGAACCTGGAGCGAATTATCC	14 968	Ile Glu Glu Asp Ser Phe Val Phe Ala Gln Ala Ser His Gly Thr Trp Ser Glu Leu Ser	Frameshift deletion	Frameshift Mutation	2,752
ATTGAGGAAGACTCCTTTGTCTTCGCCCAGA**A**GCATCCCATGGAACCTGGAGCGAATTATCC	7 914	Ile Glu Glu Asp Ser Phe Val Phe Ala Gln Lys His Pro Met Glu Pro Gly Ala Asn Tyr	Frameshift insertion	Frameshift Mutation	1,455
ATTGAGGAAGACTCCTTTGTCTTCGCCCAGAG**G**CATCCCATGGAACCTGGAGCGAATTATCC	7 115	Ile Glu Glu Asp Ser Phe Val Phe Ala Gln Arg His Pro Met Glu Pro Gly Ala Asn Tyr	Frameshift insertion	Frameshift Mutation	1,308
ATTGAGGAAGACTCCTTTGTCTTCGCCC--AGCATCCCATGGAACCTGGAGCGAATTATCC	7 088	Ile Glu Glu Asp Ser Phe Val Phe Ala Gln His Pro Met Glu Pro Gly Ala Asn Tyr	Frameshift deletion	Frameshift Mutation	1,303
ATTGAGGAAGACTCCTTTGTCTTCGCCCAGA**T**GCATCCCATGGAACCTGGAGCGAATTATCC	4 151	Ile Glu Glu Asp Ser Phe Val Phe Ala Gln Met His Pro Met Glu Pro Gly Ala Asn Tyr	Frameshift insertion	Frameshift Mutation	0,763
ATTGAGGAAGACTCCTTTGTCTTCGCCCAG---ATCCCATGGAACCTGGAGCGAATTATCC	2 263	Ile Glu Glu Asp Ser Phe Val Phe Ala Gln Ile Pro Trp Asn Leu Glu Arg Ile Ile	Inframe deletion	Inframe Mutation	0,416

Cas9-gMH (#3_*mPcsk9*)

Alleles	#Reads	Protein Sequence	Type	Outcome	Percentage
GAGGAAGACTCCTTTGTCTTCGCCCAGAGCATCCCATGGAACCTGGAGCGAATTATCC	241 998	Glu Glu Asp Ser Phe Val Phe Ala Gln Ser Ile Pro Trp Asn Leu Glu Arg Ile Ile	Wild type	Wild type	86,277
GAGGAAGACTCCTTTGTCTTCGCCCAGA-CATCCCATGGAACCTGGAGCGAATTATCC	2 771	Glu Glu Asp Ser Phe Val Phe Ala Gln Thr Ser His Gly Thr Trp Ser Glu Leu Ser	Frameshift deletion	Frameshift Mutation	0,988
GAGGAAGACTCCTTTGTCTTCGCCCAG-GCATCCCATGGAACCTGGAGCGAATTATCC	2 533	Glu Glu Asp Ser Phe Val Phe Ala Gln Ala Ser His Gly Thr Trp Ser Glu Leu Ser	Frameshift deletion	Frameshift Mutation	0,903
GAGGAAGACTCCTTTGTCTTCGCCCAGA**A**GCATCCCATGGAACCTGGAGCGAATTATCC	1 658	Glu Glu Asp Ser Phe Val Phe Ala Gln Lys His Pro Met Glu Pro Gly Ala Asn Tyr	Frameshift insertion	Frameshift Mutation	0,591
GAGGAAGACTCCTTTGTCTTCGCCC--AGCATCCCATGGAACCTGGAGCGAATTATCC	1 199	Glu Glu Asp Ser Phe Val Phe Ala Gln His Pro Met Glu Pro Gly Ala Asn Tyr	Frameshift deletion	Frameshift Mutation	0,427
GAGGAAGACTCCTTTGTCTTCGCCCAGAGCATCC**A**ATGGAACCTGGAGCGAATTATCC	1 092	Glu Glu Asp Ser Phe Val Phe Ala Gln Ser Ile Gln Trp Asn Leu Glu Arg Ile Ile	Substitution, Pro>Gln	Missense Mutation	0,389
GAGGAAGACTCCTTTGTCTTCGCC**A**AGAGCATCCCATGGAACCTGGAGCGAATTATCC	1 075	Glu Glu Asp Ser Phe Val Phe Ala Lys Ser Ile Pro Trp Asn Leu Glu Arg Ile Ile	Substitution, Gln>Lys	Missense Mutation	0,383
GAGGAAGACTCCTTTGTCTTCGCCCAGAG**G**CATCCCATGGAACCTGGAGCGAATTATCC	1 071	Glu Glu Asp Ser Phe Val Phe Ala Gln Arg His Pro Met Glu Pro Gly Ala Asn Tyr	Frameshift insertion	Frameshift Mutation	0,382
GAGGAAGACTCCTTTGTCTTCGCCCAGAGCATCCCATGGAACCTG**T**AGCGAATTATCC	874	Glu Glu Asp Ser Phe Val Phe Ala Gln Ser Ile Pro Trp Asn Leu **Stp**	Substitution, Glu>Stp	Nonsense Mutation, Knockout	0,312

Cas9-gMH (#4_*mPcsk9*)

Alleles	#Reads	Protein Sequence	Type	Outcome	Percentage
GAGGAAGACTCCTTTGTCTTCGCCCAGAGCATCCCATGGAACCTGGAGCGAATTATCC	352 427	Glu Glu Asp Ser Phe Val Phe Ala Gln Ser Ile Pro Trp Asn Leu Glu Arg Ile Ile	Wild type	Wild type	83,519
GAGGAAGACTCCTTTGTCTTCGCCCAGA-CATCCCATGGAACCTGGAGCGAATTATCC	8 972	Glu Glu Asp Ser Phe Val Phe Ala Gln Thr Ser His Gly Thr Trp Ser Glu Leu Ser	Frameshift deletion	Frameshift Mutation	2,126
GAGGAAGACTCCTTTGTCTTCGCCCAG-GCATCCCATGGAACCTGGAGCGAATTATCC	7 546	Glu Glu Asp Ser Phe Val Phe Ala Gln Ala Ser His Gly Thr Trp Ser Glu Leu Ser	Frameshift deletion	Frameshift Mutation	1,788
GAGGAAGACTCCTTTGTCTTCGCCCAGA**A**GCATCCCATGGAACCTGGAGCGAATTATCC	4 135	Glu Glu Asp Ser Phe Val Phe Ala Gln Lys His Pro Met Glu Pro Gly Ala Asn Tyr	Frameshift insertion	Frameshift Mutation	0,980
GAGGAAGACTCCTTTGTCTTCGCCC--AGCATCCCATGGAACCTGGAGCGAATTATCC	2 889	Glu Glu Asp Ser Phe Val Phe Ala Gln His Pro Met Glu Pro Gly Ala Asn Tyr	Frameshift deletion	Frameshift Mutation	0,685
GAGGAAGACTCCTTTGTCTTCGCCCAGAG**G**CATCCCATGGAACCTGGAGCGAATTATCC	2 056	Glu Glu Asp Ser Phe Val Phe Ala Gln Arg His Pro Met Glu Pro Gly Ala Asn Tyr	Frameshift insertion	Frameshift Mutation	0,487
GAGGAAGACTCCTTTGTCTTCGCCCAGA**T**GCATCCCATGGAACCTGGAGCGAATTATCC	1 510	Glu Glu Asp Ser Phe Val Phe Ala Gln Met His Pro Met Glu Pro Gly Ala Asn Tyr	Frameshift insertion	Frameshift Mutation	0,358
GAGGAAGACTCCTTTGTCTTCGCCCAG---ATCCCATGGAACCTGGAGCGAATTATCC	979	Glu Glu Asp Ser Phe Val Phe Ala Gln Ile Pro Trp Asn Leu Glu Arg Ile Ile	Inframe deletion	Inframe Mutation	0,232
GAGGAAGACTCCTTTGTCTTCG----------CCCATGGAACCTGGAGCGAATTATCC	796	Glu Glu Asp Ser Phe Val Phe Ala His Gly Thr Trp Ser Glu Leu Ser	Frameshift deletion	Frameshift Mutation	0,189
GAGGAAGACTCCTTTGTCTTCGCCCAGA--ATCCCATGGAACCTGGAGCGAATTATCC	642	Glu Glu Asp Ser Phe Val Phe Ala Gln Asn Pro Met Glu Pro Gly Ala Asn Tyr	Frameshift deletion	Frameshift Mutation	0,152

Cas9-gMH (#5_*mPcsk9*)

Alleles	#Reads	Protein Sequence	Type	Outcome	Percentage
GAGGAAGACTCCTTTGTCTTCGCCCAGAGCATCCCATGGAACCTGGAGCGAATTATCC	141 381	Glu Glu Asp Ser Phe Val Phe Ala Gln Ser Ile Pro Trp Asn Leu Glu Arg Ile Ile	Wild type	Wild type	61,838
GAGGAAGACTCCTTTGTCTTCGCCCAGA-CATCCCATGGAACCTGGAGCGAATTATCC	13 811	Glu Glu Asp Ser Phe Val Phe Ala Gln Thr Ser His Gly Thr Trp Ser Glu Leu Ser	Frameshift deletion	Frameshift Mutation	6,041
GAGGAAGACTCCTTTGTCTTCGCCCAG-GCATCCCATGGAACCTGGAGCGAATTATCC	10 038	Glu Glu Asp Ser Phe Val Phe Ala Gln Ala Ser His Gly Thr Trp Ser Glu Leu Ser	Frameshift deletion	Frameshift Mutation	4,390
GAGGAAGACTCCTTTGTCTTCGCCCAGAAGCATCCCATGGAACCTGGAGCGAATTATCC	5 480	Glu Glu Asp Ser Phe Val Phe Ala Gln Lys His Pro Met Glu Pro Gly Ala Asn Tyr	Frameshift insertion	Frameshift Mutation	2,397
GAGGAAGACTCCTTTGTCTTCGCCC--AGCATCCCATGGAACCTGGAGCGAATTATCC	4 496	Glu Glu Asp Ser Phe Val Phe Ala Gln His Pro Met Glu Pro Gly Ala Asn Tyr	Frameshift deletion	Frameshift Mutation	1,966
GAGGAAGACTCCTTTGTCTTCGCCCAGAGGCATCCCATGGAACCTGGAGCGAATTATCC	3 242	Glu Glu Asp Ser Phe Val Phe Ala Gln Arg His Pro Met Glu Pro Gly Ala Asn Tyr	Frameshift insertion	Frameshift Mutation	1,418
GAGGAAGACTCCTTTGTCTTCGCCCAGATGCATCCCATGGAACCTGGAGCGAATTATCC	2 346	Glu Glu Asp Ser Phe Val Phe Ala Gln Met His Pro Met Glu Pro Gly Ala Asn Tyr	Frameshift insertion	Frameshift Mutation	1,026
GAGGAAGACTCCTTTGTCTTCGCCCAG---ATCCCATGGAACCTGGAGCGAATTATCC	1 231	Glu Glu Asp Ser Phe Val Phe Ala Gln Ile Pro Trp Asn Leu Glu Arg Ile Ile	Inframe deletion	Inframe Mutation	0,538
GAGGAAGACTCCTTTGTCTTCG----------CCCATGGAACCTGGAGCGAATTATCC	1 042	Glu Glu Asp Ser Phe Val Phe Ala His Gly Thr Trp Ser Glu Leu Ser	Frameshift deletion	Frameshift Mutation	0,456
GAGGAAGACTCCTTTGTCTTCGCCCAGA--ATCCCATGGAACCTGGAGCGAATTATCC	906	Glu Glu Asp Ser Phe Val Phe Ala Gln Asn Pro Met Glu Pro Gly Ala Asn Tyr	Frameshift deletion	Frameshift Mutation	0,396

Cas9-gMH (#6_*mPcsk9*)

Alleles	#Reads	Protein Sequence	Type	Outcome	Percentage
GAGGAAGACTCCTTTGTCTTCGCCCAGAGCATCCCATGGAACCTGGAGCGAATTATCC	158 653	Glu Glu Asp Ser Phe Val Phe Ala Gln Ser Ile Pro Trp Asn Leu Glu Arg Ile Ile	Wild type	Wild type	74,878
GAGGAAGACTCCTTTGTCTTCGCCCAGA-CATCCCATGGAACCTGGAGCGAATTATCC	6 436	Glu Glu Asp Ser Phe Val Phe Ala Gln Thr Ser His Gly Thr Trp Ser Glu Leu Ser	Frameshift deletion	Frameshift Mutation	3,038
GAGGAAGACTCCTTTGTCTTCGCCCAG-GCATCCCATGGAACCTGGAGCGAATTATCC	6 042	Glu Glu Asp Ser Phe Val Phe Ala Gln Ala Ser His Gly Thr Trp Ser Glu Leu Ser	Frameshift deletion	Frameshift Mutation	2,852
GAGGAAGACTCCTTTGTCTTCGCCCAGAAGCATCCCATGGAACCTGGAGCGAATTATCC	2 594	Glu Glu Asp Ser Phe Val Phe Ala Gln Lys His Pro Met Glu Pro Gly Ala Asn Tyr	Frameshift insertion	Frameshift Mutation	1,224
GAGGAAGACTCCTTTGTCTTCGCCC--AGCATCCCATGGAACCTGGAGCGAATTATCC	2 290	Glu Glu Asp Ser Phe Val Phe Ala Gln His Pro Met Glu Pro Gly Ala Asn Tyr	Frameshift deletion	Frameshift Mutation	1,081
GAGGAAGACTCCTTTGTCTTCGCCCAGAGGCATCCCATGGAACCTGGAGCGAATTATCC	1 703	Glu Glu Asp Ser Phe Val Phe Ala Gln Arg His Pro Met Glu Pro Gly Ala Asn Tyr	Frameshift insertion	Frameshift Mutation	0,804
GAGGAAGACTCCTTTGTCTTCGCCCAGATGCATCCCATGGAACCTGGAGCGAATTATCC	851	Glu Glu Asp Ser Phe Val Phe Ala Gln Met His Pro Met Glu Pro Gly Ala Asn Tyr	Frameshift insertion	Frameshift Mutation	0,402
GAGGAAGACTCCTTTGTCTTCGCCCAGAGCATCCAATGGAACCTGGAGCGAATTATCC	801	Glu Glu Asp Ser Phe Val Phe Ala Gln Ser Ile Gln Trp Asn Leu Glu Arg Ile Ile	Substitution, Pro>Gln	Missense Mutation	0,378
GAGGAAGACTCCTTTGTCTTCGCCAAGAGCATCCCATGGAACCTGGAGCGAATTATCC	693	Glu Glu Asp Ser Phe Val Phe Ala Lys Ser Ile Pro Trp Asn Leu Glu Arg Ile Ile	Substitution, Gln>Lys	Missense Mutation	0,327
GAGGAAGACTCCTTTGTCTTCGCCCAGA--ATCCCATGGAACCTGGAGCGAATTATCC	683	Glu Glu Asp Ser Phe Val Phe Ala Gln Asn Pro Met Glu Pro Gly Ala Asn Tyr	Frameshift deletion	Frameshift Mutation	0,322

Cas9-gMH (#1_ *hPCSK9*)

Alleles	#Reads	Protein Sequence	Type	Outcome	Percentage
TACATCGAGGAGGACTCCTCTGTCTTTGCCCAGAGCATCCCGTGGAACCTGGAGCGGATTACCCCTC	154 977	Tyr Ile Glu Glu Asp Ser Ser Val Phe Ala Gln Ser Ile Pro Trp Asn Leu Glu Arg Ile Thr Pro	Wild type	Wild type	68,920
TACATCGAGGAGGACTCCTCTGTCTTTGCCCAG-GCATCCCGTGGAACCTGGAGCGGATTACCCCTC	15 725	Tyr Ile Glu Glu Asp Ser Ser Val Phe Ala Gln Ala Ser Arg Gly Thr Trp Ser Gly Leu Pro Leu	Frameshift deletion	Frameshift Mutation	6,993
TACATCGAGGAGGACTCCTCTGTCTTTGCCCAGA-CATCCCGTGGAACCTGGAGCGGATTACCCCTC	10 529	Tyr Ile Glu Glu Asp Ser Ser Val Phe Ala Gln Thr Ser Arg Gly Thr Trp Ser Gly Leu Pro Leu	Frameshift deletion	Frameshift Mutation	4,682
TACATCGAGGAGGACTCCTCTGTCTTTGCCC--AGCATCCCGTGGAACCTGGAGCGGATTACCCCTC	5 519	Tyr Ile Glu Glu Asp Ser Ser Val Phe Ala Gln His Pro Val Glu Pro Gly Ala Asp Tyr Pro	Frameshift deletion	Frameshift Mutation	2,454
TACATCGAGGAGGACTCCTCTGTCTTTGCCCAGAAGCATCCCGTGGAACCTGGAGCGGATTACCCCTC	5 062	Tyr Ile Glu Glu Asp Ser Ser Val Phe Ala Gln Lys His Pro Val Glu Pro Gly Ala Asp Tyr Pro	Frameshift insertion	Frameshift Mutation	2,251
TACATCGAGGAGGACTCCTCTGTCTTTGCCCAGAGGCATCCCGTGGAACCTGGAGCGGATTACCCCTC	2 467	Tyr Ile Glu Glu Asp Ser Ser Val Phe Ala Gln Arg His Pro Val Glu Pro Gly Ala Asp Tyr Pro	Frameshift insertion	Frameshift Mutation	1,097
TACATCGAGGAGGACTCCTCTGTCTTTGCCCAGATGCATCCCGTGGAACCTGGAGCGGATTACCCCTC	1812	Tyr Ile Glu Glu Asp Ser Ser Val Phe Ala Gln Met His Pro Val Glu Pro Gly Ala Asp Tyr Pro	Frameshift insertion	Frameshift Mutation	0,806
TACATCGAGGAGGACTCCTCTGTCTTTGCCCAGA--ATCCCGTGGAACCTGGAGCGGATTACCCCTC	1 038	Tyr Ile Glu Glu Asp Ser Ser Val Phe Ala Gln Asn Pro Val Glu Pro Gly Ala Asp Tyr Pro	Frameshift deletion	Frameshift Mutation	0,462
TACATCGAGGAGGACTCCTCTGTCTTT-------GCATCCCGTGGAACCTGGAGCGGATTACCCCTC	981	Tyr Ile Glu Glu Asp Ser Ser Val Phe Ala Ser Arg Gly Thr Trp Ser Gly Leu Pro Leu	Frameshift deletion	Frameshift Mutation	0,436
TACATCGAGGAGGACTCCTCTGTCTT--------GCATCCCGTGGAACCTGGAGCGGATTACCCCTC	885	Tyr Ile Glu Glu Asp Ser Ser Val Leu His Pro Val Glu Pro Gly Ala Asp Tyr Pro	Frameshift deletion	Frameshift Mutation	0,394

Cas9-gMH (#2_ *hPCSK9*)

Alleles	#Reads	Protein Sequence	Type	Outcome	Percentage
TACATCGAGGAGGACTCCTCTGTCTTTGCCCAGAGCATCCCGTGGAACCTGGAGCGGATTACCCCTC	275 055	Tyr Ile Glu Glu Asp Ser Ser Val Phe Ala Gln Ser Ile Pro Trp Asn Leu Glu Arg Ile Thr Pro	Wild type	Wild type	73,946
TACATCGAGGAGGACTCCTCTGTCTTTGCCCAG-GCATCCCGTGGAACCTGGAGCGGATTACCCCTC	21 750	Tyr Ile Glu Glu Asp Ser Ser Val Phe Ala Gln Ala Ser Arg Gly Thr Trp Ser Gly Leu Pro Leu	Frameshift deletion	Frameshift Mutation	5,847
TACATCGAGGAGGACTCCTCTGTCTTTGCCCAGA-CATCCCGTGGAACCTGGAGCGGATTACCCCTC	17 804	Tyr Ile Glu Glu Asp Ser Ser Val Phe Ala Gln Thr Ser Arg Gly Thr Trp Ser Gly Leu Pro Leu	Frameshift deletion	Frameshift Mutation	4,786
TACATCGAGGAGGACTCCTCTGTCTTTGCCCAGAAGCATCCCGTGGAACCTGGAGCGGATTACCCCTC	8 315	Tyr Ile Glu Glu Asp Ser Ser Val Phe Ala Gln Lys His Pro Val Glu Pro Gly Ala Asp Tyr Pro	Frameshift insertion	Frameshift Mutation	2,235
TACATCGAGGAGGACTCCTCTGTCTTTGCCC--AGCATCCCGTGGAACCTGGAGCGGATTACCCCTC	5 972	Tyr Ile Glu Glu Asp Ser Ser Val Phe Ala Gln His Pro Val Glu Pro Gly Ala Asp Tyr Pro	Frameshift deletion	Frameshift Mutation	1,606
TACATCGAGGAGGACTCCTCTGTCTTTGCCCAGATGCATCCCGTGGAACCTGGAGCGGATTACCCCTC	2 587	Tyr Ile Glu Glu Asp Ser Ser Val Phe Ala Gln Met His Pro Val Glu Pro Gly Ala Asp Tyr Pro	Frameshift insertion	Frameshift Mutation	0,695
TACATCGAGGAGGACTCCTCTGTCTTTGCCCAGAGGCATCCCGTGGAACCTGGAGCGGATTACCCCTC	2241	Tyr Ile Glu Glu Asp Ser Ser Val Phe Ala Gln Arg His Pro Val Glu Pro Gly Ala Asp Tyr Pro	Frameshift insertion	Frameshift Mutation	0,602
----------------------------------GCATCCCGTGGAACCTGGAGCGGATTACCCCTC	1 806	Ala Ser Arg Gly Thr Trp Ser Gly Leu Pro Leu	Frameshift deletion	Frameshift Mutation	0,486
TACATCGAGGAGGACTCCTCTGTCTTTGCCCAG---ATCCCGTGGAACCTGGAGCGGATTACCCCTC	1 587	Tyr Ile Glu Glu Asp Ser Ser Val Phe Ala Gln Ile Pro Trp Asn Leu Glu Arg Ile Thr Pro	Inframe deletion	Inframe Mutation	0,427
TACATCGAGGAGGACTCCTCTGT-----------GCATCCCGTGGAACCTGGAGCGGATTACCCCTC	1 344	Tyr Ile Glu Glu Asp Ser Ser Val His Pro Val Glu Pro Gly Ala Asp Tyr Pro	Frameshift deletion	Frameshift Mutation	0,361

Cas9-gMH (#3_ *hPCSK9*)

Alleles	#Reads	Protein Sequence	Type	Outcome	Percentage
TACATCGAGGAGGACTCCTCTGTCTTTGCCCAGAGCATCCCGTGGAACCTGGAGCGGATTACCCCTC	263 766	Tyr Ile Glu Glu Asp Ser Ser Val Phe Ala Gln Ser Ile Pro Trp Asn Leu Glu Arg Ile Thr Pro	Wild type	Wild type	89,767
TACATCGAGGAGGACTCCTCTGTCTTTGCCCAG-GCATCCCGTGGAACCTGGAGCGGATTACCCCTC	4 852	Tyr Ile Glu Glu Asp Ser Ser Val Phe Ala Gln Ala Ser Arg Gly Thr Trp Ser Gly Leu Pro Leu	Frameshift deletion	Frameshift Mutation	1,651
TACATCGAGGAGGACTCCTCTGTCTTTGCCCAGA-CATCCCGTGGAACCTGGAGCGGATTACCCCTC	3 136	Tyr Ile Glu Glu Asp Ser Ser Val Phe Ala Gln Thr Ser Arg Gly Thr Trp Ser Gly Leu Pro Leu	Frameshift deletion	Frameshift Mutation	1,067
TACATCGAGGAGGACTCCTCTGTCTTTGCCC--AGCATCCCGTGGAACCTGGAGCGGATTACCCCTC	1 643	Tyr Ile Glu Glu Asp Ser Ser Val Phe Ala Gln His Pro Val Glu Pro Gly Ala Asp Tyr Pro	Frameshift deletion	Frameshift Mutation	0,559
TACATCGAGGAGGACTCCTCTGTCTTTGCCCAGAAGCATCCCGTGGAACCTGGAGCGGATTACCCCTC	1418	Tyr Ile Glu Glu Asp Ser Ser Val Phe Ala Gln Lys His Pro Val Glu Pro Gly Ala Asp Tyr Pro	Frameshift insertion	Frameshift Mutation	0,483
TACATCGAGGAGGACTCCTCTGTCTTTGCCCAGAGCATCCCGTGGAACCTGGAGCGGATTACCACTC	672	Tyr Ile Glu Glu Asp Ser Ser Val Phe Ala Gln Ser Ile Pro Trp Asn Leu Glu Arg Ile Thr Thr	Substitution, Pro>Thr	Missense Mutation	0,229
TACATCGAGGAGGACTCCTCTGTCTTTGCCCAGAGCATCCAGTGGAACCTGGAGCGGATTACCCCTC	651	Tyr Ile Glu Glu Asp Ser Ser Val Phe Ala Gln Ser Ile Gln Trp Asn Leu Glu Arg Ile Thr Pro	Substitution, Pro>Gln	Missense Mutation	0,222
TACATCGAGGAGGACTCCTCTGTCTTTGCCCAGAGCATCCCGTGGAACCTGGAGCGGATTACCCATC	598	Tyr Ile Glu Glu Asp Ser Ser Val Phe Ala Gln Ser Ile Pro Trp Asn Leu Glu Arg Ile Thr His	Substitution, Pro>His	Missense Mutation	0,204
TACATCGAGGAGGACTCCTCTGTCTTTGCCAAGAGCATCCCGTGGAACCTGGAGCGGATTACCCCTC	494	Tyr Ile Glu Glu Asp Ser Ser Val Phe Ala Lys Ser Ile Pro Trp Asn Leu Glu Arg Ile Thr Pro	Substitution, Gln>Lys	Missense Mutation	0,168
TACATCGAGGAGGACTCCTCTGTCTTTGCCCAGAGGCATCCCGTGGAACCTGGAGCGGATTACCCCTC	486	Tyr Ile Glu Glu Asp Ser Ser Val Phe Ala Gln Arg His Pro Val Glu Pro Gly Ala Asp Tyr Pro	Frameshift insertion	Frameshift Mutation	0,165

Cas9-gMH (#4_ *hPCSK9*)

Alleles	#Reads	Protein Sequence	Type	Outcome	Percentage
TACATCGAGGAGGACTCCTCTGTCTTTGCCCAGAGCATCCCGTGGAACCTGGAGCGGATTACCCCTC	288 062	Tyr Ile Glu Glu Asp Ser Ser Val Phe Ala Gln Ser Ile Pro Trp Asn Leu Glu Arg Ile Thr Pro	Wild type	Wild type	84,576
TACATCGAGGAGGACTCCTCTGTCTTTGCCCAG-GCATCCCGTGGAACCTGGAGCGGATTACCCCTC	9 185	Tyr Ile Glu Glu Asp Ser Ser Val Phe Ala Gln Ala Ser Arg Gly Thr Trp Ser Gly Leu Pro Leu	Frameshift deletion	Frameshift Mutation	2,697
TACATCGAGGAGGACTCCTCTGTCTTTGCCCAGA-CATCCCGTGGAACCTGGAGCGGATTACCCCTC	6 894	Tyr Ile Glu Glu Asp Ser Ser Val Phe Ala Gln Thr Ser Arg Gly Thr Trp Ser Gly Leu Pro Leu	Frameshift deletion	Frameshift Mutation	2,024
TACATCGAGGAGGACTCCTCTGTCTTTGCCCAGAAGCATCCCGTGGAACCTGGAGCGGATTACCCCTC	2 485	Tyr Ile Glu Glu Asp Ser Ser Val Phe Ala Gln Lys His Pro Val Glu Pro Gly Ala Asp Tyr Pro	Frameshift insertion	Frameshift Mutation	0,730
TACATCGAGGAGGACTCCTCTGTCTTTGCCC--AGCATCCCGTGGAACCTGGAGCGGATTACCCCTC	2407	Tyr Ile Glu Glu Asp Ser Ser Val Phe Ala Gln His Pro Val Glu Pro Gly Ala Asp Tyr Pro	Frameshift deletion	Frameshift Mutation	0,707
TACATCGAGGAGGACTCCTCTGTCTTTGCCCAGAGCATCCCGTGGAACCTGGAGCGGATTACCACTC	1 040	Tyr Ile Glu Glu Asp Ser Ser Val Phe Ala Gln Ser Ile Pro Trp Asn Leu Glu Arg Ile Thr Thr	Substitution, Pro>Thr	Missense Mutation	0,305
TACATCGAGGAGGACTCCTCTGTCTTTGCCCAGAGCATCCAGTGGAACCTGGAGCGGATTACCCCTC	937	Tyr Ile Glu Glu Asp Ser Ser Val Phe Ala Gln Ser Ile Gln Trp Asn Leu Glu Arg Ile Thr Pro	Substitution, Pro>Gln	Missense Mutation	0,275
TACATCGAGGAGGACTCCTCTGTCTTTGCCCAGAGCATCCCGTGGAACCTGGAGCGGATTACCCATC	873	Tyr Ile Glu Glu Asp Ser Ser Val Phe Ala Gln Ser Ile Pro Trp Asn Leu Glu Arg Ile Thr His	Substitution, Pro>His	Missense Mutation	0,256
TACATCGAGGAGGACTCCTCTGTCTTTGCCCAGATGCATCCCGTGGAACCTGGAGCGGATTACCCCTC	852	Tyr Ile Glu Glu Asp Ser Ser Val Phe Ala Gln Met His Pro Val Glu Pro Gly Ala Asp Tyr Pro	Frameshift insertion	Frameshift Mutation	0,250
TACATCGAGGAGGACTCCTCTGTCTTTGCCAAGAGCATCCCGTGGAACCTGGAGCGGATTACCCCTC	816	Tyr Ile Glu Glu Asp Ser Ser Val Phe Ala Lys Ser Ile Pro Trp Asn Leu Glu Arg Ile Thr Pro	Substitution, Gln>Lys	Missense Mutation	0,240

Cas9-gMH (#5_*hPCSK9*)

Alleles	#Reads	Protein Sequence	Type	Outcome	Percentage
TACATCGAGGAGGACTCCTCTGTCTTTGCCCAGAGCATCCCGTGGAACCTGGAGCGGATTACCCCTC	227 333	Tyr Ile Glu Glu Asp Ser Ser Val Phe Ala Gln Ser Ile Pro Trp Asn Leu Glu Arg Ile Thr Pro	Wild type	Wild type	68,548
TACATCGAGGAGGACTCCTCTGTCTTTGCCCAG-GCATCCCGTGGAACCTGGAGCGGATTACCCCTC	22 352	Tyr Ile Glu Glu Asp Ser Ser Val Phe Ala Gln Ala Ser Arg Gly Thr Trp Ser Gly Leu Pro Leu	Frameshift deletion	Frameshift Mutation	6,740
TACATCGAGGAGGACTCCTCTGTCTTTGCCCAGA-CATCCCGTGGAACCTGGAGCGGATTACCCCTC	17 805	Tyr Ile Glu Glu Asp Ser Ser Val Phe Ala Gln Thr Ser Arg Gly Thr Trp Ser Gly Leu Pro Leu	Frameshift deletion	Frameshift Mutation	5,369
TACATCGAGGAGGACTCCTCTGTCTTTGCCC--AGCATCCCGTGGAACCTGGAGCGGATTACCCCTC	7 095	Tyr Ile Glu Glu Asp Ser Ser Val Phe Ala Gln His Pro Val Glu Pro Gly Ala Asp Tyr Pro	Frameshift deletion	Frameshift Mutation	2,139
TACATCGAGGAGGACTCCTCTGTCTTTGCCCAGAAGCATCCCGTGGAACCTGGAGCGGATTACCCCTC	6796	Tyr Ile Glu Glu Asp Ser Ser Val Phe Ala Gln Lys His Pro Val Glu Pro Gly Ala Asp Tyr Pro	Frameshift insertion	Frameshift Mutation	2,049
TACATCGAGGAGGACTCCTCTGTCTTTGCCCAGAGGCATCCCGTGGAACCTGGAGCGGATTACCCCTC	2 100	Tyr Ile Glu Glu Asp Ser Ser Val Phe Ala Gln Arg His Pro Val Glu Pro Gly Ala Asp Tyr Pro	Frameshift insertion	Frameshift Mutation	0,633
TACATCGAGGAGGACTCCTCTGTCTTTGCCCAGATGCATCCCGTGGAACCTGGAGCGGATTACCCCTC	1 775	Tyr Ile Glu Glu Asp Ser Ser Val Phe Ala Gln Met His Pro Val Glu Pro Gly Ala Asp Tyr Pro	Frameshift insertion	Frameshift Mutation	0,535
TACATCGAGGAGGACTCCTCTGT-----------GCATCCCGTGGAACCTGGAGCGGATTACCCCTC	923	Tyr Ile Glu Glu Asp Ser Ser Val His Pro Val Glu Pro Gly Ala Asp Tyr Pro	Frameshift deletion	Frameshift Mutation	0,278
TACATCGAGGAGGACTCCTCTGTCTTTGCCCAG---ATCCCGTGGAACCTGGAGCGGATTACCCCTC	795	Tyr Ile Glu Glu Asp Ser Ser Val Phe Ala Gln Ile Pro Trp Asn Leu Glu Arg Ile Thr Pro	Inframe deletion	Inframe Mutation	0,240
TACATCGAGGAGGACTCCTCTGTCTTTGCCCAGAGCATCCCGTGGAACCTGGAGCGGATTACCACTC	734	Tyr Ile Glu Glu Asp Ser Ser Val Phe Ala Gln Ser Ile Pro Trp Asn Leu Glu Arg Ile Thr Thr	Substitution, Pro>Thr	Missense Mutation	0,221

Cas9-gMH (#6_*hPCSK9*)

Alleles	#Reads	Protein Sequence	Type	Outcome	Percentage
TACATCGAGGAGGACTCCTCTGTCTTTGCCCAGAGCATCCCGTGGAACCTGGAGCGGATTACCCCTC	313 218	Tyr Ile Glu Glu Asp Ser Ser Val Phe Ala Gln Ser Ile Pro Trp Asn Leu Glu Arg Ile Thr Pro	Wild type	Wild type	80,670
TACATCGAGGAGGACTCCTCTGTCTTTGCCCAG-GCATCCCGTGGAACCTGGAGCGGATTACCCCTC	17 531	Tyr Ile Glu Glu Asp Ser Ser Val Phe Ala Gln Ala Ser Arg Gly Thr Trp Ser Gly Leu Pro Leu	Frameshift deletion	Frameshift Mutation	4,515
TACATCGAGGAGGACTCCTCTGTCTTTGCCCAGA-CATCCCGTGGAACCTGGAGCGGATTACCCCTC	14 899	Tyr Ile Glu Glu Asp Ser Ser Val Phe Ala Gln Thr Ser Arg Gly Thr Trp Ser Gly Leu Pro Leu	Frameshift deletion	Frameshift Mutation	3,837
TACATCGAGGAGGACTCCTCTGTCTTTGCCCAGAAGCATCCCGTGGAACCTGGAGCGGATTACCCCTC	5 471	Tyr Ile Glu Glu Asp Ser Ser Val Phe Ala Gln Lys His Pro Val Glu Pro Gly Ala Asp Tyr Pro	Frameshift insertion	Frameshift Mutation	1,409
TACATCGAGGAGGACTCCTCTGTCTTTGCCC--AGCATCCCGTGGAACCTGGAGCGGATTACCCCTC	2828	Tyr Ile Glu Glu Asp Ser Ser Val Phe Ala Gln His Pro Val Glu Pro Gly Ala Asp Tyr Pro	Frameshift deletion	Frameshift Mutation	0,728
TACATCGAGGAGGACTCCTCTGTCTTTGCCCAGAGGCATCCCGTGGAACCTGGAGCGGATTACCCCTC	1 024	Tyr Ile Glu Glu Asp Ser Ser Val Phe Ala Gln Arg His Pro Val Glu Pro Gly Ala Asp Tyr Pro	Frameshift insertion	Frameshift Mutation	0,264
TACATCGAGGAGGACTCCTCTGTCTTTGCCCAGATGCATCCCGTGGAACCTGGAGCGGATTACCCCTC	906	Tyr Ile Glu Glu Asp Ser Ser Val Phe Ala Gln Met His Pro Val Glu Pro Gly Ala Asp Tyr Pro	Frameshift insertion	Frameshift Mutation	0,233
TACATCGAGGAGGACT---CCTC	780	Tyr Ile Glu Glu Asp Ser	Frameshift deletion	Frameshift Mutation	0,201
TACATCGAGGAGGACTCCTCTGTCTTTGCCCAGAGCATCCCGTGGAACCTGGAGCGGATTACCACTC	673	Tyr Ile Glu Glu Asp Ser Ser Val Phe Ala Gln Ser Ile Pro Trp Asn Leu Glu Arg Ile Thr Thr	Substitution, Pro>Thr	Missense Mutation	0,173
----------------------------------GCATCCCGTGGAACCTGGAGCGGATTACCCCTC	619	Ala Ser Arg Gly Thr Trp Ser Gly Leu Pro Leu	Frameshift deletion	Frameshift Mutation	0,159

BE3-gMH (#1_*mPcsk9*)

Alleles	#Reads	Protein Sequence	Type	Outcome	Percentage
ATTGAGGAAGACTCCTTTGTCTTCGCCCAGAGCATCCCATGGAACCTGGAGCGAATTATCC	666 611	Ile Glu Glu Asp Ser Phe Val Phe Ala Gln Ser Ile Pro Trp Asn Leu Glu Arg Ile Ile	Wild type	Wild type	94,466
ATTGAGGAAGACTCCTTTGTCTTCGCCCAGAGCATCCCAT**AA**AACCTGGAGCGAATTATCC	10 600	Ile Glu Glu Asp Ser Phe Val Phe Ala Gln Ser Ile Pro **Stp**	Substitution, Trp>Stp	Nonsense Mutation, Knockout	1,502
ATTGAGGAAGACTCCTTTGTCTTCGCCCAGAGCATCCCATG**A**AACCTGGAGCGAATTATCC	7 676	Ile Glu Glu Asp Ser Phe Val Phe Ala Gln Ser Ile Pro **Stp**	Substitution, Trp>Stp	Nonsense Mutation, Knockout	1,088
ATTGAGGAAGACTCCTTTGTCTTCGCCCAGAGCATCCCATG**T**AACCTGGAGCGAATTATCC	1 377	Ile Glu Glu Asp Ser Phe Val Phe Ala Gln Ser Ile Pro **Cys** Asn Leu Glu Arg Ile Ile	Substitution, Trp>Cys	Missense Mutation	0,195
ATTGAGGAAGACTCCTTTGTCTTCGCCCAGAGCATCCCAT**TA**AACCTGGAGCGAATTATCC	1 349	Ile Glu Glu Asp Ser Phe Val Phe Ala Gln Ser Ile Pro **Leu** Asn Leu Glu Arg Ile Ile	Substitution, Trp>Leu	Missense Mutation	0,191
ATTGAGGAAGACTCCTTTGTCTTCGCCCAGAGCATCCCATG**C**AACCTGGAGCGAATTATCC	666	Ile Glu Glu Asp Ser Phe Val Phe Ala Gln Ser Ile Pro **Cys** Asn Leu Glu Arg Ile Ile	Substitution, Trp>Cys	Missense Mutation	0,094
ATTGAGGAAGACTCCTTTGTCTTCGCCCAGAGCATCCCAT**AC**AACCTGGAGCGAATTATCC	482	Ile Glu Glu Asp Ser Phe Val Phe Ala Gln Ser Ile Pro **Tyr** Asn Leu Glu Arg Ile Ile	Substitution, Trp>Tyr	Missense Mutation	0,068
ATTGAGGAAGACTCCTTTGTCTTCGCCCAGAGCATCCCAT**A**GAACCTGGAGCGAATTATCC	444	Ile Glu Glu Asp Ser Phe Val Phe Ala Gln Ser Ile Pro **Stp**	Substitution, Trp>Stp	Nonsense Mutation, Knockout	0,063
ATTGAGGAAGACTCCTTTGTCTTC**A**CCCAGAGCATCCCATGGAACCTGGAGCGAATTATCC	436	Ile Glu Glu Asp Ser Phe Val Phe **Thr** Gln Ser Ile Pro Trp Asn Leu Glu Arg Ile Ile	Substitution, Ala>Thr	Missense Mutation	0,062
ATTGAGGAAGACTCCTTTGTCTTCGCCCAGA**A**CATCCCATGGAACCTGGAGCGAATTATCC	286	Ile Glu Glu Asp Ser Phe Val Phe Ala Gln **Asn** Ile Pro Trp Asn Leu Glu Arg Ile Ile	Substitution, Ser>Asn	Missense Mutation	0,041

BE3-gMH (#2_*mPcsk9*)

Alleles	#Reads	Protein Sequence	Type	Outcome	Percentage
ATTGAGGAAGACTCCTTTGTCTTCGCCCAGAGCATCCCATGGAACCTGGAGCGAATTATCC	598 065	Ile Glu Glu Asp Ser Phe Val Phe Ala Gln Ser Ile Pro Trp Asn Leu Glu Arg Ile Ile	Wild type	Wild type	93,258
ATTGAGGAAGACTCCTTTGTCTTCGCCCAGAGCATCCCAT**AA**AACCTGGAGCGAATTATCC	14 243	Ile Glu Glu Asp Ser Phe Val Phe Ala Gln Ser Ile Pro **Stp**	Substitution, Trp>Stp	Nonsense Mutation, Knockout	2,221
ATTGAGGAAGACTCCTTTGTCTTCGCCCAGAGCATCCCATG**A**AACCTGGAGCGAATTATCC	9 307	Ile Glu Glu Asp Ser Phe Val Phe Ala Gln Ser Ile Pro **Stp**	Substitution, Trp>Stp	Nonsense Mutation, Knockout	1,451
ATTGAGGAAGACTCCTTTGTCTTCGCCCAGAGCATCCCAT**TA**AACCTGGAGCGAATTATCC	1 897	Ile Glu Glu Asp Ser Phe Val Phe Ala Gln Ser Ile Pro **Leu** Asn Leu Glu Arg Ile Ile	Substitution, Trp>Leu	Missense Mutation	0,296
ATTGAGGAAGACTCCTTTGTCTTCGCCCAGAGCATCCCATG**T**AACCTGGAGCGAATTATCC	955	Ile Glu Glu Asp Ser Phe Val Phe Ala Gln Ser Ile Pro **Cys** Asn Leu Glu Arg Ile Ile	Substitution, Trp>Cys	Missense Mutation	0,149
ATTGAGGAAGACTCCTTTGTCTTCGCCCAGAGCATCCCATG**C**AACCTGGAGCGAATTATCC	763	Ile Glu Glu Asp Ser Phe Val Phe Ala Gln Ser Ile Pro **Cys** Asn Leu Glu Arg Ile Ile	Substitution, Trp>Cys	Missense Mutation	0,119
ATTGAGGAAGACTCCTTTGTCTTCGCCCAGAGCATCCCAT**AC**AACCTGGAGCGAATTATCC	691	Ile Glu Glu Asp Ser Phe Val Phe Ala Gln Ser Ile Pro **Tyr** Asn Leu Glu Arg Ile Ile	Substitution, Trp>Tyr	Missense Mutation	0,108
ATTGAGGAAGACTCCTTTGTCTTCGCCCAGAGCATCCCAT**AA**AACCTG**A**AGCGAATTATCC	300	Ile Glu Glu Asp Ser Phe Val Phe Ala Gln Ser Ile Pro **Stp**	Substitution, Trp>Stp	Nonsense Mutation, Knockout	0,047
ATTGAGGAAGACTCCTTTGTCTTC**A**CCCAGAGCATCCCATGGAACCTGGAGCGAATTATCC	288	Ile Glu Glu Asp Ser Phe Val Phe **Thr** Gln Ser Ile Pro Trp Asn Leu Glu Arg Ile Ile	Substitution, Ala>Thr	Missense Mutation	0,045
ATTGAGGAAGACTCCTTTGTCTTCGCCCAGA**A**CATCCCATGGAACCTGGAGCGAATTATCC	243	Ile Glu Glu Asp Ser Phe Val Phe Ala Gln **Asn** Ile Pro Trp Asn Leu Glu Arg Ile Ile	Substitution, Ser>Asn	Missense Mutation	0,038

BE3-gMH (#3_*mPcsk9*)

Alleles	#Reads	Protein Sequence	Type	Outcome	Percentage
ATTGAGGAAGACTCCTTTGTCTTCGCCCAGAGCATCCCATGGAACCTGGAGCGAATTATCC	519 932	Ile Glu Glu Asp Ser Phe Val Phe Ala Gln Ser Ile Pro Trp Asn Leu Glu Arg Ile Ile	Wild type	Wild type	88,400
ATTGAGGAAGACTCCTTTGTCTTCGCCCAGAGCATCCCAT**AA**AACCTGGAGCGAATTATCC	23 054	Ile Glu Glu Asp Ser Phe Val Phe Ala Gln Ser Ile Pro **Stp**	Substitution, Trp>Stp	Nonsense Mutation, Knockout	3,920
ATTGAGGAAGACTCCTTTGTCTTCGCCCAGAGCATCCCATG**A**AACCTGGAGCGAATTATCC	19 038	Ile Glu Glu Asp Ser Phe Val Phe Ala Gln Ser Ile Pro **Stp**	Substitution, Trp>Stp	Nonsense Mutation, Knockout	3,237
ATTGAGGAAGACTCCTTTGTCTTCGCCCAGAGCATCCCAT**TA**AACCTGGAGCGAATTATCC	2 975	Ile Glu Glu Asp Ser Phe Val Phe Ala Gln Ser Ile Pro **Leu** Asn Leu Glu Arg Ile Ile	Substitution, Trp>Leu	Missense Mutation	0,506
ATTGAGGAAGACTCCTTTGTCTTCGCCCAGAGCATCCCATG**T**AACCTGGAGCGAATTATCC	1 826	Ile Glu Glu Asp Ser Phe Val Phe Ala Gln Ser Ile Pro **Cys** Asn Leu Glu Arg Ile Ile	Substitution, Trp>Cys	Missense Mutation	0,310
ATTGAGGAAGACTCCTTTGTCTTCGCCCAGAGCATCCCAT**AC**AACCTGGAGCGAATTATCC	1 346	Ile Glu Glu Asp Ser Phe Val Phe Ala Gln Ser Ile Pro **Tyr** Asn Leu Glu Arg Ile Ile	Substitution, Trp>Tyr	Missense Mutation	0,229
ATTGAGGAAGACTCCTTTGTCTTCGCCCAGAGCATCCCATG**C**AACCTGGAGCGAATTATCC	1 083	Ile Glu Glu Asp Ser Phe Val Phe Ala Gln Ser Ile Pro **Cys** Asn Leu Glu Arg Ile Ile	Substitution, Trp>Cys	Missense Mutation	0,184
ATTGAGGAAGACTCCTTTGTCTTCGCCCAGAGCATCCCAT**AT**AACCTGGAGCGAATTATCC	689	Ile Glu Glu Asp Ser Phe Val Phe Ala Gln Ser Ile Pro **Tyr** Asn Leu Glu Arg Ile Ile	Substitution, Trp>Tyr	Missense Mutation	0,117
ATTGAGGAAGACTCCTTTGTCTTCGCCCAGAGCATCCCAT**A**GAACCTGGAGCGAATTATCC	610	Ile Glu Glu Asp Ser Phe Val Phe Ala Gln Ser Ile Pro **Stp**	Substitution, Trp>Stp	Nonsense Mutation, Knockout	0,104
ATTGAGGAAGACTCCTTTGTCTTCGCCCAGAGCATCCCATG**A**AACCTG**A**AGCGAATTATCC	537	Ile Glu Glu Asp Ser Phe Val Phe Ala Gln Ser Ile Pro **Stp**	Substitution, Trp>Stp	Nonsense Mutation, Knockout	0,091

BE3-gMH (#4_*mPcsk9*)

Alleles	#Reads	Protein Sequence	Type	Outcome	Percentage
ATTGAGGAAGACTCCTTTGTCTTCGCCCAGAGCATCCCATGGAACCTGGAGCGAATTATCC	253 381	Ile Glu Glu Asp Ser Phe Val Phe Ala Gln Ser Ile Pro Trp Asn Leu Glu Arg Ile Ile	Wild type	Wild type	89,700
ATTGAGGAAGACTCCTTTGTCTTCGCCCAGAGCATCCCAT**AA**AACCTGGAGCGAATTATCC	8 799	Ile Glu Glu Asp Ser Phe Val Phe Ala Gln Ser Ile Pro **Stp**	Substitution, Trp>Stp	Nonsense Mutation, Knockout	3,115
ATTGAGGAAGACTCCTTTGTCTTCGCCCAGAGCATCCCATG**A**AACCTGGAGCGAATTATCC	8 089	Ile Glu Glu Asp Ser Phe Val Phe Ala Gln Ser Ile Pro **Stp**	Substitution, Trp>Stp	Nonsense Mutation, Knockout	2,864
ATTGAGGAAGACTCCTTTGTCTTCGCCCAGAGCATCCCATG**T**AACCTGGAGCGAATTATCC	1 385	Ile Glu Glu Asp Ser Phe Val Phe Ala Gln Ser Ile Pro **Cys** Asn Leu Glu Arg Ile Ile	Substitution, Trp>Cys	Missense Mutation	0,490
ATTGAGGAAGACTCCTTTGTCTTCGCCCAGAGCATCCCAT**TA**AACCTGGAGCGAATTATCC	1 265	Ile Glu Glu Asp Ser Phe Val Phe Ala Gln Ser Ile Pro **Leu** Asn Leu Glu Arg Ile Ile	Substitution, Trp>Leu	Missense Mutation	0,448
ATTGAGGAAGACTCCTTTGTCTTCGCCCAGAGCATCCCAT**AC**AACCTGGAGCGAATTATCC	500	Ile Glu Glu Asp Ser Phe Val Phe Ala Gln Ser Ile Pro **Tyr** Asn Leu Glu Arg Ile Ile	Substitution, Trp>Tyr	Missense Mutation	0,177
ATTGAGGAAGACTCCTTTGTCTTCGCCCAGAGCATCCCAT**AT**AACCTGGAGCGAATTATCC	363	Ile Glu Glu Asp Ser Phe Val Phe Ala Gln Ser Ile Pro **Tyr** Asn Leu Glu Arg Ile Ile	Substitution, Trp>Tyr	Missense Mutation	0,129
ATTGAGGAAGACTCCTTTGTCTTCGCCCAGAGCATCCCATG**C**AACCTGGAGCGAATTATCC	346	Ile Glu Glu Asp Ser Phe Val Phe Ala Gln Ser Ile Pro **Cys** Asn Leu Glu Arg Ile Ile	Substitution, Trp>Cys	Missense Mutation	0,122
ATTGAGGAAGACTCCTTTGTCTTCGCCCAGAGCATCCCAT**A**GAACCTGGAGCGAATTATCC	199	Ile Glu Glu Asp Ser Phe Val Phe Ala Gln Ser Ile Pro **Stp**	Substitution, Trp>Stp	Nonsense Mutation, Knockout	0,070
ATTGAGGAAGACTCCTTTGTCTTCGCCCAGAGCATCCCAT**-A**AACCTGGAGCGAATTATCC	166	Ile Glu Glu Asp Ser Phe Val Phe Ala Gln Ser Ile Pro **Stp**	Frameshift deletion	Frameshift Mutation, Knockout	0,059

BE3-gMH (#5_ *mPcsk9*)

Alleles	#Reads	Protein Sequence	Type	Outcome	Percentage
GAGGAAGACTCCTTTGTCTTCGCCCAGAGCATCCCATGGAACCTGGAGCGAATTATCC	143 886	Glu Glu Asp Ser Phe Val Phe Ala Gln Ser Ile Pro Trp Asn Leu Glu Arg Ile Ile	Wild type	Wild type	89,664
GAGGAAGACTCCTTTGTCTTCGCCCAGAGCATCCCAT**AA**AACCTGGAGCGAATTATCC	3 517	Glu Glu Asp Ser Phe Val Phe Ala Gln Ser Ile Pro **Stp**	Substitution, Trp>Stp	Nonsense Mutation, Knockout	2,158
GAGGAAGACTCCTTTGTCTTCGCCCAGAGCATCCCATG**A**AACCTGGAGCGAATTATCC	2 165	Glu Glu Asp Ser Phe Val Phe Ala Gln Ser Ile Pro **Stp**	Substitution, Trp>Stp	Nonsense Mutation, Knockout	1,328
GAGGAAGACTCCTTTGTCTTCGCCCAGAGCATCCCATG**T**AACCTGGAGCGAATTATCC	780	Glu Glu Asp Ser Phe Val Phe Ala Gln Ser Ile Pro Cys Asn Leu Glu Arg Ile Ile	Substitution, Trp>Cys	Missense Mutation	0,479
GAGGAAGACTCCTTTGTCTTCGCCCAGAGCATCC**A**ATGGAACCTGGAGCGAATTATCC	626	Glu Glu Asp Ser Phe Val Phe Ala Gln Ser Ile Gln Trp Asn Leu Glu Arg Ile Ile	Substitution, Pro>Gln	Missense Mutation	0,384
GAGGAAGACTCCTTTGTCTTCGCCCAGAGCATCCCAT**TA**AACCTGGAGCGAATTATCC	604	Glu Glu Asp Ser Phe Val Phe Ala Gln Ser Ile Pro Leu Asn Leu Glu Arg Ile Ile	Substitution, Trp>Leu	Missense Mutation	0,371
GAGGAAGACTCCTTTGTCTTCGCC**A**AGAGCATCCCATGGAACCTGGAGCGAATTATCC	569	Glu Glu Asp Ser Phe Val Phe Ala Lys Ser Ile Pro Trp Asn Leu Glu Arg Ile Ile	Substitution, Gln>Lys	Missense Mutation	0,349
GAGGAAGACTCCTTTGTCTTCGCCCAGAGCATCCCATGGAACCTG**T**AGCGAATTATCC	444	Glu Glu Asp Ser Phe Val Phe Ala Gln Ser Ile Pro Trp Asn Leu **Stp**	Substitution, Glu>Stp	Nonsense Mutation, Knockout	0,272
GAGGAAGACTCCTTTGTCTTCGCCCAGAGCATCCCATGGAACCTGGAGC**T**AATTATCC	397	Glu Glu Asp Ser Phe Val Phe Ala Gln Ser Ile Pro Trp Asn Leu Glu Leu Ile Ile	Substitution, Arg>Leu	Missense Mutation	0,244
GAGGAAGACTCCTTTGTCTTCGCCCA**T**AGCATCCCATGGAACCTGGAGCGAATTATCC	396	Glu Glu Asp Ser Phe Val Phe Ala His Ser Ile Pro Trp Asn Leu Glu Arg Ile Ile	Substitution, Gln>His	Missense Mutation	0,243

BE3-gMH (#6_ *mPcsk9*)

Alleles	#Reads	Protein Sequence	Type	Outcome	Percentage
GAGGAAGACTCCTTTGTCTTCGCCCAGAGCATCCCATGGAACCTGGAGCGAATTATCC	171 461	Glu Glu Asp Ser Phe Val Phe Ala Gln Ser Ile Pro Trp Asn Leu Glu Arg Ile Ile	Wild type	Wild type	88,877
GAGGAAGACTCCTTTGTCTTCGCCCAGAGCATCCCAT**AA**AACCTGGAGCGAATTATCC	7 696	Glu Glu Asp Ser Phe Val Phe Ala Gln Ser Ile Pro **Stp**	Substitution, Trp>Stp	Nonsense Mutation, Knockout	3,989
GAGGAAGACTCCTTTGTCTTCGCCCAGAGCATCCCATG**A**AACCTGGAGCGAATTATCC	4 243	Glu Glu Asp Ser Phe Val Phe Ala Gln Ser Ile Pro **Stp**	Substitution, Trp>Stp	Nonsense Mutation, Knockout	2,199
GAGGAAGACTCCTTTGTCTTCGCCCAGAGCATCCCAT**TA**AACCTGGAGCGAATTATCC	970	Glu Glu Asp Ser Phe Val Phe Ala Gln Ser Ile Pro Leu Asn Leu Glu Arg Ile Ile	Substitution, Trp>Leu	Missense Mutation	0,503
GAGGAAGACTCCTTTGTCTTCGCCCAGAGCATCCCATG**T**AACCTGGAGCGAATTATCC	915	Glu Glu Asp Ser Phe Val Phe Ala Gln Ser Ile Pro Cys Asn Leu Glu Arg Ile Ile	Substitution, Trp>Cys	Missense Mutation	0,474
GAGGAAGACTCCTTTGTCTTCGCCCAGAGCATCC**A**ATGGAACCTGGAGCGAATTATCC	616	Glu Glu Asp Ser Phe Val Phe Ala Gln Ser Ile Gln Trp Asn Leu Glu Arg Ile Ile	Substitution, Pro>Gln	Missense Mutation	0,319
GAGGAAGACTCCTTTGTCTTCGCC**A**AGAGCATCCCATGGAACCTGGAGCGAATTATCC	581	Glu Glu Asp Ser Phe Val Phe Ala Lys Ser Ile Pro Trp Asn Leu Glu Arg Ile Ile	Substitution, Gln>Lys	Missense Mutation	0,301
GAGGAAGACTCCTTTGTCTTCGCCCAGAGCATCCCATGGAACCTG**T**AGCGAATTATCC	501	Glu Glu Asp Ser Phe Val Phe Ala Gln Ser Ile Pro Trp Asn Leu **Stp**	Substitution, Glu>Stp	Nonsense Mutation, Knockout	0,260
GAGGAAGACTCCTTTGTCTTCGCCCAGAGCATCCCATGGAACCTGGAGC**T**AATTATCC	390	Glu Glu Asp Ser Phe Val Phe Ala Gln Ser Ile Pro Trp Asn Leu Glu Leu Ile Ile	Substitution, Arg>Leu	Missense Mutation	0,202
GAGGAAGACTCCTTTGTCTTCGCCCA**T**AGCATCCCATGGAACCTGGAGCGAATTATCC	386	Glu Glu Asp Ser Phe Val Phe Ala His Ser Ile Pro Trp Asn Leu Glu Arg Ile Ile	Substitution, Gln>His	Missense Mutation	0,200

BE3-gMH (#7_ *mPcsk9*)

Alleles	#Reads	Protein Sequence	Type	Outcome	Percentage
GAGGAAGACTCCTTTGTCTTCGCCCAGAGCATCCCATGGAACCTGGAGCGAATTATCC	169 754	Glu Glu Asp Ser Phe Val Phe Ala Gln Ser Ile Pro Trp Asn Leu Glu Arg Ile Ile	Wild type	Wild type	85,585
GAGGAAGACTCCTTTGTCTTCGCCCAGAGCATCCCAT**AA**AACCTGGAGCGAATTATCC	11 599	Glu Glu Asp Ser Phe Val Phe Ala Gln Ser Ile Pro **Stp**	Substitution, Trp>Stp	Nonsense Mutation, Knockout	5,848
GAGGAAGACTCCTTTGTCTTCGCCCAGAGCATCCCATG**A**AACCTGGAGCGAATTATCC	7 422	Glu Glu Asp Ser Phe Val Phe Ala Gln Ser Ile Pro **Stp**	Substitution, Trp>Stp	Nonsense Mutation, Knockout	3,742
GAGGAAGACTCCTTTGTCTTCGCCCAGAGCATCCCAT**TA**AACCTGGAGCGAATTATCC	1 591	Glu Glu Asp Ser Phe Val Phe Ala Gln Ser Ile Pro Leu Asn Leu Glu Arg Ile Ile	Substitution, Trp>Leu	Missense Mutation	0,802
GAGGAAGACTCCTTTGTCTTCGCCCAGAGCATCCCATG**T**AACCTGGAGCGAATTATCC	1 417	Glu Glu Asp Ser Phe Val Phe Ala Gln Ser Ile Pro Cys Asn Leu Glu Arg Ile Ile	Substitution, Trp>Cys	Missense Mutation	0,714
GAGGAAGACTCCTTTGTCTTCGCCCAGAGCATCC**A**ATGGAACCTGGAGCGAATTATCC	654	Glu Glu Asp Ser Phe Val Phe Ala Gln Ser Ile Gln Trp Asn Leu Glu Arg Ile Ile	Substitution, Pro>Gln	Missense Mutation	0,330
GAGGAAGACTCCTTTGTCTTCGCC**A**AGAGCATCCCATGGAACCTGGAGCGAATTATCC	592	Glu Glu Asp Ser Phe Val Phe Ala Lys Ser Ile Pro Trp Asn Leu Glu Arg Ile Ile	Substitution, Gln>Lys	Missense Mutation	0,298
GAGGAAGACTCCTTTGTCTTCGCCCAGAGCATCCCATGGAACCTGTAGCGAATTATCC	500	Glu Glu Asp Ser Phe Val Phe Ala Gln Ser Ile Pro Trp Asn Leu **Stp**	Substitution, Glu>Stp	Nonsense Mutation, Knockout	0,252
GAGGAAGACTCCTTTGTCTTCGCCCA**T**AGCATCCCATGGAACCTGGAGCGAATTATCC	428	Glu Glu Asp Ser Phe Val Phe Ala His Ser Ile Pro Trp Asn Leu Glu Arg Ile Ile	Substitution, Gln>His	Missense Mutation	0,216
GAGGAAGACTCCTTTGTCTTCGCCCAGAGCATCCCATGGAACCTGGAGCTAATTATCC	428	Glu Glu Asp Ser Phe Val Phe Ala Gln Ser Ile Pro Trp Asn Leu Glu Leu Ile Ile	Substitution, Arg>Leu	Missense Mutation	0,216

BE3-MH (#8_ *mPcsk9*)

Alleles	#Reads	Protein Sequence	Type	Outcome	Percentage
GAGGAAGACTCCTTTGTCTTCGCCCAGAGCATCCCATGGAACCTGGAGCGAATTATCC	182 283	Glu Glu Asp Ser Phe Val Phe Ala Gln Ser Ile Pro Trp Asn Leu Glu Arg Ile Ile	Wild type	Wild type	88,134
GAGGAAGACTCCTTTGTCTTCGCCCAGAGCATCCCAT**AA**AACCTGGAGCGAATTATCC	6 364	Glu Glu Asp Ser Phe Val Phe Ala Gln Ser Ile Pro **Stp**	Substitution, Trp>Stp	Nonsense Mutation, Knockout	3,077
GAGGAAGACTCCTTTGTCTTCGCCCAGAGCATCCCATG**A**AACCTGGAGCGAATTATCC	4 316	Glu Glu Asp Ser Phe Val Phe Ala Gln Ser Ile Pro **Stp**	Substitution, Trp>Stp	Nonsense Mutation, Knockout	2,087
GAGGAAGACTCCTTTGTCTTCGCCCAGAGCATCCCAT**TA**AACCTGGAGCGAATTATCC	1 052	Glu Glu Asp Ser Phe Val Phe Ala Gln Ser Ile Pro Leu Asn Leu Glu Arg Ile Ile	Substitution, Trp>Leu	Missense Mutation	0,509
GAGGAAGACTCCTTTGTCTTCGCCCAGAGCATCCCATG**T**AACCTGGAGCGAATTATCC	861	Glu Glu Asp Ser Phe Val Phe Ala Gln Ser Ile Pro Cys Asn Leu Glu Arg Ile Ile	Substitution, Trp>Cys	Missense Mutation	0,416
GAGGAAGACTCCTTTGTCTTCGCCCAGAGCATCC**A**ATGGAACCTGGAGCGAATTATCC	491	Glu Glu Asp Ser Phe Val Phe Ala Gln Ser Ile Gln Trp Asn Leu Glu Arg Ile Ile	Substitution, Pro>Gln	Missense Mutation	0,237
GAGGAAGACTCCTTTGTCTTCGCC**A**AGAGCATCCCATGGAACCTGGAGCGAATTATCC	430	Glu Glu Asp Ser Phe Val Phe Ala Lys Ser Ile Pro Trp Asn Leu Glu Arg Ile Ile	Substitution, Gln>Lys	Missense Mutation	0,208
GAGGAAGACTCCTTTGTCTTCGCCCAGAGCATCCCATGGAACCTG**T**AGCGAATTATCC	370	Glu Glu Asp Ser Phe Val Phe Ala Gln Ser Ile Pro Trp Asn Leu **Stp**	Substitution, Glu>Stp	Nonsense Mutation, Knockout	0,179
GAGGAAGACTCCTTTGTCTTCGCCCAGAGCATCCCATGGAACCTGGAGC**T**AATTATCC	340	Glu Glu Asp Ser Phe Val Phe Ala Gln Ser Ile Pro Trp Asn Leu Glu Leu Ile Ile	Substitution, Arg>Leu	Missense Mutation	0,164
GAGGAAGACTCCTTTGTCTTCGCCCA**T**AGCATCCCATGGAACCTGGAGCGAATTATCC	282	Glu Glu Asp Ser Phe Val Phe Ala His Ser Ile Pro Trp Asn Leu Glu Arg Ile Ile	Substitution, Gln>His	Missense Mutation	0,136

BE3-gMH (#1_*hPCSK9*)

Alleles	#Reads	Protein Sequence	Type	Outcome	Percentage
TACATCGAGGAGGACTCCTCTGTCTTTGCCCAGAGCATCCCGTGGAACCTGGAGCGGATTACCCCTC	277 920	Tyr Ile Glu Glu Asp Ser Ser Val Phe Ala Gln Ser Ile Pro Trp Asn Leu Glu Arg Ile Thr Pro	Wild type	Wild type	88,891
TACATCGAGGAGGACTCCTCTGTCTTTGCCCAGAGCATCCCGT**AA**AACCTGGAGCGGATTACCCCTC	16 328	Tyr Ile Glu Glu Asp Ser Ser Val Phe Ala Gln Ser Ile Pro **Stp**	Substitution, Trp>Stp	Nonsense Mutation, Knockout	5,222
TACATCGAGGAGGACTCCTCTGTCTTTGCCCAGAGCATCCCGTG**A**AACCTGGAGCGGATTACCCCTC	4 763	Tyr Ile Glu Glu Asp Ser Ser Val Phe Ala Gln Ser Ile Pro **Stp**	Substitution, Trp>Stp	Nonsense Mutation, Knockout	1,523
TACATCGAGGAGGACTCCTCTGTCTTTGCCCAGAGCATCCCGT**TA**AACCTGGAGCGGATTACCCCTC	807	Tyr Ile Glu Glu Asp Ser Ser Val Phe Ala Gln Ser Ile Pro Leu Asn Leu Glu Arg Ile Thr Pro	Substitution, Trp>Leu	Missense Mutation	0,258
TACATCGAGGAGGACTCCTCTGTCTTTGCCCAGAGCATCCC**A**T**AA**AACCTGGAGCGGATTACCCCTC	454	Tyr Ile Glu Glu Asp Ser Ser Val Phe Ala Gln Ser Ile Pro **Stp**	Substitution, Trp>Stp	Nonsense Mutation, Knockout	0,145
TACATCGAGGAGGACTCCTCTGTCTTTGCCCAGAGCATCCCGT**AT**AACCTGGAGCGGATTACCCCTC	453	Tyr Ile Glu Glu Asp Ser Ser Val Phe Ala Gln Ser Ile Pro Tyr Asn Leu Glu Arg Ile Thr Pro	Substitution, Trp>Tyr	Missense Mutation	0,145
TACATCGAGGAGGACTCCTCTGTCTTTGCCCAGAGCATCCCGT**A**GAACCTGGAGCGGATTACCCCTC	406	Tyr Ile Glu Glu Asp Ser Ser Val Phe Ala Gln Ser Ile Pro **Stp**	Substitution, Trp>Stp	Nonsense Mutation, Knockout	0,130
TACATCGAGGAGGACTCCTCTGTCTTTGCCCAGAGCATCCCGTG**T**AACCTGGAGCGGATTACCCCTC	394	Tyr Ile Glu Glu Asp Ser Ser Val Phe Ala Gln Ser Ile Pro Cys Asn Leu Glu Arg Ile Thr Pro	Substitution, Trp>Cys	Missense Mutation	0,126
TACATCGAGGAGGACTCCTCTGTCTTTGCCCAGAGCATCCCGT**AA**AACCTG**A**AGCGGATTACCCCTC	307	Tyr Ile Glu Glu Asp Ser Ser Val Phe Ala Gln Ser Ile Pro **Stp**	Substitution, Trp>Stp	Nonsense Mutation, Knockout	0,098
TACATCGAGGAGGACTCCTCTGTCTTTGCCCA------------GAACCTGGAGCGGATTACCCCTC	219	Tyr Ile Glu Glu Asp Ser Ser Val Phe Ala Gln Asn Leu Glu Arg Ile Thr Pro	Inframe deletion	Inframe Mutation	0,070

BE3-gMH (#2_*hPCSK9*)

Alleles	#Reads	Protein Sequence	Type	Outcome	Percentage
TACATCGAGGAGGACTCCTCTGTCTTTGCCCAGAGCATCCCGTGGAACCTGGAGCGGATTACCCCTC	326 812	Tyr Ile Glu Glu Asp Ser Ser Val Phe Ala Gln Ser Ile Pro Trp Asn Leu Glu Arg Ile Thr Pro	Wild type	Wild type	85,594
TACATCGAGGAGGACTCCTCTGTCTTTGCCCAGAGCATCCCGT**AA**AACCTGGAGCGGATTACCCCTC	24 429	Tyr Ile Glu Glu Asp Ser Ser Val Phe Ala Gln Ser Ile Pro **Stp**	Substitution, Trp>Stp	Nonsense Mutation, Knockout	6,398
TACATCGAGGAGGACTCCTCTGTCTTTGCCCAGAGCATCCCGTG**A**AACCTGGAGCGGATTACCCCTC	8 446	Tyr Ile Glu Glu Asp Ser Ser Val Phe Ala Gln Ser Ile Pro **Stp**	Substitution, Trp>Stp	Nonsense Mutation, Knockout	2,212
TACATCGAGGAGGACTCCTCTGTCTTTGCCCAGAGCATCCC**A**T**AA**AACCTGGAGCGGATTACCCCTC	1 592	Tyr Ile Glu Glu Asp Ser Ser Val Phe Ala Gln Ser Ile Pro **Stp**	Substitution, Trp>Stp	Nonsense Mutation, Knockout	0,417
TACATCGAGGAGGACTCCTCTGTCTTTGCCCAGAGCATCCCGT**A**GAACCTGGAGCGGATTACCCCTC	1 067	Tyr Ile Glu Glu Asp Ser Ser Val Phe Ala Gln Ser Ile Pro **Stp**	Substitution, Trp>Stp	Nonsense Mutation, Knockout	0,279
TACATCGAGGAGGACTCCTCTGTCTTTGCCCAGAGCATCCCGT-**A**AACCTGGAGCGGATTACCCCTC	971	Tyr Ile Glu Glu Asp Ser Ser Val Phe Ala Gln Ser Ile Pro **Stp**	Frameshift deletion	Frameshift Mutation, Knockout	0,254
TACATCGAGGAGGACTCCTCTGTCTTTGCCCAGAGCATCCCGTG**T**AACCTGGAGCGGATTACCCCTC	803	Tyr Ile Glu Glu Asp Ser Ser Val Phe Ala Gln Ser Ile Pro Cys Asn Leu Glu Arg Ile Thr Pro	Substitution, Trp>Cys	Missense Mutation	0,210
TACATCGAGGAGGACTCCTCTGTCTTTGCCCAGAGCATCCCGT**TA**AACCTGGAGCGGATTACCCCTC	569	Tyr Ile Glu Glu Asp Ser Ser Val Phe Ala Gln Ser Ile Pro Leu Asn Leu Glu Arg Ile Thr Pro	Substitution, Trp>Leu	Missense Mutation	0,149
TACATCGAGGAGGACTCCTCTGTCTTTGCCCAGAGCATCCC**A**T-**A**AACCTGGAGCGGATTACCCCTC	511	Tyr Ile Glu Glu Asp Ser Ser Val Phe Ala Gln Ser Ile Pro **Stp**	Frameshift deletion	Frameshift Mutation, Knockout	0,134
TACATCGAGGAGGACTCCTCTGTCTTTGCCCAGAGCATCCCGT**AA**AACCTG**A**AGCGGATTACCCCTC	485	Tyr Ile Glu Glu Asp Ser Ser Val Phe Ala Gln Ser Ile Pro **Stp**	Substitution, Trp>Stp	Nonsense Mutation, Knockout	0,127

BE3-gMH (#3_*hPCSK9*)

Alleles	#Reads	Protein Sequence	Type	Outcome	Percentage
TACATCGAGGAGGACTCCTCTGTCTTTGCCCAGAGCATCCCGTGGAACCTGGAGCGGATTACCCCTC	294 948	Tyr Ile Glu Glu Asp Ser Ser Val Phe Ala Gln Ser Ile Pro Trp Asn Leu Glu Arg Ile Thr Pro	Wild type	Wild type	78,889
TACATCGAGGAGGACTCCTCTGTCTTTGCCCAGAGCATCCCGT**AA**AACCTGGAGCGGATTACCCCTC	36 172	Tyr Ile Glu Glu Asp Ser Ser Val Phe Ala Gln Ser Ile Pro **Stp**	Substitution, Trp>Stp	Nonsense Mutation, Knockout	9,675
TACATCGAGGAGGACTCCTCTGTCTTTGCCCAGAGCATCCCGTG**A**AACCTGGAGCGGATTACCCCTC	12 770	Tyr Ile Glu Glu Asp Ser Ser Val Phe Ala Gln Ser Ile Pro **Stp**	Substitution, Trp>Stp	Nonsense Mutation, Knockout	3,416
TACATCGAGGAGGACTCCTCTGTCTTTGCCCAGAGCATCCCGT**TA**AACCTGGAGCGGATTACCCCTC	3 201	Tyr Ile Glu Glu Asp Ser Ser Val Phe Ala Gln Ser Ile Pro Leu Asn Leu Glu Arg Ile Thr Pro	Substitution, Trp>Leu	Missense Mutation	0,856
TACATCGAGGAGGACTCCTCTGTCTTTGCCCAGAGCATCCCGTG**T**AACCTGGAGCGGATTACCCCTC	1 728	Tyr Ile Glu Glu Asp Ser Ser Val Phe Ala Gln Ser Ile Pro Cys Asn Leu Glu Arg Ile Thr Pro	Substitution, Trp>Cys	Missense Mutation	0,462
TACATCGAGGAGGACTCCTCTGTCTTTGCCCAGAGCATCCC**A**T**AA**AACCTGGAGCGGATTACCCCTC	1 208	Tyr Ile Glu Glu Asp Ser Ser Val Phe Ala Gln Ser Ile Pro **Stp**	Substitution, Trp>Stp	Nonsense Mutation, Knockout	0,323
TACATCGAGGAGGACTCCTCTGTCTTTGCCCAGAGCATCCCGTG**A**AACCTG**A**AGCGGATTACCCCTC	702	Tyr Ile Glu Glu Asp Ser Ser Val Phe Ala Gln Ser Ile Pro **Stp**	Substitution, Trp>Stp	Nonsense Mutation, Knockout	0,188
TACATCGAGGAGGACTCCTCTGTCTTTGCCCAGAGCATCCCGT-**A**AACCTGGAGCGGATTACCCCTC	663	Tyr Ile Glu Glu Asp Ser Ser Val Phe Ala Gln Ser Ile Pro **Stp**	Frameshift deletion	Frameshift Mutation, Knockout	0,177
TACATCGAGGAGGACTCCTCTGTCTTTGCCCAGAGCATCCCGT**AT**AACCTGGAGCGGATTACCCCTC	509	Tyr Ile Glu Glu Asp Ser Ser Val Phe Ala Gln Ser Ile Pro Tyr Asn Leu Glu Arg Ile Thr Pro	Substitution, Trp>Tyr	Missense Mutation	0,136
TACATCGAGGAGGACTCCTCTGTCTTTGCCCAGA-------GTGGAACCTGGAGCGGATTACCCCTC	499	Tyr Ile Glu Glu Asp Ser Ser Val Phe Ala Gln Ser Gly Thr Trp Ser Gly Leu Pro Leu	Frameshift deletion	Frameshift Mutation	0,133

BE3-gMH (#4_*hPCSK9*)

Alleles	#Reads	Protein Sequence	Type	Outcome	Percentage
TACATCGAGGAGGACTCCTCTGTCTTTGCCCAGAGCATCCCGTGGAACCTGGAGCGGATTACCCCTC	249 720	Tyr Ile Glu Glu Asp Ser Ser Val Phe Ala Gln Ser Ile Pro Trp Asn Leu Glu Arg Ile Thr Pro	Wild type	Wild type	75,923
TACATCGAGGAGGACTCCTCTGTCTTTGCCCAGAGCATCCCGT**AA**AACCTGGAGCGGATTACCCCTC	40 347	Tyr Ile Glu Glu Asp Ser Ser Val Phe Ala Gln Ser Ile Pro **Stp**	Substitution, Trp>Stp	Nonsense Mutation, Knockout	12,267
TACATCGAGGAGGACTCCTCTGTCTTTGCCCAGAGCATCCCGTG**A**AACCTGGAGCGGATTACCCCTC	11 246	Tyr Ile Glu Glu Asp Ser Ser Val Phe Ala Gln Ser Ile Pro **Stp**	Substitution, Trp>Stp	Nonsense Mutation, Knockout	3,419
TACATCGAGGAGGACTCCTCTGTCTTTGCCCAGAGCATCCCGT**TA**AACCTGGAGCGGATTACCCCTC	4 012	Tyr Ile Glu Glu Asp Ser Ser Val Phe Ala Gln Ser Ile Pro Leu Asn Leu Glu Arg Ile Thr Pro	Substitution, Trp>Leu	Missense Mutation	1,220
TACATCGAGGAGGACTCCTCTGTCTTTGCCCAGAGCATCCC**A**T**AA**AACCTGGAGCGGATTACCCCTC	1 830	Tyr Ile Glu Glu Asp Ser Ser Val Phe Ala Gln Ser Ile Pro **Stp**	Substitution, Trp>Stp	Nonsense Mutation, Knockout	0,556
TACATCGAGGAGGACTCCTCTGTCTTTGCCCAGAGCATCCCGT-**A**AACCTGGAGCGGATTACCCCTC	902	Tyr Ile Glu Glu Asp Ser Ser Val Phe Ala Gln Ser Ile Pro **Stp**	Frameshift deletion	Frameshift Mutation, Knockout	0,274
TACATCGAGGAGGACTCCTCTGTCTTTGCCCAGAGCATCCCGTG**T**AACCTGGAGCGGATTACCCCTC	848	Tyr Ile Glu Glu Asp Ser Ser Val Phe Ala Gln Ser Ile Pro Cys Asn Leu Glu Arg Ile Thr Pro	Substitution, Trp>Cys	Missense Mutation	0,258
TACATCGAGGAGGACTCCTCTGTCTTTGCCCAGAGCATCCCGT**AA**AACCTG**A**AGCGGATTACCCCTC	847	Tyr Ile Glu Glu Asp Ser Ser Val Phe Ala Gln Ser Ile Pro **Stp**	Substitution, Trp>Stp	Nonsense Mutation, Knockout	0,258
TACATCGAGGAGGACTCCTCTGTCTTTGCCCAGAGCATCCCGT**A**GAACCTGGAGCGGATTACCCCTC	567	Tyr Ile Glu Glu Asp Ser Ser Val Phe Ala Gln Ser Ile Pro **Stp**	Substitution, Trp>Stp	Nonsense Mutation, Knockout	0,172
TACATCGAGGAGGACTCCTCTGTCTTTGCCCAGAGCATCCC**A**TG**A**AACCTGGAGCGGATTACCCCTC	550	Tyr Ile Glu Glu Asp Ser Ser Val Phe Ala Gln Ser Ile Pro **Stp**	Substitution, Trp>Stp	Nonsense Mutation, Knockout	0,167

BE3-gMH (#5_ *hPCSK9*)

Alleles	#Reads	Protein Sequence	Type	Outcome	Percentage
TACATCGAGGAGGACTCCTCTGTCTTTGCCCAGAGCATCCCGTGGAACCTGGAGCGGATTACCCCTC	166 491	Tyr Ile Glu Glu Asp Ser Ser Val Phe Ala Gln Ser Ile Pro Trp Asn Leu Glu Arg Ile Thr Pro	Wild type	Wild type	74,868
TACATCGAGGAGGACTCCTCTGTCTTTGCCCAGAGCATCCCGTAAAACCTGGAGCGGATTACCCCTC	11 391	Tyr Ile Glu Glu Asp Ser Ser Val Phe Ala Gln Ser Ile Pro Stp	Substitution, Trp>Stp	Nonsense Mutation, Knockout	5,122
TACATCGAGGAGGACTCCTCTGTCTTTGCCCAGAGCATCCCGTGAAACCTGGAGCGGATTACCCCTC	4 289	Tyr Ile Glu Glu Asp Ser Ser Val Phe Ala Gln Ser Ile Pro Stp	Substitution, Trp>Stp	Nonsense Mutation, Knockout	1,929
TACATCGAGGAGGACTCCTCTGTCTTTGCCCAGAGCATCCCGTGGAACCTGGAGCGGATTACCACTC	1 630	Tyr Ile Glu Glu Asp Ser Ser Val Phe Ala Gln Ser Ile Pro Trp Asn Leu Glu Arg Ile Thr Thr	Substitution, Pro>Thr	Missense Mutation	0,733
TACATCGAGGAGGACTCCTCTGTCTTTGCCCAGAGCATCCAGTGGAACCTGGAGCGGATTACCCCTC	1593	Tyr Ile Glu Glu Asp Ser Ser Val Phe Ala Gln Ser Ile Gln Trp Asn Leu Glu Arg Ile Thr Pro	Substitution, Pro>Gln	Missense Mutation	0,716
TACATCGAGGAGGACTCCTCTGTCTTTGCCCAGAGCATCCCGTGGAACCTGTAGCGGATTACCCCTC	1 513	Tyr Ile Glu Glu Asp Ser Ser Val Phe Ala Gln Ser Ile Pro Trp Asn Leu Stp	Substitution, Glu>Stp	Nonsense Mutation, Knockout	0,680
TACATCGAGGAGGACTCCTCTGTCTTTGCCCAGAGCATCCCGTGTAACCTGGAGCGGATTACCCCTC	1 410	Tyr Ile Glu Glu Asp Ser Ser Val Phe Ala Gln Ser Ile Pro Cys Asn Leu Glu Arg Ile Thr Pro	Substitution, Trp>Cys	Missense Mutation	0,634
TACATCGAGGAGGACTCCTCTGTCTTTGCCCAGAGCATCCCGTGGAACCTGGAGCGGATTACCCATC	1 337	Tyr Ile Glu Glu Asp Ser Ser Val Phe Ala Gln Ser Ile Pro Trp Asn Leu Glu Arg Ile Thr His	Substitution, Pro>His	Missense Mutation	0,601
TACATCGAGGAGGACTCCTCTGTCTTTGCCAAGAGCATCCCGTGGAACCTGGAGCGGATTACCCCTC	1 249	Tyr Ile Glu Glu Asp Ser Ser Val Phe Ala Lys Ser Ile Pro Trp Asn Leu Glu Arg Ile Thr Pro	Substitution, Gln>Lys	Missense Mutation	0,562
TACATCGAGGAGGACTCCTCTGTCTTTGCCCATAGCATCCCGTGGAACCTGGAGCGGATTACCCCTC	1 171	Tyr Ile Glu Glu Asp Ser Ser Val Phe Ala His Ser Ile Pro Trp Asn Leu Glu Arg Ile Thr Pro	Substitution, Gln>His	Missense Mutation	0,527

BE3-gMH (#6_ *hPCSK9*)

Alleles	#Reads	Protein Sequence	Type	Outcome	Percentage
TACATCGAGGAGGACTCCTCTGTCTTTGCCCAGAGCATCCCGTGGAACCTGGAGCGGATTACCCCTC	222 167	Tyr Ile Glu Glu Asp Ser Ser Val Phe Ala Gln Ser Ile Pro Trp Asn Leu Glu Arg Ile Thr Pro	Wild type	Wild type	74,951
TACATCGAGGAGGACTCCTCTGTCTTTGCCCAGAGCATCCCGTAAAACCTGGAGCGGATTACCCCTC	30 559	Tyr Ile Glu Glu Asp Ser Ser Val Phe Ala Gln Ser Ile Pro Stp	Substitution, Trp>Stp	Nonsense Mutation, Knockout	10,309
TACATCGAGGAGGACTCCTCTGTCTTTGCCCAGAGCATCCCGTGAAACCTGGAGCGGATTACCCCTC	9 416	Tyr Ile Glu Glu Asp Ser Ser Val Phe Ala Gln Ser Ile Pro Stp	Substitution, Trp>Stp	Nonsense Mutation, Knockout	3,177
TACATCGAGGAGGACTCCTCTGTCTTTGCCCAGAGCATCCCGTTAAACCTGGAGCGGATTACCCCTC	1 604	Tyr Ile Glu Glu Asp Ser Ser Val Phe Ala Gln Ser Ile Pro Leu Asn Leu Glu Arg Ile Thr Pro	Substitution, Trp>Leu	Missense Mutation	0,541
TACATCGAGGAGGACTCCTCTGTCTTTGCCCAGAGCATCCCGTGTAACCTGGAGCGGATTACCCCTC	1406	Tyr Ile Glu Glu Asp Ser Ser Val Phe Ala Gln Ser Ile Pro Cys Asn Leu Glu Arg Ile Thr Pro	Substitution, Trp>Cys	Missense Mutation	0,474
TACATCGAGGAGGACTCCTCTGTCTTTGCCCAGAGCATCCCATAAAACCTGGAGCGGATTACCCCTC	1 314	Tyr Ile Glu Glu Asp Ser Ser Val Phe Ala Gln Ser Ile Pro Stp	Substitution, Trp>Stp	Nonsense Mutation, Knockout	0,443
TACATCGAGGAGGACTCCTCTGTCTTTGCCCAGAGCATCCCGTGGAACCTGGAGCGGATTACCACTC	814	Tyr Ile Glu Glu Asp Ser Ser Val Phe Ala Gln Ser Ile Pro Trp Asn Leu Glu Arg Ile Thr Thr	Substitution, Pro>Thr	Missense Mutation	0,275
TACATCGAGGAGGACTCCTCTGTCTTTGCCCAGAGCATCCAGTGGAACCTGGAGCGGATTACCCCTC	748	Tyr Ile Glu Glu Asp Ser Ser Val Phe Ala Gln Ser Ile Gln Trp Asn Leu Glu Arg Ile Thr Pro	Substitution, Pro>Gln	Missense Mutation	0,252
TACATCGAGGAGGACTCCTCTGTCTTTGCCCAGAGCATCCCGTGGAACCTGGAGCGGATTACCCATC	712	Tyr Ile Glu Glu Asp Ser Ser Val Phe Ala Gln Ser Ile Pro Trp Asn Leu Glu Arg Ile Thr His	Substitution, Pro>His	Missense Mutation	0,240

BE3-gMH (#7_ *hPCSK9*)

Alleles	#Reads	Protein Sequence	Type	Outcome	Percentage
TACATCGAGGAGGACTCCTCTGTCTTTGCCCAGAGCATCCCGTGGAACCTGGAGCGGATTACCCCTC	200 394	Tyr Ile Glu Glu Asp Ser Ser Val Phe Ala Gln Ser Ile Pro Trp Asn Leu Glu Arg Ile Thr Pro	Wild type	Wild type	65,069
TACATCGAGGAGGACTCCTCTGTCTTTGCCCAGAGCATCCCGTAAAACCTGGAGCGGATTACCCCTC	47 002	Tyr Ile Glu Glu Asp Ser Ser Val Phe Ala Gln Ser Ile Pro Stp	Substitution, Trp>Stp	Nonsense Mutation, Knockout	15,262
TACATCGAGGAGGACTCCTCTGTCTTTGCCCAGAGCATCCCGTGAAACCTGGAGCGGATTACCCCTC	16 648	Tyr Ile Glu Glu Asp Ser Ser Val Phe Ala Gln Ser Ile Pro Stp	Substitution, Trp>Stp	Nonsense Mutation, Knockout	5,406
TACATCGAGGAGGACTCCTCTGTCTTTGCCCAGAGCATCCCGTTAAACCTGGAGCGGATTACCCCTC	3 003	Tyr Ile Glu Glu Asp Ser Ser Val Phe Ala Gln Ser Ile Pro Leu Asn Leu Glu Arg Ile Thr Pro	Substitution, Trp>Leu	Missense Mutation	0,975
TACATCGAGGAGGACTCCTCTGTCTTTGCCCAGAGCATCCCATAAAACCTGGAGCGGATTACCCCTC	2056	Tyr Ile Glu Glu Asp Ser Ser Val Phe Ala Gln Ser Ile Pro Stp	Substitution, Trp>Stp	Nonsense Mutation, Knockout	0,668
TACATCGAGGAGGACTCCTCTGTCTTTGCCCAGAGCATCCCGTGTAACCTGGAGCGGATTACCCCTC	2 042	Tyr Ile Glu Glu Asp Ser Ser Val Phe Ala Gln Ser Ile Pro Cys Asn Leu Glu Arg Ile Thr Pro	Substitution, Trp>Cys	Missense Mutation	0,663
TACATCGAGGAGGACTCCTCTGTCTTTGCCCAGAGCATCCCGTAAAACCTGAAGCGGATTACCCCTC	1 060	Tyr Ile Glu Glu Asp Ser Ser Val Phe Ala Gln Ser Ile Pro Stp	Substitution, Trp>Stp	Nonsense Mutation, Knockout	0,344
TACATCGAGGAGGACTCCTCTGTCTTTGCCCAGAGCATCCCGTATAACCTGGAGCGGATTACCCCTC	894	Tyr Ile Glu Glu Asp Ser Ser Val Phe Ala Gln Ser Ile Pro Tyr Asn Leu Glu Arg Ile Thr Pro	Substitution, Trp>Tyr	Missense Mutation	0,290
TACATCGAGGAGGACTCCTCTGTCTTTGCCCAGAGCATCCCGT-AAACCTGGAGCGGATTACCCCTC	784	Tyr Ile Glu Glu Asp Ser Ser Val Phe Ala Gln Ser Ile Pro Stp	Frameshift deletion	Frameshift Mutation, Knockout	0,255
TACATCGAGGAGGACTCCTCTGTCTTTGCCCAGAGCATCCCGTGGAACCTGGAGCGGATTACCACTC	752	Tyr Ile Glu Glu Asp Ser Ser Val Phe Ala Gln Ser Ile Pro Trp Asn Leu Glu Arg Ile Thr Thr	Substitution, Pro>Thr	Missense Mutation	0,244

BE3-gMH (#8_ *hPCSK9*)

Alleles	#Reads	Protein Sequence	Type	Outcome	Percentage
TACATCGAGGAGGACTCCTCTGTCTTTGCCCAGAGCATCCCGTGGAACCTGGAGCGGATTACCCCTC	246 592	Tyr Ile Glu Glu Asp Ser Ser Val Phe Ala Gln Ser Ile Pro Trp Asn Leu Glu Arg Ile Thr Pro	Wild type	Wild type	71,707
TACATCGAGGAGGACTCCTCTGTCTTTGCCCAGAGCATCCCGTAAAACCTGGAGCGGATTACCCCTC	33 694	Tyr Ile Glu Glu Asp Ser Ser Val Phe Ala Gln Ser Ile Pro Stp	Substitution, Trp>Stp	Nonsense Mutation, Knockout	9,798
TACATCGAGGAGGACTCCTCTGTCTTTGCCCAGAGCATCCCGTGAAACCTGGAGCGGATTACCCCTC	13 166	Tyr Ile Glu Glu Asp Ser Ser Val Phe Ala Gln Ser Ile Pro Stp	Substitution, Trp>Stp	Nonsense Mutation, Knockout	3,829
TACATCGAGGAGGACTCCTCTGTCTTTGCCCAGAGCATCCCGTTAAACCTGGAGCGGATTACCCCTC	2 380	Tyr Ile Glu Glu Asp Ser Ser Val Phe Ala Gln Ser Ile Pro Leu Asn Leu Glu Arg Ile Thr Pro	Substitution, Trp>Leu	Missense Mutation	0,692
TACATCGAGGAGGACTCCTCTGTCTTTGCCCAGAGCATCCCGTGTAACCTGGAGCGGATTACCCCTC	2091	Tyr Ile Glu Glu Asp Ser Ser Val Phe Ala Gln Ser Ile Pro Cys Asn Leu Glu Arg Ile Thr Pro	Substitution, Trp>Cys	Missense Mutation	0,608
TACATCGAGGAGGACTCCTCTGTCTTTGCCCAGAGCATCCCATAAAACCTGGAGCGGATTACCCCTC	1 750	Tyr Ile Glu Glu Asp Ser Ser Val Phe Ala Gln Ser Ile Pro Stp	Substitution, Trp>Stp	Nonsense Mutation, Knockout	0,509
TACATCGAGGAGGACTCCTCTGTCTTTGCCCAGAGCATCCCGTGGAACCTGGAGCGGATTACCACTC	1 443	Tyr Ile Glu Glu Asp Ser Ser Val Phe Ala Gln Ser Ile Pro Trp Asn Leu Glu Arg Ile Thr Thr	Substitution, Pro>Thr	Missense Mutation	0,420
TACATCGAGGAGGACTCCTCTGTCTTTGCCCAGAGCATCCAGTGGAACCTGGAGCGGATTACCCCTC	1 296	Tyr Ile Glu Glu Asp Ser Ser Val Phe Ala Gln Ser Ile Gln Trp Asn Leu Glu Arg Ile Thr Pro	Substitution, Pro>Gln	Missense Mutation	0,377
TACATCGAGGAGGACTCCTCTGTCTTTGCCCAGAGCATCCCGTGGAACCTGGAGCGGATTACCCATC	1 217	Tyr Ile Glu Glu Asp Ser Ser Val Phe Ala Gln Ser Ile Pro Trp Asn Leu Glu Arg Ile Thr His	Substitution, Pro>His	Missense Mutation	0,354
TACATCGAGGAGGACTCCTCTGTCTTTGCCAAGAGCATCCCGTGGAACCTGGAGCGGATTACCCCTC	1 080	Tyr Ile Glu Glu Asp Ser Ser Val Phe Ala Lys Ser Ile Pro Trp Asn Leu Glu Arg Ile Thr Pro	Substitution, Gln>Lys	Missense Mutation	0,314

BE3 (#1_ *mPcsk9*)

Alleles	#Reads	Protein Sequence	Type	Outcome	Percentage
ATTGAGGAAGACTCCTTTGTCTTCGCCCAGAGCATCCCATGGAACCTGGAGCGAATTATCC	779 048	Ile Glu Glu Asp Ser Phe Val Phe Ala Gln Ser Ile Pro Trp Asn Leu Glu Arg Ile Ile	Wild type	Wild type	98,217
ATTGAGGAAGACTCCTTTGTCTTC**A**CCCAGAGCATCCCATGGAACCTGGAGCGAATTATCC	374	Ile Glu Glu Asp Ser Phe Val Phe Thr Gln Ser Ile Pro Trp Asn Leu Glu Arg Ile Ile	Substitution, Ala>Thr	Missense Mutation	0,047
ATTGAGGAAGACTCCTTTGTCTTCGCCCAGA**A**CATCCCATGGAACCTGGAGCGAATTATCC	370	Ile Glu Glu Asp Ser Phe Val Phe Ala Gln Asn Ile Pro Trp Asn Leu Glu Arg Ile Ile	Substitution, Ser>Asn	Missense Mutation	0,047
ATTGAGGAAGACTCCTTTGTCTTCGCCCAGAGCATCCCATGGAACCTGGAGC**A**AATTATCC	366	Ile Glu Glu Asp Ser Phe Val Phe Ala Gln Ser Ile Pro Trp Asn Leu Glu Gln Ile Ile	Substitution, Arg>Gln	Missense Mutation	0,046
ATTGAGGAAGACTCCTTTGTCTTCGCCCAGAGCATCCCATGGAACCTGGAG**T**GAATTATCC	251	Ile Glu Glu Asp Ser Phe Val Phe Ala Gln Ser Ile Pro Trp Asn Leu Glu **Stp**	Substitution, Arg>Stp	Nonsense Mutation, Knockout	0,032
ATTGAGGAAGACTCCTTTGTCTTCGCCCAGAG**T**ATCCCATGGAACCTGGAGCGAATTATCC	218	Ile Glu Glu Asp Ser Phe Val Phe Ala Gln Ser Ile Pro Trp Asn Leu Glu Arg Ile Ile	Substitution	Silent Mutation	0,027
ATTGAGGAAGACTCCTTTG**A**CTTCGCCCAGAGCATCCCATGGAACCTGGAGCGAATTATCC	212	Ile Glu Glu Asp Ser Phe Asp Phe Ala Gln Ser Ile Pro Trp Asn Leu Glu Arg Ile Ile	Substitution, Val>Asp	Missense Mutation	0,027
ATTGAGGAAGACTCCTTTGTCTTCGCCCAGAGCATCCCATGGAACC**C**GGAGCGAATTATCC	179	Ile Glu Glu Asp Ser Phe Val Phe Ala Gln Ser Ile Pro Trp Asn Pro Glu Arg Ile Ile	Substitution, Leu>Pro	Missense Mutation	0,023
ATTGAGGAAGACTCCTTTGTC**A**TCGCCCAGAGCATCCCATGGAACCTGGAGCGAATTATCC	161	Ile Glu Glu Asp Ser Phe Val Ile Ala Gln Ser Ile Pro Trp Asn Leu Glu Arg Ile Ile	Substitution, Phe>Ile	Missense Mutation	0,020

BE3 (#2_ *mPcsk9*)

Alleles	#Reads	Protein Sequence	Type	Outcome	Percentage
ATTGAGGAAGACTCCTTTGTCTTCGCCCAGAGCATCCCATGGAACCTGGAGCGAATTATCC	372 474	Ile Glu Glu Asp Ser Phe Val Phe Ala Gln Ser Ile Pro Trp Asn Leu Glu Arg Ile Ile	Wild type	Wild type	98,271
ATTGAGGAAGACTCCTTTGTCTTCGCCCAGAGCATCCCATGGAACCTGGAG**T**GAATTATCC	178	Ile Glu Glu Asp Ser Phe Val Phe Ala Gln Ser Ile Pro Trp Asn Leu Glu **Stp**	Substitution, Arg>Stp	Nonsense Mutation, Knockout	0,047
ATTGAGGAAGACTCCTTTGTCTTCGCCCAGA**A**CATCCCATGGAACCTGGAGCGAATTATCC	153	Ile Glu Glu Asp Ser Phe Val Phe Ala Gln Asn Ile Pro Trp Asn Leu Glu Arg Ile Ile	Substitution, Ser>Asn	Missense Mutation	0,040
ATTGAGGAAGACTCCTTTGTCTTC**A**CCCAGAGCATCCCATGGAACCTGGAGCGAATTATCC	150	Ile Glu Glu Asp Ser Phe Val Phe Thr Gln Ser Ile Pro Trp Asn Leu Glu Arg Ile Ile	Substitution, Ala>Thr	Missense Mutation	0,040
ATTGAGGAAGACTCCTTTGTCTTCGCCCAGAG**T**ATCCCATGGAACCTGGAGCGAATTATCC	130	Ile Glu Glu Asp Ser Phe Val Phe Ala Gln Ser Ile Pro Trp Asn Leu Glu Arg Ile Ile	Substitution	Silent Mutation	0,034
ATTGAGGAAGACTCCTTTG**A**CTTCGCCCAGAGCATCCCATGGAACCTGGAGCGAATTATCC	95	Ile Glu Glu Asp Ser Phe Asp Phe Ala Gln Ser Ile Pro Trp Asn Leu Glu Arg Ile Ile	Substitution, Val>Asp	Missense Mutation	0,025
ATTGAGGAAGACTCCTTTGTCTTCGCCCAGAGCATCCCATGGAACCTGGAGC**A**AATTATCC	93	Ile Glu Glu Asp Ser Phe Val Phe Ala Gln Ser Ile Pro Trp Asn Leu Glu Gln Ile Ile	Substitution, Arg>Gln	Missense Mutation	0,025
ATTGAGGAAGACTCCTTTGTCTTCGCCCAGAGCATCCCATGGAACC**C**GGAGCGAATTATCC	87	Ile Glu Glu Asp Ser Phe Val Phe Ala Gln Ser Ile Pro Trp Asn Pro Glu Arg Ile Ile	Substitution, Leu>Pro	Missense Mutation	0,023

BE3 (#3_ *mPcsk9*)

Alleles	#Reads	Protein Sequence	Type	Outcome	Percentage
ATTGAGGAAGACTCCTTTGTCTTCGCCCAGAGCATCCCATGGAACCTGGAGCGAATTATCC	885 187	Ile Glu Glu Asp Ser Phe Val Phe Ala Gln Ser Ile Pro Trp Asn Leu Glu Arg Ile Ile	Wild type	Wild type	98,261
ATTGAGGAAGACTCCTTTGTCTTC**A**CCCAGAGCATCCCATGGAACCTGGAGCGAATTATCC	459	Ile Glu Glu Asp Ser Phe Val Phe Thr Gln Ser Ile Pro Trp Asn Leu Glu Arg Ile Ile	Substitution, Ala>Thr	Missense Mutation	0,051
ATTGAGGAAGACTCCTTTGTCTTCGCCCAGA**A**CATCCCATGGAACCTGGAGCGAATTATCC	382	Ile Glu Glu Asp Ser Phe Val Phe Ala Gln Asn Ile Pro Trp Asn Leu Glu Arg Ile Ile	Substitution, Ser>Asn	Missense Mutation	0,042
ATTGAGGAAGACTCCTTTGTCTTCGCCCAGAGCATCCCATGGAACCTGGAG**T**GAATTATCC	297	Ile Glu Glu Asp Ser Phe Val Phe Ala Gln Ser Ile Pro Trp Asn Leu Glu **Stp**	Substitution, Arg>Stp	Nonsense Mutation, Knockout	0,033
ATTGAGGAAGACTCCTTTGTCTTCGCCCAGAG**T**ATCCCATGGAACCTGGAGCGAATTATCC	284	Ile Glu Glu Asp Ser Phe Val Phe Ala Gln Ser Ile Pro Trp Asn Leu Glu Arg Ile Ile	Substitution	Silent Mutation	0,032
ATTGAGGAAGACTCCTTTGTCTTCGCCCAGAGCATCCCATGGAACC**C**GGAGCGAATTATCC	262	Ile Glu Glu Asp Ser Phe Val Phe Ala Gln Ser Ile Pro Trp Asn Pro Glu Arg Ile Ile	Substitution, Leu>Pro	Missense Mutation	0,029
ATTGAGGAAGACTCCTTTG**A**CTTCGCCCAGAGCATCCCATGGAACCTGGAGCGAATTATCC	212	Ile Glu Glu Asp Ser Phe Asp Phe Ala Gln Ser Ile Pro Trp Asn Leu Glu Arg Ile Ile	Substitution, Val>Asp	Missense Mutation	0,024
ATTGAGGAAGACTCC**C**TTGTCTTCGCCCAGAGCATCCCATGGAACCTGGAGCGAATTATCC	208	Ile Glu Glu Asp Ser Leu Val Phe Ala Gln Ser Ile Pro Trp Asn Leu Glu Arg Ile Ile	Substitution, Phe>Leu	Missense Mutation	0,023

BE3 (#4_ *mPcsk9*)

Alleles	#Reads	Protein Sequence	Type	Outcome	Percentage
ATTGAGGAAGACTCCTTTGTCTTCGCCCAGAGCATCCCATGGAACCTGGAGCGAATTATCC	520 784	Ile Glu Glu Asp Ser Phe Val Phe Ala Gln Ser Ile Pro Trp Asn Leu Glu Arg Ile Ile	Wild type	Wild type	98,213
ATTGAGGAAGACTCCTTTGTCTTCGCCCAGAGCATCCCATGGAACCTGGAG**T**GAATTATCC	267	Ile Glu Glu Asp Ser Phe Val Phe Ala Gln Ser Ile Pro Trp Asn Leu Glu **Stp**	Substitution, Arg>Stp	Nonsense Mutation, Knockout	0,050
ATTGAGGAAGACTCCTTTGTCTTCGCCCAGA**A**CATCCCATGGAACCTGGAGCGAATTATCC	251	Ile Glu Glu Asp Ser Phe Val Phe Ala Gln Asn Ile Pro Trp Asn Leu Glu Arg Ile Ile	Substitution, Ser>Asn	Missense Mutation	0,047
ATTGAGGAAGACTCCTTTGTCTTC**A**CCCAGAGCATCCCATGGAACCTGGAGCGAATTATCC	231	Ile Glu Glu Asp Ser Phe Val Phe Thr Gln Ser Ile Pro Trp Asn Leu Glu Arg Ile Ile	Substitution, Ala>Thr	Missense Mutation	0,044
ATTGAGGAAGACTCCTTTGTCTTCGCCCAGAGCATCCCATGGAACC**C**GGAGCGAATTATCC	149	Ile Glu Glu Asp Ser Phe Val Phe Ala Gln Ser Ile Pro Trp Asn Pro Glu Arg Ile Ile	Substitution, Leu>Pro	Missense Mutation	0,028
ATTGAGGAAGACTCCTTTGTCTTCGCCCAGAG**T**ATCCCATGGAACCTGGAGCGAATTATCC	132	Ile Glu Glu Asp Ser Phe Val Phe Ala Gln Ser Ile Pro Trp Asn Leu Glu Arg Ile Ile	Substitution	Silent Mutation	0,025
ATTGAGGAAGACTCCTTTG**A**CTTCGCCCAGAGCATCCCATGGAACCTGGAGCGAATTATCC	132	Ile Glu Glu Asp Ser Phe Asp Phe Ala Gln Ser Ile Pro Trp Asn Leu Glu Arg Ile Ile	Substitution, Val>Asp	Missense Mutation	0,025
ATTGAGGAAGACTCC**C**TTGTCTTCGCCCAGAGCATCCCATGGAACCTGGAGCGAATTATCC	117	Ile Glu Glu Asp Ser Leu Val Phe Ala Gln Ser Ile Pro Trp Asn Leu Glu Arg Ile Ile	Substitution, Phe>Leu	Missense Mutation	0,022
ATTGAGGAAGACTCCTTTGTCTTCGCCCAGAGCATCCCATGGAACCTGGAGC**A**AATTATCC	111	Ile Glu Glu Asp Ser Phe Val Phe Ala Gln Ser Ile Pro Trp Asn Leu Glu Gln Ile Ile	Substitution, Arg>Gln	Missense Mutation	0,021
ATTGAGGAAGACTCCTTTGTCTTCGCCCAG**G**GCATCCCATGGAACCTGGAGCGAATTATCC	97	Ile Glu Glu Asp Ser Phe Val Phe Ala Gln Gly Ile Pro Trp Asn Leu Glu Arg Ile Ile	Substitution, Ser>Gly	Missense Mutation	0,018

BE3 (#5_ *mPcsk9*)

Alleles	#Reads	Protein Sequence	Type	Outcome	Percentage
ATTGAGGAAGACTCCTTTGTCTTCGCCCAGAGCATCCCATGGAACCTGGAGCGAATTATCC	625 878	Ile Glu Glu Asp Ser Phe Val Phe Ala Gln Ser Ile Pro Trp Asn Leu Glu Arg Ile Ile	Wild type	Wild type	98,207
ATTGAGGAAGACTCCTTTGTCTTC**A**CCCAGAGCATCCCATGGAACCTGGAGCGAATTATCC	389	Ile Glu Glu Asp Ser Phe Val Phe **Thr** Gln Ser Ile Pro Trp Asn Leu Glu Arg Ile Ile	Substitution, Ala>Thr	Missense Mutation	0,061
ATTGAGGAAGACTCCTTTGTCTTCGCCCAGAGCATCCCATGGAACCTGGAG**T**GAATTATCC	317	Ile Glu Glu Asp Ser Phe Val Phe Ala Gln Ser Ile Pro Trp Asn Leu Glu **Stp**	Substitution, Arg>Stp	Nonsense Mutation, Knockout	0,050
ATTGAGGAAGACTCCTTTGTCTTCGCCCAGA**A**CATCCCATGGAACCTGGAGCGAATTATCC	280	Ile Glu Glu Asp Ser Phe Val Phe Ala Gln **Asn** Ile Pro Trp Asn Leu Glu Arg Ile Ile	Substitution, Ser>Asn	Missense Mutation	0,044
ATTGAGGAAGACTCCTTTGTCTTCGCCCAGAG**T**ATCCCATGGAACCTGGAGCGAATTATCC	195	Ile Glu Glu Asp Ser Phe Val Phe Ala Gln Ser Ile Pro Trp Asn Leu Glu Arg Ile Ile	Substitution	Silent Mutation	0,031
ATTGAGGAAGACTCCTTTG**A**CTTCGCCCAGAGCATCCCATGGAACCTGGAGCGAATTATCC	193	Ile Glu Glu Asp Ser Phe **Asp** Phe Ala Gln Ser Ile Pro Trp Asn Leu Glu Arg Ile Ile	Substitution, Val>Asp	Missense Mutation	0,030
ATTGAGGAAGACTCC**C**TTGTCTTCGCCCAGAGCATCCCATGGAACCTGGAGCGAATTATCC	138	Ile Glu Glu Asp Ser **Leu** Val Phe Ala Gln Ser Ile Pro Trp Asn Leu Glu Arg Ile Ile	Substitution, Phe>Leu	Missense Mutation	0,022
ATTGAGGAAGACTCCTTTGTCTTCGCCCAGAGCATCCCATGGAACC**C**GGAGCGAATTATCC	136	Ile Glu Glu Asp Ser Phe Val Phe Ala Gln Ser Ile Pro Trp Asn **Pro** Glu Arg Ile Ile	Substitution, Leu>Pro	Missense Mutation	0,021

BE3 (#6_ *mPcsk9*)

Alleles	#Reads	Protein Sequence	Type	Outcome	Percentage
ATTGAGGAAGACTCCTTTGTCTTCGCCCAGAGCATCCCATGGAACCTGGAGCGAATTATCC	656 566	Ile Glu Glu Asp Ser Phe Val Phe Ala Gln Ser Ile Pro Trp Asn Leu Glu Arg Ile Ile	Wild type	Wild type	98,196
ATTGAGGAAGACTCCTTTGTCTTC**A**CCCAGAGCATCCCATGGAACCTGGAGCGAATTATCC	354	Ile Glu Glu Asp Ser Phe Val Phe **Thr** Gln Ser Ile Pro Trp Asn Leu Glu Arg Ile Ile	Substitution, Ala>Thr	Missense Mutation	0,053
ATTGAGGAAGACTCCTTTGTCTTCGCCCAGA**A**CATCCCATGGAACCTGGAGCGAATTATCC	277	Ile Glu Glu Asp Ser Phe Val Phe Ala Gln **Asn** Ile Pro Trp Asn Leu Glu Arg Ile Ile	Substitution, Ser>Asn	Missense Mutation	0,041
ATTGAGGAAGACTCCTTTGTCTTCGCCCAGAGCATCCCATGGAACCTGGAG**T**GAATTATCC	209	Ile Glu Glu Asp Ser Phe Val Phe Ala Gln Ser Ile Pro Trp Asn Leu Glu **Stp**	Substitution, Arg>Stp	Nonsense Mutation, Knockout	0,031
ATTGAGGAAGACTCCTTTGTCTTCGCCCAGAG**T**ATCCCATGGAACCTGGAGCGAATTATCC	199	Ile Glu Glu Asp Ser Phe Val Phe Ala Gln Ser Ile Pro Trp Asn Leu Glu Arg Ile Ile	Substitution	Silent Mutation	0,030
ATTGAGGAAGACTCCTTTG**A**CTTCGCCCAGAGCATCCCATGGAACCTGGAGCGAATTATCC	190	Ile Glu Glu Asp Ser Phe **Asp** Phe Ala Gln Ser Ile Pro Trp Asn Leu Glu Arg Ile Ile	Substitution, Val>Asp	Missense Mutation	0,028
ATTGAGGAAGACTCC**A**TTGTCTTCGCCCAGAGCATCCCATGGAACCTGGAGCGAATTATCC	182	Ile Glu Glu Asp Ser **Ile** Val Phe Ala Gln Ser Ile Pro Trp Asn Leu Glu Arg Ile Ile	Substitution, Phe>Ile	Missense Mutation	0,027
ATTGAGGAAGACTCC**C**TTGTCTTCGCCCAGAGCATCCCATGGAACCTGGAGCGAATTATCC	181	Ile Glu Glu Asp Ser **Leu** Val Phe Ala Gln Ser Ile Pro Trp Asn Leu Glu Arg Ile Ile	Substitution, Phe>Leu	Missense Mutation	0,027
ATTGAGGAAGACTCCTTTGTCTTCGCCCAGAGCATCCCATGGAACC**C**GGAGCGAATTATCC	172	Ile Glu Glu Asp Ser Phe Val Phe Ala Gln Ser Ile Pro Trp Asn **Pro** Glu Arg Ile Ile	Substitution, Leu>Pro	Missense Mutation	0,026
ATTGAGGAAGACTCCTTTGTCTTCGCCCAGAGCATCCCATGGAACCTGGAGC**A**AATTATCC	168	Ile Glu Glu Asp Ser Phe Val Phe Ala Gln Ser Ile Pro Trp Asn Leu Glu **Gln** Ile Ile	Substitution, Arg>Gln	Missense Mutation	0,025

BE3 (#7_ *mPcsk9*)

Alleles	#Reads	Protein Sequence	Type	Outcome	Percentage
ATTGAGGAAGACTCCTTTGTCTTCGCCCAGAGCATCCCATGGAACCTGGAGCGAATTATCC	679 028	Ile Glu Glu Asp Ser Phe Val Phe Ala Gln Ser Ile Pro Trp Asn Leu Glu Arg Ile Ile	Wild type	Wild type	98,177
ATTGAGGAAGACTCCTTTGTCTTC**A**CCCAGAGCATCCCATGGAACCTGGAGCGAATTATCC	369	Ile Glu Glu Asp Ser Phe Val Phe **Thr** Gln Ser Ile Pro Trp Asn Leu Glu Arg Ile Ile	Substitution, Ala>Thr	Missense Mutation	0,053
ATTGAGGAAGACTCCTTTGTCTTCGCCCAGA**A**CATCCCATGGAACCTGGAGCGAATTATCC	317	Ile Glu Glu Asp Ser Phe Val Phe Ala Gln **Asn** Ile Pro Trp Asn Leu Glu Arg Ile Ile	Substitution, Ser>Asn	Missense Mutation	0,046
ATTGAGGAAGACTCCTTTGTCTTCGCCCAGAGCATCCCATGGAACCTGGAG**T**GAATTATCC	307	Ile Glu Glu Asp Ser Phe Val Phe Ala Gln Ser Ile Pro Trp Asn Leu Glu **Stp**	Substitution, Arg>Stp	Nonsense Mutation, Knockout	0,044
ATTGAGGAAGACTCCTTTGTCTTCGCCCAGAG**T**ATCCCATGGAACCTGGAGCGAATTATCC	210	Ile Glu Glu Asp Ser Phe Val Phe Ala Gln Ser Ile Pro Trp Asn Leu Glu Arg Ile Ile	Substitution	Silent Mutation	0,030
ATTGAGGAAGACTCCTTTGTCTTCGCCCAGAGCATCCCATGGAACC**C**GGAGCGAATTATCC	196	Ile Glu Glu Asp Ser Phe Val Phe Ala Gln Ser Ile Pro Trp Asn **Pro** Glu Arg Ile Ile	Substitution, Leu>Pro	Missense Mutation	0,028
ATTGAGGAAGACTCCTTTG**A**CTTCGCCCAGAGCATCCCATGGAACCTGGAGCGAATTATCC	184	Ile Glu Glu Asp Ser Phe **Asp** Phe Ala Gln Ser Ile Pro Trp Asn Leu Glu Arg Ile Ile	Substitution, Val>Asp	Missense Mutation	0,027
ATTGAGGAAGACTCCTTTGTCTT**T**GCCCAGAGCATCCCATGGAACCTGGAGCGAATTATCC	175	Ile Glu Glu Asp Ser Phe Val Phe Ala Gln Ser Ile Pro Trp Asn Leu Glu Arg Ile Ile	Substitution	Silent Mutation	0,025
ATTGAGGAAGACTCCTTTGTCTTCGCCCAGAGCATCCCATGGAACCTGGAGC**A**AATTATCC	169	Ile Glu Glu Asp Ser Phe Val Phe Ala Gln Ser Ile Pro Trp Asn Leu Glu **Gln** Ile Ile	Substitution, Arg>Gln	Missense Mutation	0,024
ATTGAGGAAGACTCC**C**TTGTCTTCGCCCAGAGCATCCCATGGAACCTGGAGCGAATTATCC	147	Ile Glu Glu Asp Ser **Leu** Val Phe Ala Gln Ser Ile Pro Trp Asn Leu Glu Arg Ile Ile	Substitution, Phe>Leu	Missense Mutation	0,021

BE3 (#8_ *mPcsk9*)

Alleles	#Reads	Protein Sequence	Type	Outcome	Percentage
ATTGAGGAAGACTCCTTTGTCTTCGCCCAGAGCATCCCATGGAACCTGGAGCGAATTATCC	423 846	Ile Glu Glu Asp Ser Phe Val Phe Ala Gln Ser Ile Pro Trp Asn Leu Glu Arg Ile Ile	Wild type	Wild type	98,242
ATTGAGGAAGACTCCTTTGTCTTC**A**CCCAGAGCATCCCATGGAACCTGGAGCGAATTATCC	230	Ile Glu Glu Asp Ser Phe Val Phe **Thr** Gln Ser Ile Pro Trp Asn Leu Glu Arg Ile Ile	Substitution, Ala>Thr	Missense Mutation	0,053
ATTGAGGAAGACTCCTTTGTCTTCGCCCAGA**A**CATCCCATGGAACCTGGAGCGAATTATCC	200	Ile Glu Glu Asp Ser Phe Val Phe Ala Gln **Asn** Ile Pro Trp Asn Leu Glu Arg Ile Ile	Substitution, Ser>Asn	Missense Mutation	0,046
ATTGAGGAAGACTCCTTTGTCTTCGCCCAGAG**T**ATCCCATGGAACCTGGAGCGAATTATCC	120	Ile Glu Glu Asp Ser Phe Val Phe Ala Gln Ser Ile Pro Trp Asn Leu Glu Arg Ile Ile	Substitution	Silent Mutation	0,028
ATTGAGGAAGACTCCTTTGTCTTCGCCCAGAGCATCCCATGGAACCTGGAG**T**GAATTATCC	119	Ile Glu Glu Asp Ser Phe Val Phe Ala Gln Ser Ile Pro Trp Asn Leu Glu **Stp**	Substitution, Arg>Stp	Nonsense Mutation, Knockout	0,028
ATTGAGGAAGACTCCTTTGTCTTCGCCCAGAGCATCCCATGGAACCTGGAGC**A**AATTATCC	118	Ile Glu Glu Asp Ser Phe Val Phe Ala Gln Ser Ile Pro Trp Asn Leu Glu **Gln** Ile Ile	Substitution, Arg>Gln	Missense Mutation	0,027
ATTGAGGAAGACTCCTTTG**A**CTTCGCCCAGAGCATCCCATGGAACCTGGAGCGAATTATCC	114	Ile Glu Glu Asp Ser Phe **Asp** Phe Ala Gln Ser Ile Pro Trp Asn Leu Glu Arg Ile Ile	Substitution, Val>Asp	Missense Mutation	0,026
ATTGAGGAAGACTCC**C**TTGTCTTCGCCCAGAGCATCCCATGGAACCTGGAGCGAATTATCC	104	Ile Glu Glu Asp Ser **Leu** Val Phe Ala Gln Ser Ile Pro Trp Asn Leu Glu Arg Ile Ile	Substitution, Phe>Leu	Missense Mutation	0,024
ATTGAGGAAGACTCCTTTGTCTTCGCCCAGAGCATCCCATGGAACC**C**GGAGCGAATTATCC	98	Ile Glu Glu Asp Ser Phe Val Phe Ala Gln Ser Ile Pro Trp Asn **Pro** Glu Arg Ile Ile	Substitution, Leu>Pro	Missense Mutation	0,023

BE3 (#1_ *hPCSK9*)

Alleles	#Reads	Protein Sequence	Type	Outcome	Percentage
TACATCGAGGAGGACTCCTCTGTCTTTGCCCAGAGCATCCCGTGGAACCTGGAGCGGATTACCCCTC	291 098	Tyr Ile Glu Glu Asp Ser Ser Val Phe Ala Gln Ser Ile Pro Trp Asn Leu Glu Arg Ile Thr Pro	Wild type	Wild type	98,358
TGCATCGAGGAGGACTCCTCTGTCTTTGCCCAGAGCATCCCGTGGAACCTGGAGCGGATTACCCCTC	150	Cys Ile Glu Glu Asp Ser Ser Val Phe Ala Gln Ser Ile Pro Trp Asn Leu Glu Arg Ile Thr Pro	Substitution, Tyr>Cys	Missense Mutation	0,051
TACATCGAGGAGGACTCCTCTGTCTTTGCCCAGAGCATCCCGTGGAACCTGGAGTGGATTACCCCTC	114	Tyr Ile Glu Glu Asp Ser Ser Val Phe Ala Gln Ser Ile Pro Trp Asn Leu Glu Trp Ile Thr Pro	Substitution, Arg>Trp	Missense Mutation	0,039
TACATCGAGGAGGACTCCTCTGTCTTTGCCCAGAACATCCCGTGGAACCTGGAGCGGATTACCCCTC	105	Tyr Ile Glu Glu Asp Ser Ser Val Phe Ala Gln Asn Ile Pro Trp Asn Leu Glu Arg Ile Thr Pro	Substitution, Ser>Asn	Missense Mutation	0,035
TACATCGAGGAGGACTCCTCTGTCTTTACCCAGAGCATCCCGTGGAACCTGGAGCGGATTACCCCTC	104	Tyr Ile Glu Glu Asp Ser Ser Val Phe Thr Gln Ser Ile Pro Trp Asn Leu Glu Arg Ile Thr Pro	Substitution, Ala>Thr	Missense Mutation	0,035
TACATCGAGGAGGACTCCTCTGTCTTTGTCCAGAGCATCCCGTGGAACCTGGAGCGGATTACCCCTC	95	Tyr Ile Glu Glu Asp Ser Ser Val Phe Val Gln Ser Ile Pro Trp Asn Leu Glu Arg Ile Thr Pro	Substitution, Ala>Val	Missense Mutation	0,032
TACATCGAGGAGGACTCCTCTGTCTTTGCCCAGAGCATCCCATGGAACCTGGAGCGGATTACCCCTC	73	Tyr Ile Glu Glu Asp Ser Ser Val Phe Ala Gln Ser Ile Pro Trp Asn Leu Glu Arg Ile Thr Pro	Substitution	Silent Mutation	0,025
TACATCGAGGAGGACTCCTCTGTCCTTGCCCAGAGCATCCCGTGGAACCTGGAGCGGATTACCCCTC	68	Tyr Ile Glu Glu Asp Ser Ser Val Leu Ala Gln Ser Ile Pro Trp Asn Leu Glu Arg Ile Thr Pro	Substitution, Phe>Leu	Missense Mutation	0,023
TACATCGAGGAGGACTCCTCCGTCTTTGCCCAGAGCATCCCGTGGAACCTGGAGCGGATTACCCCTC	65	Tyr Ile Glu Glu Asp Ser Ser Val Phe Ala Gln Ser Ile Pro Trp Asn Leu Glu Arg Ile Thr Pro	Substitution	Silent Mutation	0,022
TACATCGAGGAGGACTCCTCTGTCTTTGCCCAGAGCATCCCGTGGAACCCGGAGCGGATTACCCCTC	65	Tyr Ile Glu Glu Asp Ser Ser Val Phe Ala Gln Ser Ile Pro Trp Asn Pro Glu Arg Ile Thr Pro	Substitution, Leu>Pro	Missense Mutation	0,022
TACATCGAGGAGGACTCCTCTGTCTTTGCCCAGAGCATCCCGCGGAACCTGGAGCGGATTACCCCTC	61	Tyr Ile Glu Glu Asp Ser Ser Val Phe Ala Gln Ser Ile Pro Arg Asn Leu Glu Arg Ile Thr Pro	Substitution, Trp>Arg	Missense Mutation	0,021

BE3 (#2_ *hPCSK9*)

Alleles	#Reads	Protein Sequence	Type	Outcome	Percentage
TACATCGAGGAGGACTCCTCTGTCTTTGCCCAGAGCATCCCGTGGAACCTGGAGCGGATTACCCCTC	256 652	Tyr Ile Glu Glu Asp Ser Ser Val Phe Ala Gln Ser Ile Pro Trp Asn Leu Glu Arg Ile Thr Pro	Wild type	Wild type	98,286
TACATCGAGGAGGACTCCTCTGTCTTTGCCCAGAGCATCCCGTGGAACCTGGAGTGGATTACCCCTC	123	Tyr Ile Glu Glu Asp Ser Ser Val Phe Ala Gln Ser Ile Pro Trp Asn Leu Glu Trp Ile Thr Pro	Substitution, Arg>Trp	Missense Mutation	0,047
TACATCGAGGAGGACTCCTCTGTCTTTGCCCAGAACATCCCGTGGAACCTGGAGCGGATTACCCCTC	118	Tyr Ile Glu Glu Asp Ser Ser Val Phe Ala Gln Asn Ile Pro Trp Asn Leu Glu Arg Ile Thr Pro	Substitution, Ser>Asn	Missense Mutation	0,045
TACATCGAGGAGGACTCCTCTGTCTTTACCCAGAGCATCCCGTGGAACCTGGAGCGGATTACCCCTC	88	Tyr Ile Glu Glu Asp Ser Ser Val Phe Thr Gln Ser Ile Pro Trp Asn Leu Glu Arg Ile Thr Pro	Substitution, Ala>Thr	Missense Mutation	0,034
TACATCGAGGAGGACTCCTCTGTCTTTGTCCAGAGCATCCCGTGGAACCTGGAGCGGATTACCCCTC	88	Tyr Ile Glu Glu Asp Ser Ser Val Phe Val Gln Ser Ile Pro Trp Asn Leu Glu Arg Ile Thr Pro	Substitution, Ala>Val	Missense Mutation	0,034
TACATCGAGGAGGACTCCTCTGTCTTTGCCCAGAGCATCCCATGGAACCTGGAGCGGATTACCCCTC	84	Tyr Ile Glu Glu Asp Ser Ser Val Phe Ala Gln Ser Ile Pro Trp Asn Leu Glu Arg Ile Thr Pro	Substitution	Silent Mutation	0,032
TACATCGAGGAGGACTCCTCTGTCTTTGCCCAGAGTATCCCGTGGAACCTGGAGCGGATTACCCCTC	69	Tyr Ile Glu Glu Asp Ser Ser Val Phe Ala Gln Ser Ile Pro Trp Asn Leu Glu Arg Ile Thr Pro	Substitution	Silent Mutation	0,026
TACATCGAGGAGGACTCCTCTGTCTTTGCCCAGAGCATCCCGTGGAACCCGGAGCGGATTACCCCTC	68	Tyr Ile Glu Glu Asp Ser Ser Val Phe Ala Gln Ser Ile Pro Trp Asn Pro Glu Arg Ile Thr Pro	Substitution, Leu>Pro	Missense Mutation	0,026
TACATCGAGGAGGACTCCTCTGTCTTTGCCCAGAGCATCCCGTGGAACCTGGAGCGGATTATCCCTC	66	Tyr Ile Glu Glu Asp Ser Ser Val Phe Ala Gln Ser Ile Pro Trp Asn Leu Glu Arg Ile Ile Pro	Substitution, Thr>Ile	Missense Mutation	0,025
TACATCGAGGAGGACTCCTCTGTCTTTGCCCAGAGCATCCCGCGGAACCTGGAGCGGATTACCCCTC	62	Tyr Ile Glu Glu Asp Ser Ser Val Phe Ala Gln Ser Ile Pro Arg Asn Leu Glu Arg Ile Thr Pro	Substitution, Trp>Arg	Missense Mutation	0,024

BE3 (#3_ *hPCSK9*)

Alleles	#Reads	Protein Sequence	Type	Outcome	Percentage
TACATCGAGGAGGACTCCTCTGTCTTTGCCCAGAGCATCCCGTGGAACCTGGAGCGGATTACCCCTC	184 078	Tyr Ile Glu Glu Asp Ser Ser Val Phe Ala Gln Ser Ile Pro Trp Asn Leu Glu Arg Ile Thr Pro	Wild type	Wild type	98,297
TACATCGAGGAGGACTCCTCTGTCTTTGCCCAGAACATCCCGTGGAACCTGGAGCGGATTACCCCTC	80	Tyr Ile Glu Glu Asp Ser Ser Val Phe Ala Gln Asn Ile Pro Trp Asn Leu Glu Arg Ile Thr Pro	Substitution, Ser>Asn	Missense Mutation	0,043
TACATCGAGGAGGACTCCTCTGTCTTTGTCCAGAGCATCCCGTGGAACCTGGAGCGGATTACCCCTC	75	Tyr Ile Glu Glu Asp Ser Ser Val Phe Val Gln Ser Ile Pro Trp Asn Leu Glu Arg Ile Thr Pro	Substitution, Ala>Val	Missense Mutation	0,040
TACATCGAGGAGGACTCCTCTGTCTTTGCCCAGAGCATCCCGTGGAACCTGGAGTGGATTACCCCTC	69	Tyr Ile Glu Glu Asp Ser Ser Val Phe Ala Gln Ser Ile Pro Trp Asn Leu Glu Trp Ile Thr Pro	Substitution, Arg>Trp	Missense Mutation	0,037
TACATCGAGGAGGACTCCTCTGTCTTTACCCAGAGCATCCCGTGGAACCTGGAGCGGATTACCCCTC	63	Tyr Ile Glu Glu Asp Ser Ser Val Phe Thr Gln Ser Ile Pro Trp Asn Leu Glu Arg Ile Thr Pro	Substitution, Ala>Thr	Missense Mutation	0,034
TACATCGAGGAGGACTCCTCTGTCTTTGCCCAGAGCATCCCATGGAACCTGGAGCGGATTACCCCTC	63	Tyr Ile Glu Glu Asp Ser Ser Val Phe Ala Gln Ser Ile Pro Trp Asn Leu Glu Arg Ile Thr Pro	Substitution	Silent Mutation	0,034
TACATCGAGGAGGGCTCCTCTGTCTTTGCCCAGAGCATCCCGTGGAACCTGGAGCGGATTACCCCTC	46	Tyr Ile Glu Glu Gly Ser Ser Val Phe Ala Gln Ser Ile Pro Trp Asn Leu Glu Arg Ile Thr Pro	Substitution, Asp>Gly	Missense Mutation	0,025
TACATCGAGGAGGACTCCTCTGTCTTTGCCCAGAGCATCCTGTGGAACCTGGAGCGGATTACCCCTC	46	Tyr Ile Glu Glu Asp Ser Ser Val Phe Ala Gln Ser Ile Leu Trp Asn Leu Glu Arg Ile Thr Pro	Substitution, Pro>Leu	Missense Mutation	0,025
TACATCGAGGAGGTCTCCTCTGTCTTTGCCCAGAGCATCCCGTGGAACCTGGAGCGGATTACCCCTC	44	Tyr Ile Glu Glu Val Ser Ser Val Phe Ala Gln Ser Ile Pro Trp Asn Leu Glu Arg Ile Thr Pro	Substitution, Asp>Val	Missense Mutation	0,023
TACATCGAGGAGGACTCCTCTGTCTTTGCCCAGAGCATCCCGTGGAACCCGGAGCGGATTACCCCTC	40	Tyr Ile Glu Glu Asp Ser Ser Val Phe Ala Gln Ser Ile Pro Trp Asn Pro Glu Arg Ile Thr Pro	Substitution, Leu>Pro	Missense Mutation	0,021

BE3 (#4_ *hPCSK9*)

Alleles	#Reads	Protein Sequence	Type	Outcome	Percentage
TACATCGAGGAGGACTCCTCTGTCTTTGCCCAGAGCATCCCGTGGAACCTGGAGCGGATTACCCCTC	283 311	Tyr Ile Glu Glu Asp Ser Ser Val Phe Ala Gln Ser Ile Pro Trp Asn Leu Glu Arg Ile Thr Pro	Wild type	Wild type	98,315
TACATCGAGGAGGACTCCTCTGTCTTTGCCCAGAGCATCCCGTGGAACCTGGAGTGGATTACCCCTC	124	Tyr Ile Glu Glu Asp Ser Ser Val Phe Ala Gln Ser Ile Pro Trp Asn Leu Glu Trp Ile Thr Pro	Substitution, Arg>Trp	Missense Mutation	0,043
TACATCGAGGAGGACTCCTCTGTCTTTACCCAGAGCATCCCGTGGAACCTGGAGCGGATTACCCCTC	123	Tyr Ile Glu Glu Asp Ser Ser Val Phe Thr Gln Ser Ile Pro Trp Asn Leu Glu Arg Ile Thr Pro	Substitution, Ala>Thr	Missense Mutation	0,043
TACATCGAGGAGGACTCCTCTGTCTTTGCCCAGAGCATCCCGTGGAACCTGGAGCGGATTATCCCTC	91	Tyr Ile Glu Glu Asp Ser Ser Val Phe Ala Gln Ser Ile Pro Trp Asn Leu Glu Arg Ile Ile Pro	Substitution, Thr>Ile	Missense Mutation	0,032
TACATCGAGGAGGACTCCTCTGTCTTTGCCCAGAGCATCCCGTGGAACCCGGAGCGGATTACCCCTC	91	Tyr Ile Glu Glu Asp Ser Ser Val Phe Ala Gln Ser Ile Pro Trp Asn Pro Glu Arg Ile Thr Pro	Substitution, Leu>Pro	Missense Mutation	0,032
TACATCGAGGAGGACTCCTCTGTCTTTGTCCAGAGCATCCCGTGGAACCTGGAGCGGATTACCCCTC	89	Tyr Ile Glu Glu Asp Ser Ser Val Phe Val Gln Ser Ile Pro Trp Asn Leu Glu Arg Ile Thr Pro	Substitution, Ala>Val	Missense Mutation	0,031
TACATCGAGGAGGACTCCTCTGTCTTTGCCCAGAACATCCCGTGGAACCTGGAGCGGATTACCCCTC	89	Tyr Ile Glu Glu Asp Ser Ser Val Phe Ala Gln Asn Ile Pro Trp Asn Leu Glu Arg Ile Thr Pro	Substitution, Ser>Asn	Missense Mutation	0,031
TACATCGAGGAGGACTCCTCTGTCTTTGCCCAGAGTATCCCGTGGAACCTGGAGCGGATTACCCCTC	85	Tyr Ile Glu Glu Asp Ser Ser Val Phe Ala Gln Ser Ile Pro Trp Asn Leu Glu Arg Ile Thr Pro	Substitution	Silent Mutation	0,029
TACATCGAGGAGGACTCCTCTGTCTTTGCCCAGAGCATCCCATGGAACCTGGAGCGGATTACCCCTC	78	Tyr Ile Glu Glu Asp Ser Ser Val Phe Ala Gln Ser Ile Pro Trp Asn Leu Glu Arg Ile Thr Pro	Substitution	Silent Mutation	0,027
TACATCGAGGAGGACTCCTCTGTCTTTGCCCGGAGCATCCCGTGGAACCTGGAGCGGATTACCCCTC	70	Tyr Ile Glu Glu Asp Ser Ser Val Phe Ala Arg Ser Ile Pro Trp Asn Leu Glu Arg Ile Thr Pro	Substitution, Gln>Arg	Missense Mutation	0,024

BE3 (#5_ *hPCSK9*)

Alleles	#Reads	Protein Sequence	Type	Outcome	Percentage
TACATCGAGGAGGACTCCTCTGTCTTTGCCCAGAGCATCCCGTGGAACCTGGAGCGGATTACCCCTC	311 156	Tyr Ile Glu Glu Asp Ser Ser Val Phe Ala Gln Ser Ile Pro Trp Asn Leu Glu Arg Ile Thr Pro	Wild type	Wild type	97,928
TACATCGAGGAGGACTCCTCTGTCTTTGCCCAGA**A**CATCCCGTGGAACCTGGAGCGGATTACCCCTC	161	Tyr Ile Glu Glu Asp Ser Ser Val Phe Ala Gln Asn Ile Pro Trp Asn Leu Glu Arg Ile Thr Pro	Substitution, Ser>Asn	Missense Mutation	0,051
TACATCGAGGAGGACTCCTCTGTCTTTGCCCAGAGCATCCCGTGGAACCTGGAG**T**GGATTACCCCTC	147	Tyr Ile Glu Glu Asp Ser Ser Val Phe Ala Gln Ser Ile Pro Trp Asn Leu Glu Trp Ile Thr Pro	Substitution, Arg>Trp	Missense Mutation	0,046
TACATCGAGGAGGACTCCTCTGTCTTTG**T**CCAGAGCATCCCGTGGAACCTGGAGCGGATTACCCCTC	128	Tyr Ile Glu Glu Asp Ser Ser Val Phe Val Gln Ser Ile Pro Trp Asn Leu Glu Arg Ile Thr Pro	Substitution, Ala>Val	Missense Mutation	0,040
TACATCGAGGAGGACTCCTCTGTCTTT**A**CCCAGAGCATCCCGTGGAACCTGGAGCGGATTACCCCTC	112	Tyr Ile Glu Glu Asp Ser Ser Val Phe Thr Gln Ser Ile Pro Trp Asn Leu Glu Arg Ile Thr Pro	Substitution, Ala>Thr	Missense Mutation	0,035
TACATCGAGGAGGACTCCTCTGTCTTTGCCCAGAGCATCCC**A**TGGAACCTGGAGCGGATTACCCCTC	107	Tyr Ile Glu Glu Asp Ser Ser Val Phe Ala Gln Ser Ile Pro Trp Asn Leu Glu Arg Ile Thr Pro	Substitution	Silent Mutation	0,034
TACATCGAGGAGGACTCCTCTGTCTTTGCCCAGAG**T**ATCCCGTGGAACCTGGAGCGGATTACCCCTC	103	Tyr Ile Glu Glu Asp Ser Ser Val Phe Ala Gln Ser Ile Pro Trp Asn Leu Glu Arg Ile Thr Pro	Substitution	Silent Mutation	0,032
TACATCGAGGAGG**T**CTCCTCTGTCTTTGCCCAGAGCATCCCGTGGAACCTGGAGCGGATTACCCCTC	97	Tyr Ile Glu Glu Val Ser Ser Val Phe Ala Gln Ser Ile Pro Trp Asn Leu Glu Arg Ile Thr Pro	Substitution, Asp>Val	Missense Mutation	0,031
TACATCGAGGAGGACTCCTCTGTCTTTGCCCAGAGCATCCCGTGGAACC**C**GGAGCGGATTACCCCTC	96	Tyr Ile Glu Glu Asp Ser Ser Val Phe Ala Gln Ser Ile Pro Trp Asn Pro Glu Arg Ile Thr Pro	Substitution, Leu>Pro	Missense Mutation	0,030
TACATCGAGGAGGACTCCTCTGTCTTTGCCCAGAGCATCCCGTGGAACCTGGAGCGGATTA**T**CCCTC	94	Tyr Ile Glu Glu Asp Ser Ser Val Phe Ala Gln Ser Ile Pro Trp Asn Leu Glu Arg Ile Ile Pro	Substitution, Thr>Ile	Missense Mutation	0,030
TACATCGAGGAGGACTCCTCTGTCTTTGCCCAGAGCATCCCGTGGA**T**CCTGGAGCGGATTACCCCTC	92	Tyr Ile Glu Glu Asp Ser Ser Val Phe Ala Gln Ser Ile Pro Trp Ile Leu Glu Arg Ile Thr Pro	Substitution, Asn>Ile	Missense Mutation	0,029

BE3 (#6_ *hPCSK9*)

Alleles	#Reads	Protein Sequence	Type	Outcome	Percentage
TACATCGAGGAGGACTCCTCTGTCTTTGCCCAGAGCATCCCGTGGAACCTGGAGCGGATTACCCCTC	427 337	Tyr Ile Glu Glu Asp Ser Ser Val Phe Ala Gln Ser Ile Pro Trp Asn Leu Glu Arg Ile Thr Pro	Wild type	Wild type	97,862
TACATCGAGGAGGACTCCTCTGTCTTTGCCCAGAGCATCCCGTGGAACCTGGAG**T**GGATTACCCCTC	220	Tyr Ile Glu Glu Asp Ser Ser Val Phe Ala Gln Ser Ile Pro Trp Asn Leu Glu Trp Ile Thr Pro	Substitution, Arg>Trp	Missense Mutation	0,050
TACATCGAGGAGGACTCCTCTGTCTTTGCCCAGAGCATCCC**A**TGGAACCTGGAGCGGATTACCCCTC	215	Tyr Ile Glu Glu Asp Ser Ser Val Phe Ala Gln Ser Ile Pro Trp Asn Leu Glu Arg Ile Thr Pro	Substitution	Silent Mutation	0,049
TACATCGAGGAGGACTCCTCTGTCTTTGCCCAGA**A**CATCCCGTGGAACCTGGAGCGGATTACCCCTC	210	Tyr Ile Glu Glu Asp Ser Ser Val Phe Ala Gln Asn Ile Pro Trp Asn Leu Glu Arg Ile Thr Pro	Substitution, Ser>Asn	Missense Mutation	0,048
TACATCGAGGAGGACTCCTCTGTCTTTG**T**CCAGAGCATCCCGTGGAACCTGGAGCGGATTACCCCTC	201	Tyr Ile Glu Glu Asp Ser Ser Val Phe Val Gln Ser Ile Pro Trp Asn Leu Glu Arg Ile Thr Pro	Substitution, Ala>Val	Missense Mutation	0,046
TACATCGAGGAGGACTCCTCTGTCTTT**A**CCCAGAGCATCCCGTGGAACCTGGAGCGGATTACCCCTC	198	Tyr Ile Glu Glu Asp Ser Ser Val Phe Thr Gln Ser Ile Pro Trp Asn Leu Glu Arg Ile Thr Pro	Substitution, Ala>Thr	Missense Mutation	0,045
TACATCGAGGAGGACTCCTCTGTCTTTGCCCAGAGCATCCCGTGGAACCTGGAGCGGATTA**T**CCCTC	161	Tyr Ile Glu Glu Asp Ser Ser Val Phe Ala Gln Ser Ile Pro Trp Asn Leu Glu Arg Ile Ile Pro	Substitution, Thr>Ile	Missense Mutation	0,037
TACATCGAGGAGGACTCCTCTGTCTTTGCCCAGAGCATCCCGTGGA**T**CCTGGAGCGGATTACCCCTC	144	Tyr Ile Glu Glu Asp Ser Ser Val Phe Ala Gln Ser Ile Pro Trp Ile Leu Glu Arg Ile Thr Pro	Substitution, Asn>Ile	Missense Mutation	0,033
TACATCGAGGAGGACTCCTCTGTCTTTGCCCAGAG**T**ATCCCGTGGAACCTGGAGCGGATTACCCCTC	125	Tyr Ile Glu Glu Asp Ser Ser Val Phe Ala Gln Ser Ile Pro Trp Asn Leu Glu Arg Ile Thr Pro	Substitution	Silent Mutation	0,029
TACATCGAGGAGGACTCCTCTGTCTTTGCCCAGAGCATCC**T**GTGGAACCTGGAGCGGATTACCCCTC	124	Tyr Ile Glu Glu Asp Ser Ser Val Phe Ala Gln Ser Ile Leu Trp Asn Leu Glu Arg Ile Thr Pro	Substitution, Pro>Leu	Missense Mutation	0,028

BE3 (#7_ *hPCSK9*)

Alleles	#Reads	Protein Sequence	Type	Outcome	Percentage
TACATCGAGGAGGACTCCTCTGTCTTTGCCCAGAGCATCCCGTGGAACCTGGAGCGGATTACCCCTC	332 314	Tyr Ile Glu Glu Asp Ser Ser Val Phe Ala Gln Ser Ile Pro Trp Asn Leu Glu Arg Ile Thr Pro	Wild type	Wild type	97,844
TACATCGAGGAGGACTCCTCTGTCTTTGCCCAGAGCATCCCGTGGAACCTGGAGC**T**GATTACCCCTC	235	Tyr Ile Glu Glu Asp Ser Ser Val Phe Ala Gln Ser Ile Pro Trp Asn Leu Glu Leu Ile Thr Pro	Substitution, Arg>Leu	Missense Mutation	0,069
TACATCGAGGAGGACTCCTCTGTCTTTGCCCAGAGCATCCCGTGGAACCTGGAG**T**GGATTACCCCTC	165	Tyr Ile Glu Glu Asp Ser Ser Val Phe Ala Gln Ser Ile Pro Trp Asn Leu Glu Trp Ile Thr Pro	Substitution, Arg>Trp	Missense Mutation	0,049
TACATCGAGGAGGACTCCTCTGTCTTTG**T**CCAGAGCATCCCGTGGAACCTGGAGCGGATTACCCCTC	145	Tyr Ile Glu Glu Asp Ser Ser Val Phe Val Gln Ser Ile Pro Trp Asn Leu Glu Arg Ile Thr Pro	Substitution, Ala>Val	Missense Mutation	0,043
TACATCGAGGAGGACTCCTCTGTCTTT**A**CCCAGAGCATCCCGTGGAACCTGGAGCGGATTACCCCTC	142	Tyr Ile Glu Glu Asp Ser Ser Val Phe Thr Gln Ser Ile Pro Trp Asn Leu Glu Arg Ile Thr Pro	Substitution, Ala>Thr	Missense Mutation	0,042
TACATCGAGGAGGACTCCTCTGTCTTTGCCCAGA**A**CATCCCGTGGAACCTGGAGCGGATTACCCCTC	135	Tyr Ile Glu Glu Asp Ser Ser Val Phe Ala Gln Asn Ile Pro Trp Asn Leu Glu Arg Ile Thr Pro	Substitution, Ser>Asn	Missense Mutation	0,040
TACATCGAGGAGGACTCCTCTGTCTTTGCCCAGAGCATCCCGTGGAACCTGGAGCGGATTA**T**CCCTC	120	Tyr Ile Glu Glu Asp Ser Ser Val Phe Ala Gln Ser Ile Pro Trp Asn Leu Glu Arg Ile Ile Pro	Substitution, Thr>Ile	Missense Mutation	0,035
TACATCGAGGAGGACTCCTCTGTCTTTGCCCAGAGCATCCCGTGGAACCTGGAGC**A**GATTACCCCTC	118	Tyr Ile Glu Glu Asp Ser Ser Val Phe Ala Gln Ser Ile Pro Trp Asn Leu Glu Gln Ile Thr Pro	Substitution, Arg>Gln	Missense Mutation	0,035
TACATCGAGGAGGACTCCTCTGTCTTTGCCCAGAG**T**ATCCCGTGGAACCTGGAGCGGATTACCCCTC	115	Tyr Ile Glu Glu Asp Ser Ser Val Phe Ala Gln Ser Ile Pro Trp Asn Leu Glu Arg Ile Thr Pro	Substitution	Silent Mutation	0,034
TACATCGAGGAGGACTCCTCTGTCTTTGCCCAGAGCATCCC**A**TGGAACCTGGAGCGGATTACCCCTC	112	Tyr Ile Glu Glu Asp Ser Ser Val Phe Ala Gln Ser Ile Pro Trp Asn Leu Glu Arg Ile Thr Pro	Substitution	Silent Mutation	0,033

BE3 (#8_ *hPCSK9*)

Alleles	#Reads	Protein Sequence	Type	Outcome	Percentage
TACATCGAGGAGGACTCCTCTGTCTTTGCCCAGAGCATCCCGTGGAACCTGGAGCGGATTACCCCTC	422 495	Tyr Ile Glu Glu Asp Ser Ser Val Phe Ala Gln Ser Ile Pro Trp Asn Leu Glu Arg Ile Thr Pro	Wild type	Wild type	97,895
TACATCGAGGAGGACTCCTCTGTCTTTGCCCAGAGCATCCCGTGGAACCTGGAG**T**GGATTACCCCTC	272	Tyr Ile Glu Glu Asp Ser Ser Val Phe Ala Gln Ser Ile Pro Trp Asn Leu Glu Trp Ile Thr Pro	Substitution, Arg>Trp	Missense Mutation	0,063
TACATCGAGGAGGACTCCTCTGTCTTTGCCCAGA**A**CATCCCGTGGAACCTGGAGCGGATTACCCCTC	188	Tyr Ile Glu Glu Asp Ser Ser Val Phe Ala Gln Asn Ile Pro Trp Asn Leu Glu Arg Ile Thr Pro	Substitution, Ser>Asn	Missense Mutation	0,044
TACATCGAGGAGGACTCCTCTGTCTTT**A**CCCAGAGCATCCCGTGGAACCTGGAGCGGATTACCCCTC	170	Tyr Ile Glu Glu Asp Ser Ser Val Phe Thr Gln Ser Ile Pro Trp Asn Leu Glu Arg Ile Thr Pro	Substitution, Ala>Thr	Missense Mutation	0,039
TACATCGAGGAGGACTCCTCTGTCTTTG**T**CCAGAGCATCCCGTGGAACCTGGAGCGGATTACCCCTC	164	Tyr Ile Glu Glu Asp Ser Ser Val Phe Val Gln Ser Ile Pro Trp Asn Leu Glu Arg Ile Thr Pro	Substitution, Ala>Val	Missense Mutation	0,038
TACATCGAGGAGGACTCCTCTGTCTTTGCCCAGAGCATCCC**A**TGGAACCTGGAGCGGATTACCCCTC	138	Tyr Ile Glu Glu Asp Ser Ser Val Phe Ala Gln Ser Ile Pro Trp Asn Leu Glu Arg Ile Thr Pro	Substitution	Silent Mutation	0,032
TACATCGAGGAGGACTCCTCTGTCTTTGCCCAGAGCATCCCGTGGAACCTGGAGCGGATTA**T**CCCTC	136	Tyr Ile Glu Glu Asp Ser Ser Val Phe Ala Gln Ser Ile Pro Trp Asn Leu Glu Arg Ile Ile Pro	Substitution, Thr>Ile	Missense Mutation	0,032
TACATCGAGGAGGACTCCTCTGTCTTTGCCCAGAGCATCCCGTGGA**T**CCTGGAGCGGATTACCCCTC	131	Tyr Ile Glu Glu Asp Ser Ser Val Phe Ala Gln Ser Ile Pro Trp Ile Leu Glu Arg Ile Thr Pro	Substitution, Asn>Ile	Missense Mutation	0,030
TACATCGAGGAGG**T**CTCCTCTGTCTTTGCCCAGAGCATCCCGTGGAACCTGGAGCGGATTACCCCTC	131	Tyr Ile Glu Glu Val Ser Ser Val Phe Ala Gln Ser Ile Pro Trp Asn Leu Glu Arg Ile Thr Pro	Substitution, Asp>Val	Missense Mutation	0,030
TACATCGAGGAGGACTCCTCTGTCTTTGCCCAGAG**T**ATCCCGTGGAACCTGGAGCGGATTACCCCTC	123	Tyr Ile Glu Glu Asp Ser Ser Val Phe Ala Gln Ser Ile Pro Trp Asn Leu Glu Arg Ile Thr Pro	Substitution	Silent Mutation	0,029
