# Supplementary material for: In vivo genome and base editing of a human PCSK9 knock-in hypercholesterolemic mouse model
Source: BMC Biol. 2019 Jan 15;17:4. doi: 10.1186/s12915-018-0624-2 (PMC6334452; doi:10.1186/s12915-018-0624-2)
Supplement: Supplementary file 11 — Table S5. Frequency of targeted stop codons generated by BE3-gMH. (PDF 179 kb) [file 12915_2018_624_MOESM11_ESM.pdf]

Additional file 11: Table S5

Frequency of targeted stop codons generated by BE3-gMH.

Treatment	Locus	Mouse ID	Editing (absolute %)	Targeted stop codons (absolute %)	Targeted stop codons (relative %)
BE3-gMH	*Pcsk9*	#1	5,53	2,66	48,10
BE3-gMH	*Pcsk9*	#2	6,74	3,75	55,64
BE3-gMH	*Pcsk9*	#3	11,6	7,3	62,93
BE3-gMH	*Pcsk9*	#4	10,3	6,13	59,51
BE3-gMH	*Pcsk9*	#5	10,3	4,6	44,66
BE3-gMH	*Pcsk9*	#6	11,1	7,2	64,86
BE3-gMH	*Pcsk9*	#7	14,4	10,7	74,31
BE3-gMH	*Pcsk9*	#8	11,9	6,1	51,26
		Average	***10,2***	***6,1***	***57,7***

BE3-gMH	*PCSK9*	#1	11,11	7,02	63,19
BE3-gMH	*PCSK9*	#2	14,41	9,8	68,01
BE3-gMH	*PCSK9*	#3	21,11	14,18	67,17
BE3-gMH	*PCSK9*	#4	24,08	17,3	71,84
BE3-gMH	*PCSK9*	#5	25,13	7,7	30,64
BE3-gMH	*PCSK9*	#6	25,05	14,4	57,49
BE3-gMH	*PCSK9*	#7	34,93	22,4	64,13
BE3-gMH	*PCSK9*	#8	28,29	14,6	51,61
		Average	***23,0***	***13,4***	***58,33***
