# Supplementary material for: In vivo genome and base editing of a human PCSK9 knock-in hypercholesterolemic mouse model
Source: BMC Biol. 2019 Jan 15;17:4. doi: 10.1186/s12915-018-0624-2 (PMC6334452; doi:10.1186/s12915-018-0624-2)
Supplement: Supplementary file 12 — Table S6. List of off-target sites for gMH. (PDF 439 kb) [file 12915_2018_624_MOESM12_ESM.pdf]

Additional file 12: Table S6

List of off-target sites for gMH.

Off-target ID	Sequence	Mismatches	PAM	Locus	Source
OT1	CAGTTTCCATGGGGTGCTCTTGG	2	NGG	chr4:106457249-106457272	GUIDE-Seq
OT6	CAGATTCCTAGGATGCTCTGAG	3	GAG	chr12:111733417-111733439	GUIDE-Seq
OT8	CAGTTTCCATGGAATGTTCTAGG	3	NGG	chr5:98601716-98601739	GUIDE-Seq
OT10	CAGCTTCCATAGGATGCTGTAGG	3	NGG	chr6:6533795-6533818	GUIDE-Seq
OT14	CAGATTCCATGTGATGCTTTAGG	3	NGG	chr16:8681106-8681129	GUIDE-Seq
OT16	CAGCATCCATGGGATGGTCTGGG	3	NGG	chr15:95000060-95000083	GUIDE-Seq
OT17	CAGGTTTCATGGAGTGCTCTAGG	3	NGG	chr4:93574622-93574645	GUIDE-Seq
OT24	CAGGTTCCAGGGATGCTCTGGA	1	GGA	chr9:118783495-118783517	GUIDE-Seq
OT29	GAGGGTCCATGGGATGCTCTAAG	2	AAG	chr8:26866365-26866388	GUIDE-Seq
OT34	CAGGCTCCTAGGATGCTTTGGG	4	NGG	chr17:8243109-8243131	GUIDE-Seq
OT36	CAGCTTCCTGAGATGCCCTAGG	4	NGG	chr4:108893167-108893189	GUIDE-Seq
OT45	CAGGTTCCATAGGTGGCTCTGGG	3	NGG	chr14:60785005-60785028	GUIDE-Seq
OT58	CAGGGTCCATGGGATGCCCTGAG	2	GAG	chr18:64586045-64586068	GUIDE-Seq
OT68	CAGGTGCCATGGCATGCTCTCAG	2	CAG	chr8:60460964-60460987	GUIDE-Seq
OT70	CAGGTTCCACGGGATGCTCTGGG	1	NGG	hPCSK9-KI	GUIDE-Seq
OT71	CTGGTGCCCTGGGATGCTCTGGG	3	NGG	chr7:140152720-140152743	Bioinformatics
OT73	CAGGTTTCATGGTATGGTCTAGG	3	AGG	chr15:78106085-78106108	Bioinformatics
OT74	CAGGTTCCACGGGCTGCTCTGGG	2	NGG	chr16:18132505-18132528	Bioinformatics
OT75	CAGATTCCATGGGATGCACTGTG	2	GTG	chr16:90528975-90528998	Bioinformatics
OT77	CAGGTTCCTTGTGAGGCTCTTGG	3	TGG	chr17:33501685-33501708	Bioinformatics
OT78	CAGGTTCAATGGGAGGCTCTGGA	2	GGA	chr3:14980359-14980382	Bioinformatics
OT79	CAGGTTCCAGGGGTTGCTCTCAG	2	CAG	chr4:89194835-89194858	Bioinformatics
OT80	CAGGTTGCCTGGGATGCTCTTTG	2	TTG	chr7:54902936-54902959	Bioinformatics
OT81	CAGGTGCCTTGGGATTCTCTTGG	3	TGG	chr9:123273423-123273446	Bioinformatics
