# Supplementary material for: In vivo genome and base editing of a human PCSK9 knock-in hypercholesterolemic mouse model
Source: BMC Biol. 2019 Jan 15;17:4. doi: 10.1186/s12915-018-0624-2 (PMC6334452; doi:10.1186/s12915-018-0624-2)
Supplement: Supplementary file 13 — Table S7. List of primers used in this study. (PDF 215 kb) [file 12915_2018_624_MOESM13_ESM.pdf]

# Additional file 13: Table S7

## List of primers used in this study.

| ID | Sequence | Gene | Species | Application |
|---|---|---|---|---|
| Neo probe Forw | AAGTATCCATCATGGCTGATG | Neo cassette | | ES clone screening |
| Neo probe Rev | TCGTCAAGAAGGCGATAGAAG | | | ES clone screening |
| P1 | CCAGGTCTGGAATGCAAAGT | hPCSK9 | Human | Mouse genotyping |
| P2 | TTTTGATAAGGCTGCAGAAGGAGC | R26 | Mouse | Mouse genotyping |
| P3 | GTGTTCGTGCAAGTTGAGTCCATC | | Mouse | Mouse genotyping |
| mPcsk9-Cel-1Forw | GACTTTGTGAAGGCTGGGGA | Pcsk9 | Mouse | Surveyor |
| mPcsk9-Cel-1Rev | TGCATGGAGCAATGCAGAGA | | Mouse | Surveyor |
| hPcsk9-Cel-1Forw | ACTCAGGCTCCTAGTCTGTCC | PCSK9 | Human | Surveyor |
| hPcsk9-Cel-1Rev | CAGATGGGGGTCTTACCGGG | | Human | Surveyor |
| mPcsk9Forw | CCATGGCCAGCACTGGTATC | Pcsk9 | Mouse | Sanger Sequencing |
| mPcsk9Rev | GGGATGAAGCCAACACGGAT | | Mouse | Sanger Sequencing |
| hPCSK9Forw | GCCTGAGCTTCACTCCATGT | PCSK9 | Human | Sanger Sequencing |
| hPCSK9Rev | GGCCCTAGGTAGAGTCACCA | | Human | Sanger Sequencing |
| mON-Forw | CATGGCTGTCTGGTTCTGTA | Pcsk9 | Mouse | Deep sequencing |
| mON-Rev | AGG TCA GTG CAG ACT CTG GAG | | Mouse | Deep sequencing |
| hON-Forw | ATGTGGGGACAGGTTTGATC | PCSK9 | Human | Deep sequencing |
| hON-Rev | TGGTATTCATCCGCCCGGTA | | Human | Deep sequencing |
| OT1-Forw | GGTTTCCAGGAGCTGAGGACA | OT1 | Mouse | Deep sequencing |
| OT1-Rev | CCCCCTCAGAGCAGTTTTAGGT | | Mouse | Deep sequencing |
| OT6-Forw | GGCGCTACTCATCACTGCCTGT | OT6 | Mouse | Deep sequencing |
| OT6-Rev | CAGTTTCCTGGAGGTGCCACA | | Mouse | Deep sequencing |
| OT8-Forw | TGTGCAGTAAAGAACAACTTTGAGG | OT8 | Mouse | Deep sequencing |
| OT8-Rev | ACTTCTGCCCAGAGGCACC | | Mouse | Deep sequencing |
| OT0-Forw | GGCCTCATGACTTGGGCATCACT | OT10 | Mouse | Deep sequencing |
| OT10-Rev | AGCTCCTGGTGAGTAGCCTCCA | | Mouse | Deep sequencing |
| OT14-Forw | CCCCGATCTCACTGACCCCTA | OT14 | Mouse | Deep sequencing |
| OT14-Rev | ACACCTTCGTTGGCCACCTCAG | | Mouse | Deep sequencing |
| OT16-Forw | TCTCTGACCTCTGATGGCTCAAGT | OT16 | Mouse | Deep sequencing |
| OT16-Rev | GCCTGCAGAGCACATCAGGGAT | | Mouse | Deep sequencing |
| OT17-Forw | ACACACCACAATGTCTCATTCCAGGA | OT17 | Mouse | Deep sequencing |
| OT17-Rev | AGGCCATTTGTACCAGCTAGATACT | | Mouse | Deep sequencing |
| OT24-Forw | GACCACAGTGCTCACATCCTCCT | OT24 | Mouse | Deep sequencing |
| OT24-Rev | TGTTTCTCTCACCGGAGCTGGC | | Mouse | Deep sequencing |
| OT29-Forw | ATTTTGGGACAGGGTTTCTCGCCT | OT29 | Mouse | Deep sequencing |
| OT29-Rev | GGTAAGGTGAAGTCAGAGGACACA | | Mouse | Deep sequencing |
| OT34-Forw | GGGGGAGACAGACCAGGCATAC | OT34 | Mouse | Deep sequencing |
| OT34-Rev | ACACACCACTCCTCCCCCTAGG | | Mouse | Deep sequencing |
| OT36-Forw | GGCTCTTGGGTCCCCTTGCAAG | OT36 | Mouse | Deep sequencing |
| OT36-Rev | TCGTGAGTGGAGGGATCCTGCT | | Mouse | Deep sequencing |
| OT45-Forw | AGTACTAGCTGGTCCCCGGTGG | OT45 | Mouse | Deep sequencing |
| OT45-Rev | GGAGCTTGAGTAGAGGAAAGGC | | Mouse | Deep sequencing |
| OT58-Forw | TCCGTGTTGTAGCTGTTTGTGA | OT58 | Mouse | Deep sequencing |

| | | | | |
|---|---|---|---|---|
| OT58-Rev | ACCCCCGTTCAAACTCCAGTATGC | | Mouse | Deep sequencing |
| OT68-Forw | TGACACCCACAGCTCAGTTGCT | OT68 | Mouse | Deep sequencing |
| OT68-Rev | TTGCGTTGATGTGTGTGCGTTT | | Mouse | Deep sequencing |
| OT70-Forw | CCTGCAGGCCCAGGCTGC | OT70 | Mouse | Deep sequencing |
| OT70-Rev | TCCGCCCGGTACCGTGGA | | Mouse | Deep sequencing |
| OT71-Forw | ATCATTGGGACCTGCTCTGGGC | OT71 | Mouse | Deep sequencing |
| OT71-Rev | CCCACCCTGACACGAGGACATG | | Mouse | Deep sequencing |
| OT73-Forw | AGGGCAGAGGGAAAGCACTGTG | OT73 | Mouse | Deep sequencing |
| OT73-Rev | GGTTGTGTGCTCCCTTCCTGCT | | Mouse | Deep sequencing |
| OT74-Forw | CGAGTTCTAGGACAGCCAGGGC | OT74 | Mouse | Deep sequencing |
| OT74-Rev | AGACATCTCAGGAGCTCCCCAG | | Mouse | Deep sequencing |
| OT75-Forw | TTCTTGGCTCATGGTTCCGGGG | OT75 | Mouse | Deep sequencing |
| OT75-Rev | AGGTCGATTTGTGAAGCCTGGGT | | Mouse | Deep sequencing |
| OT77-Forw | GGCTCACAACCATCTGAAACCCC | OT77 | Mouse | Deep sequencing |
| OT77-Rev | CATATCCCCATAATTTCTGTGCCTTTTC | | Mouse | Deep sequencing |
| OT78-Forw | CAGCCAGCACTGTAGACTGAACA | OT78 | Mouse | Deep sequencing |
| OT78-Rev | GGCTGATCTACCCCCAATGCCA | | Mouse | Deep sequencing |
| OT79-Forw | TGTTTGGGAGGAAGGGAGGCATA | OT79 | Mouse | Deep sequencing |
| OT79-Rev | GGCGGGTAGGTGGGATCCTAGG | | Mouse | Deep sequencing |
| OT80-Forw | TCTGGCTCTGGAGTCTGTCTACA | OT80 | Mouse | Deep sequencing |
| OT80-Rev | ATCTGGAATTTGGAGGATGGGAC | | Mouse | Deep sequencing |
| OT81-Forw | GGGCTTGGTCACCCCTTTCTC | OT81 | Mouse | Deep sequencing |
| OT81-Rev | TGGACACTCTGCCCAGATAGAT | | Mouse | Deep sequencing |
| Trans-Forw-mouse | GACTTTGTGAAGGCTGGGGA | | Mouse | PCR/Translocations |
| Trans-Rev-human | CATTCTCGAAGTCGGTGACCA | | Human | PCR/Translocations |
